# Supplementary material for: Etiological and epidemiological characteristics of severe acute respiratory infection caused by multiple viruses and Mycoplasma pneumoniae in adult patients in Jinshan, Shanghai: A pilot hospital-based surveillance study
Source: PLoS One. 2021 Mar 22;16(3):e0248750. doi: 10.1371/journal.pone.0248750 (PMC7984646; doi:10.1371/journal.pone.0248750)
Supplement: S1 File — (DOC) [file pone.0248750.s002.doc]

**S1 File.** Sequences of primers targeting Flu A/B used in real-time RT-PCR

| **Virus Type** | **primer** | **Oligonucleotide sequence (5’ to 3’)** |
| --- | --- | --- |
| [2009 H1N1 influenza virus](https://web03.cnki.net/kcms/detail/knetsearch.aspx?dbcode=CDCT_HAMJ&sfield=kw&skey=2009+H1N1+influenza+virus) | pdmH1 | Forward: GGG TAG CCC CAT TGC AT |
|  |  | Reverse: AGA GTG ATT CAC ACT CTG GAT TTC |
| H3N2 influenza virus | CNICH3 | Forward: ACC CTC AGT GTG ATG GCT TTC AAA |
|  |  | Reverse: TAA GGG AGG CAT AAT CCG GCA CAT |
| [Influenza B virus](https://web03.cnki.net/kcms/detail/knetsearch.aspx?dbcode=CDCT_HAMJ&sfield=kw&skey=Influenza+B+virus)（Yamagata lineage and Victoria lineage） | BHA | Forward: AGACCAGAGGGAAACTATGCCC |
|  |  | Reverse: TCCGGATGTAACAGGTCTGACTT |
