# Supplementary material for: Etiological and epidemiological characteristics of severe acute respiratory infection caused by multiple viruses and Mycoplasma pneumoniae in adult patients in Jinshan, Shanghai: A pilot hospital-based surveillance study
Source: PLoS One. 2021 Mar 22;16(3):e0248750. doi: 10.1371/journal.pone.0248750 (PMC7984646; doi:10.1371/journal.pone.0248750)
Supplement: S2 File — (DOCX) [file pone.0248750.s003.docx]

S2 File. STROBE Statement—checklist of items that should be included in reports of observational studies

|  | Item No. | Recommendation | Page  No. | Relevant text from manuscript |
| --- | --- | --- | --- | --- |
| **Title and abstract** | 1 | (*a*) Indicate the study’s design with a commonly used term in the title or the abstract | P1 line3-4 | A pilot hospital-based surveillance study |
|  |  | (*b*) Provide in the abstract an informative and balanced summary of what was done and what was found | 1.P3 line53  2. P3 lne 60-P4 line 73 | 1.Active surveillance was conducted at 1 sentinel hospital.  2. Results section of abstract |
| Introduction | | | |  |
| Background/rationale | 2 | Explain the scientific background and rationale for the investigation being reported | P5 line90-P6 line 116 | Background section |
| Objectives | 3 | State specific objectives, including any prespecified hypotheses | P6 line 116-119 | To characterize the demography and epidemiology of SARI, to identify the etiologies and to assess the clinical profiles of SARI in hospitalized adult patients |
| Methods | | | |  |
| Study design | 4 | Present key elements of study design early in the paper | P6 line 122-123 | Surveillance was piloted at Jinshan district central hospital |
| Setting | 5 | Describe the setting, locations, and relevant dates, including periods of recruitment, exposure, follow-up, and data collection | P6 line 122-125,line 130-131 | Surveillance was piloted at Jinshan district central hospital since April 2017 and lasted for 12 months |
| Participants | 6 | (*a*) *Cohort study*—Give the eligibility criteria, and the sources and methods of selection of participants. Describe methods of follow-up  *Case-control study*—Give the eligibility criteria, and the sources and methods of case ascertainment and control selection. Give the rationale for the choice of cases and controls  *Cross-sectional study*—Give the eligibility criteria, and the sources and methods of selection of participants | P6 line131-P7 line 133 for eligibility criteria, although this is a surveillance study. | Patients were defined as SARI case according to WHO definition if they have acute respiratory infection with measured fever of ≥38˚C, cough, with onset within the last 10 days and require hospitalization |
|  |  | (*b*) *Cohort study*—For matched studies, give matching criteria and number of exposed and unexposed  *Case-control study*—For matched studies, give matching criteria and the number of controls per case | Not applicable |  |
| Variables | 7 | Clearly define all outcomes, exposures, predictors, potential confounders, and effect modifiers. Give diagnostic criteria, if applicable | P7 line135-145 | Data collection section |
| Data sources/ measurement | 8* | For each variable of interest, give sources of data and details of methods of assessment (measurement). Describe comparability of assessment methods if there is more than one group | P7 line135, 143-145. | A standard case report form was completed for each eligible patient. At discharge, the form was updated to include information |
| Bias | 9 | Describe any efforts to address potential sources of bias | Not applicable |  |
| Study size | 10 | Explain how the study size was arrived at | P6 line 130-131 | All patients over 16 years old who were admitted to the sentinel hospital were screened by a trained physician between April 2017 and March 2018. |

Continued on next page

| Quantitative variables | 11 | Explain how quantitative variables were handled in the analyses. If applicable, describe which groupings were chosen and why | P8 line174-175 | The collected data were double-entered into a database constructed by EpiData 3.1. Logical checking for quality of data entry was conducted. |
| --- | --- | --- | --- | --- |
| Statistical methods | 12 | (*a*) Describe all statistical methods, including those used to control for confounding | P9 line177-184 | Chi-squared test, Fisher’s exact test and Mann-Whitney U test, as appropriate, were used to compare patients with and without confirmed pathogen in terms of demographic, clinical, epidemiologic characteristics, treatment and prognosis. For proportions, the binomial 95% confidence-interval was reported. |
|  |  | (*b*) Describe any methods used to examine subgroups and interactions | Not applicable |  |
|  |  | (*c*) Explain how missing data were addressed | Not applicable |  |
|  |  | (*d*) *Cohort study*—If applicable, explain how loss to follow-up was addressed  *Case-control study*—If applicable, explain how matching of cases and controls was addressed  *Cross-sectional study*—If applicable, describe analytical methods taking account of sampling strategy | Not applicable |  |
|  |  | (*e*) Describe any sensitivity analyses | Not applicable |  |
| Results | | | | |
| Participants | 13* | (a) Report numbers of individuals at each stage of study—eg numbers potentially eligible, examined for eligibility, confirmed eligible, included in the study, completing follow-up, and analysed | P9 line187-188 | A total of 397 patients meeting the SARI case definition were admitted to our sentinel site. |
|  |  | (b) Give reasons for non-participation at each stage | Not applicable |  |
|  |  | (c) Consider use of a flow diagram | Not applicable |  |
| Descriptive data | 14* | (a) Give characteristics of study participants (eg demographic, clinical, social) and information on exposures and potential confounders | P9 line189-197 | The median age of patients were 68 years (IQR: 59-78; range: 16 to 99 years) and 194(48.9%) were male. 278 SARI patients (70.0%) had at least one comorbidity. |
|  |  | (b) Indicate number of participants with missing data for each variable of interest | Not applicable |  |
|  |  | (c) *Cohort study*—Summarise follow-up time (eg, average and total amount) | Not applicable |  |
| Outcome data | 15* | *Cohort study*—Report numbers of outcome events or summary measures over time | Not applicable |  |
|  |  | *Case-control study—*Report numbers in each exposure category, or summary measures of exposure | Not applicable |  |
|  |  | *Cross-sectional study—*Report numbers of outcome events or summary measures | P9 line188-189  P10 line199-206 | One or more positive pathogen were detected from 250 patients (63.0%; 95%CI: 58.2-67.7%) |
| Main results | 16 | (*a*) Give unadjusted estimates and, if applicable, confounder-adjusted estimates and their precision (eg, 95% confidence interval). Make clear which confounders were adjusted for and why they were included | Not applicable |  |
|  |  | (*b*) Report category boundaries when continuous variables were categorized | Not applicable |  |
|  |  | (*c*) If relevant, consider translating estimates of relative risk into absolute risk for a meaningful time period | Not applicable |  |

Continued on next page

| Other analyses | 17 | Report other analyses done—eg analyses of subgroups and interactions, and sensitivity analyses | Not applicable |  |
| --- | --- | --- | --- | --- |
| Discussion | | | | |
| Key results | 18 | Summarise key results with reference to study objectives | P16 line349-352 | The current study is the first study surveilling hospitalized adult SARI patients for most respiratory viruses and *M*. *pneumoniae* in Shanghai, and confirms that multiple respiratory pathogens may circulate among the SARI population and vary with the climatic and demographic characteristics. |
| Limitations | 19 | Discuss limitations of the study, taking into account sources of potential bias or imprecision. Discuss both direction and magnitude of any potential bias | P16 line339-347 | First, as a pilot project, this study was enforced at only 1 hospital although this hospital is the biggest hospital in the site, so the finding may have relatively limited generalizability. Second, the result was based on SARI surveillance of a 12-month period, and the burden derived from SARI may not reflect the situation over several years. Finally, the pathogens which were included in this piloting surveillance study did not involve in related respiratory bacterium owing to limited financial support. |
| Interpretation | 20 | Give a cautious overall interpretation of results considering objectives, limitations, multiplicity of analyses, results from similar studies, and other relevant evidence | P16 line349-P17 line355 | Section of conclusion |
| Generalisability | 21 | Discuss the generalisability (external validity) of the study results | P16 line339-342 | As a pilot project, this study was enforced at only 1 hospital although this hospital is the biggest hospital in the site, so the finding may have relatively limited generalizability. Actually, prevalence of each pathogen may differ for regions having different climatic, demographic patterns and access to healthcare. |
| Other information | |  | | |
| Funding | 22 | Give the source of funding and the role of the funders for the present study and, if applicable, for the original study on which the present article is based | P17 line368-371 | This work was supported by the Research Project of Shanghai Municipal Health Commission (201940428) and the Infectious Disease and Epidemiology Project of the 6^th^ Jinshan District Medical Key Specialty Construction (JSZK2019B05). |

*Give information separately for cases and controls in case-control studies and, if applicable, for exposed and unexposed groups in cohort and cross-sectional studies.

**Note:** An Explanation and Elaboration article discusses each checklist item and gives methodological background and published examples of transparent reporting. The STROBE checklist is best used in conjunction with this article (freely available on the Web sites of PLoS Medicine at http://www.plosmedicine.org/, Annals of Internal Medicine at http://www.annals.org/, and Epidemiology at http://www.epidem.com/). Information on the STROBE Initiative is available at www.strobe-statement.org.
