# Supplementary material for: Etiological and epidemiological characteristics of severe acute respiratory infection caused by multiple viruses and Mycoplasma pneumoniae in adult patients in Jinshan, Shanghai: A pilot hospital-based surveillance study
Source: PLoS One. 2021 Mar 22;16(3):e0248750. doi: 10.1371/journal.pone.0248750 (PMC7984646; doi:10.1371/journal.pone.0248750)
Supplement: Attachment — Submitted filename: PONE-D-20-19561.pdf [file pone.0248750.s004.pdf]

PLOS ONE

Etiological and epidemiological characters of severe acute respiratory infection caused by multiple viruses and mycoplasma pneumoniae in adult patients in Jinshan of Shanghai, April 2017 to March 2018: a pilot hospital-based surveillance study

--Manuscript Draft--

Manuscript Number:	PONE-D-20-19561
Article Type:	Research Article
Full Title:	Etiological and epidemiological characters of severe acute respiratory infection caused by multiple viruses and mycoplasma pneumoniae in adult patients in Jinshan of Shanghai, April 2017 to March 2018: a pilot hospital-based surveillance study
Short Title:	Etiological and epidemiological characters of severe acute respiratory infection
Corresponding Author:	JIAN LI<br>Ruijin Hospital, Shanghai Jiao Tong University, School of Medicine<br>Shanghai, CHINA
Keywords:	Severe acute respiratory infection, sentinel surveillance, pathogen, epidemiology
Abstract:	Background<br>Severe acute respiratory infection (SARI) presents a tremendous disease burden worldwide. Available research on active surveillance of hospitalized adult patients suffered from SARI in China was limited. This piloting study aimed to identify etiologies and describe the demographic, epidemiological and clinical profiles of hospitalized SARI-associated patients over 16 years old in Jinshan, Shanghai.<br>Methods<br>Active surveillance was conducted at 1 sentinel hospital in Jinshan district, Shanghai from April 2017 to March 2018. Hospitalized SARI patients over 16 years old were enrolled, the pharyngeal swabs were collected within 24 hours of admission and tested for multiple respiratory viruses (including 18 common viruses) and Mycoplasma pneumoniae with real-time polymerase chain reaction. Demographic, epidemiological and clinical information were obtained from case report forms.<br>Results<br>Of 397 SARI patients enrolled, the median age was 68 years and 194(48.9%) were male . Totally 278(70.0%) had at least one underlying chronic medical conditions . The most frequent symptom was cough (99.2%) and sputum production (88.4%). The median duration of hospitalization was 10 days. A total of 250 patients (63.0%) were identified as at least one positive pathogen infection, of whom 198 (49.9%) were single infection and 52 (13.1%) were multiple infections. The pathogens identified most frequently were Mycoplasma pneumoniae (23.9%, 95/397), followed by adenovirus (11.6%, 46/397), influenza virus A/H3N2 (11.1%, 44/397) and human rhinovirus (8.1%, 32/397). The seasonality of pathogen-confirmed SARI patients conformed to a bimodal shape, with the first peak in summer and the second peak in winter. The statistically significant differences were observed with respect to the proportions of dyspnea, radiographic diagnosis of pneumonia and presence of at least one comorbidity among patients who were single-infected by M . pneumoniae , AdV, HRhV, Flu A/H3N2, Flu A /pH1N1 and Flu B/Yamagata. No significant difference among different age groups was attained with regard to the positive rate of main pathogens.<br>Conclusions<br>Mycoplasma pneumoniae , adenovirus and influenza virus A/H3N2 were the leading pathogens among hospitalized SARI patients aged more than 16 years in Jinshan district, Shanghai. Our finding highlights the importance of sustaining multi-pathogen surveillance of SARI patients in sentinel hospitals, which can provides useful information on etiology, epidemiology, and clinical characteristics.
Order of Authors:	JIAN LI
	Can-Lei Song
	Tang Wang
	Yu-Long Ye

Powered by Editorial Manager® and ProduXion Manager® from Aries Systems Corporation

	Jian-Ru Du
	Shu-Hua Li
	Jian-Min Zhu
**Opposed Reviewers:**	
**Additional Information:**	
**Question**	**Response**

Question	Response
**Financial Disclosure**<br><br>Enter a financial disclosure statement that describes the sources of funding for the work included in this submission. Review the submission guidelines for detailed requirements. View published research articles from *PLOS ONE* for specific examples.<br><br>This statement is required for submission and **will appear in the published article** if the submission is accepted. Please make sure it is accurate.<br><br>**Unfunded studies**<br>Enter: *The author(s) received no specific funding for this work.*<br><br>**Funded studies**<br>Enter a statement with the following details:<br>• Initials of the authors who received each award<br>• Grant numbers awarded to each author<br>• The full name of each funder<br>• URL of each funder website<br>• Did the sponsors or funders play any role in the study design, data collection and analysis, decision to publish, or preparation of the manuscript?<br>• **NO** - Include this sentence at the end of your statement: *The funders had no role in study design, data collection and analysis, decision to publish, or preparation of the manuscript.*<br>• **YES** - Specify the role(s) played.<br><br>* typeset	This work was supported by the Research Project of Shanghai Municipal Health Commission (201940428) for Can-Lei Song and the Infectious Disease and Epidemiology Project of the 6th Jinshan District Medical Key Specialty Construction (JSZK2019B05) for Shu-Hua Li . The funder had no role in study design, data collection and analysis, decision to publish or preparation of manuscript.
**Competing Interests**<br><br>Use the instructions below to enter a competing interest statement for this submission. On behalf of all authors, disclose any competing interests that	The authors have declared that no competing interests exist.

could be perceived to bias this work—acknowledging all financial support and any other relevant financial or non-financial competing interests.<br><br>This statement **will appear in the published article** if the submission is accepted. Please make sure it is accurate. View published research articles from *PLOS ONE* for specific examples.<br><br>**NO authors have competing interests**<br><br>Enter: *The authors have declared that no competing interests exist.*<br><br>**Authors with competing interests**<br><br>Enter competing interest details beginning with this statement:<br><br>*I have read the journal's policy and the authors of this manuscript have the following competing interests: [insert competing interests here]*<br><br>* typeset	
**Ethics Statement**<br><br>Enter an ethics statement for this submission. This statement is required if the study involved:<br><br>• Human participants<br>• Human specimens or tissue<br>• Vertebrate animals or cephalopods<br>• Vertebrate embryos or tissues<br>• Field research<br><br>Write "N/A" if the submission does not require an ethics statement.<br><br>General guidance is provided below. Consult the submission guidelines for detailed instructions. **Make sure that all information entered here is included in the Methods section of the manuscript.**	This study belonged to the part of hospital-based surveillance program of SARI of Shanghai, and approved by the ethical review committee of the Shanghai Municipal Center for Disease Control and Prevention. Written informed consent was obtained from patients or proxies before enrollment.

Powered by Editorial Manager® and ProduXion Manager® from Aries Systems Corporation

**Format for specific study types**<br><br>**Human Subject Research (involving human participants and/or tissue)**<br>• Give the name of the institutional review board or ethics committee that approved the study<br>• Include the approval number and/or a statement indicating approval of this research<br>• Indicate the form of consent obtained (written/oral) or the reason that consent was not obtained (e.g. the data were analyzed anonymously)<br><br>**Animal Research (involving vertebrate animals, embryos or tissues)**<br>• Provide the name of the Institutional Animal Care and Use Committee (IACUC) or other relevant ethics board that reviewed the study protocol, and indicate whether they approved this research or granted a formal waiver of ethical approval<br>• Include an approval number if one was obtained<br>• If the study involved *non-human primates*, add *additional details* about animal welfare and steps taken to ameliorate suffering<br>• If anesthesia, euthanasia, or any kind of animal sacrifice is part of the study, include briefly which substances and/or methods were applied<br><br>**Field Research**<br><br>Include the following details if this study involves the collection of plant, animal, or other materials from a natural setting:<br>• Field permit number<br>• Name of the institution or relevant body that granted permission	
**Data Availability**<br><br>Authors are required to make all data underlying the findings described fully available, without restriction, and from the time of publication. PLOS allows rare exceptions to address legal and ethical concerns. See the PLOS Data Policy and FAQ for detailed information.	No - some restrictions will apply

A Data Availability Statement describing where the data can be found is required at submission. Your answers to this question constitute the Data Availability Statement and **will be published in the article**, if accepted.<br><br>**Important:** Stating 'data available on request from the author' is not sufficient. If your data are only available upon request, select 'No' for the first question and explain your exceptional situation in the text box.<br><br>Do the authors confirm that all data underlying the findings described in their manuscript are fully available without restriction?	
**Describe where the data may be found in full sentences. If you are copying our sample text, replace any instances of XXX with the appropriate details.**<br><br>• If the data are **held or will be held in a public repository**, include URLs, accession numbers or DOIs. If this information will only be available after acceptance, indicate this by ticking the box below. For example: *All XXX files are available from the XXX database (accession number(s) XXX, XXX.*).<br>• If the data are all contained **within the manuscript and/or Supporting Information files**, enter the following: *All relevant data are within the manuscript and its Supporting Information files.*<br>• If neither of these applies but you are able to provide **details of access elsewhere**, with or without limitations, please do so. For example:<br><br>*Data cannot be shared publicly because of [XXX]. Data are available from the XXX Institutional Data Access / Ethics Committee (contact via XXX) for researchers who meet the criteria for access to confidential data.*<br><br>*The data underlying the results presented in the study are available from (include the name of the third party*	The data used and/or analyzed during this study are available from the corresponding author on reasonable request.

and contact information or URL).<br>• This text is appropriate if the data are owned by a third party and authors do not have permission to share the data.<br><br>* typeset	
Additional data availability information:	

Powered by Editorial Manager® and ProduXion Manager® from Aries Systems Corporation

25 June, 2020

Dear Editor:

We would like to submit the enclosed manuscript entitled *Etiological and epidemiological characters of severe acute respiratory infection caused by multiple viruses and mycoplasma pneumoniae in adult patients in Jinshan of Shanghai, April 2017 to March 2018: a pilot hospital-based surveillance study,* which we wish to be considered for publication as a research article in PLOS ONE. No conflict of interest exists in the submission of this manuscript, and all the authors who made contributions to the manuscript are responsible for its content. I would like to declare on behalf of my co-authors that the work described with substantially the same content has not been submitted or published elsewhere.

Severe acute respiratory infection (SARI) is an important cause of morbidity and mortality in all ages worldwide, and is associated with various pathogens. So far, many studies regarding burden of SARI focuses on the children, epidemiological characterizations and distribution of the major etiologies are still limited on adult SARI patients in China. The present study is the first description of continuous surveillance of 19 respiratory pathogens of adult SARI patients in Shanghai, China. Our study implies that 63.0% of hospitalized adult SARI patients were identified as at least one positive pathogen infection, of whom 49.9% were single infection and 13.1% were multiple infections. The pathogens identified most frequently were *Mycoplasma pneumoniae* (23.93%), followed by adenovirus (11.59%) and influenza virus A/H3N2 (11.08%). The seasonality of pathogen-confirmed SARI patients conformed to a bimodal shape, with the first peak in summer and the second peak in winter.

We thought this study may be interesting to your readers. This manuscript has been reviewed and approved by all the authors for submission. We deeply appreciate your consideration of our manuscript, and we look forward to a favorable review.

Thank you and best regards.

Yours sincerely,

Jian Li

Clinical Research Center

Ruijin Hospital, Shanghai Jiao Tong University School of Medicine

Shanghai, China

Jian-Min Zhu

Department of Acute Infectious Diseases Control

Jinshan District Center for Diseases Control and Prevention

Shanghai, China

Manuscript Click here to access/download;Manuscript;Manuscript.docx

Etiological and epidemiological characters of severe acute respiratory infection caused by multiple viruses and *mycoplasma pneumoniae* in adult patients in Jinshan of Shanghai, April 2017 to March 2018: a pilot hospital-based surveillance study

Jian Li [1†],Can-Lei Song [2†],Tang Wang [2†], Yu-Long Ye [3], Jian-Ru Du [3], Shu-Hua Li [2], Jian-Min Zhu[2]

[†] Contributed equally

[1]*Clinical Research Center, Ruijin Hospital, Shanghai Jiao Tong University School of Medicine, Shanghai, China*

[2] *Department of Acute Infectious Diseases Control, Jinshan District Center for Diseases Control and Prevention, Shanghai, China*

[3]*Department of Microbiology, Jinshan District Center for Diseases Control and Prevention, Shanghai, China*

Email addresses:

Jian Li : nclijian@163.com

Can-Lei Song: 629309340@163.com

Tang Wang: 1090595528@qq.com

Yu-Long Ye: yeyulong99@163.com

Jian-Ru Du: 646947222@qq.com

Shu-Hua Li: hsli167@163.com

Jian-Min Zhu: zhujm12@163.com

Corresponding author:

Dr. Jian Li : Clinical Research Center, Ruijin Hospital, Shanghai Jiao Tong University School of

Medicine, 197 Ruijin Er Rd, Huangpu District, Shanghai, China,200025

Dr. Jian-Min Zhu: Department of Acute Infectious Diseases Control, Jinshan District Center for Diseases Control and Prevention, 94 Weisheng Rd, Jinshan District, Shanghai, China, 201599

Abstract

Background

Severe acute respiratory infection (SARI) presents a tremendous disease burden worldwide. Available research on active surveillance of hospitalized adult patients suffered from SARI in China was limited. This pilot~~ing~~ study aimed to identify etiologies and describe the demographic, epidemiological and clinical profiles of hospitalized SARI-associated patients over 16 years old in Jinshan, Shanghai.

Methods

Active surveillance was conducted at 1 sentinel hospital in Jinshan district, Shanghai from April 2017 to March 2018. Hospitalized SARI patients over 16 years old were enrolled, the pharyngeal swabs were collected within 24 hours of admission and tested for multiple respiratory viruses (including 18 common viruses) and *Mycoplasma pneumoniae* with real-time polymerase chain reaction. Demographic, epidemiological and clinical information were obtained from case report forms.

Results

Of 397 SARI patients enrolled, the median age was 68 years and 194(48.9%) were male**.** ~~Totally~~ 278(70.0%) had at least one underlying chronic medical conditions**.** The most frequent symptom was cough (99.2%) and sputum production (88.4%). The median duration of hospitalization was 10 days. A total of 250 patients (63.0%) were identified as at least one positive pathogen infection, of whom 198 (49.9%) were single infection and 52 (13.1%) were multiple infections. The pathogens identified most frequently were ~~*Mycoplasma*~~ *pneumoniae* (23.9%, 95/397), followed by adenovirus (11.6%, 46/397), influenza virus A/H3N2 (11.1%, 44/397) and human rhinovirus

(8.1%, 32/397). The seasonality of pathogen-confirmed SARI patients conformed to a bimodal shape, with the first peak in summer and the second peak in winter. The statistically significant differences were observed with respect to the proportions of dyspnea, radiographic diagnosis of pneumonia and presence of at least one comorbidity among patients who were single-infected by *M. pneumoniae*, AdV, HRhV, Flu A/H3N2, Flu A /pH1N1 and Flu B/Yamagata. No significant difference among different age groups was attained with regard to the positive rate of main pathogens.

Conclusions

~~*Mycoplasma*~~ *pneumoniae*, ~~adenovirus~~ and ~~influenza virus~~ A/H3N2 were the leading pathogens among hospitalized SARI patients aged more than 16 years in Jinshan district, Shanghai. Our finding highlights the importance of sustaining multi-pathogen surveillance of SARI patients in sentinel hospitals, which can provides useful information on etiology, epidemiology, and clinical characteristics.

Key words: Severe acute respiratory infection, sentinel surveillance, pathogen, epidemiology

Background

Severe acute respiratory infection (SARI) has been considered an important cause of morbidity and mortality in all ages worldwide, particularly in children, the elderly and individuals with compromised immune, cardiac and pulmonary systems [1-3]. It is estimated that SARI caused approximately 4.2 million of deaths annually. Of these, up to 90% are believed to occur in developing countries [4]. Various viral and bacterial pathogens are associated with SARI. Due to their extremely high potential to human-to-human transmission, these pathogens pose a substantial risk to human health. While bacterial infections exert critical influence on causing severe pneumonia [5], a significant proportion of SARI are attributed to viral infections such as influenza viruses A and B(Flu A/B), parainfluenza viruses(PIV), adenovirus(AdV), respiratory syncytial viruses(RSV)–, human coronaviruses(HCoV) and human rhinovirus(HRhV)[6]. Nevertheless, owing to the lack of gold standard methods to swiftly determine ~~etiological diagnoses~~, most of the patients may be treated with antibiotics empirically [7]. Rapid etiologic diagnosis therefore remains a significant public health challenge.

Routine pathogen monitoring is critical for preparedness and response to the epidemic of SARI. Since SARI is the leading cause of hospitalization in children under the age of 5 years and of febrile episodes in infants younger than 3 months old [8, 9], most studies regarding the burden of SARI test for viruses and focus on the children. A study of SARI surveillance in China revealed 90% of patients were aged <15 years [10]. Besides, majority of the data on the epidemiology of the etiologic agents of SARI come from more developed regions. Epidemiological characterizations and distribution of the major viral agents in adult SARI patients are still limited in developing regions. *Mycoplasma pneumoniae* (*M. pneumoniae*) has long been considered an important

etiology of respiratory disease, and is more frequently isolated among children and young adults [11, 12]. Limited research is available on active surveillance of hospitalized adult patients suffered from SARI in China, and the available data on viruses and *M. pneumoniae* is limited to adults in eastern China. In response, a piloting, active surveillance system for SARI had been initiated to conduct epidemiologic and etiologic monitoring of SARI caused by various viral pathogens and *M. pneumoniae* in adult inpatients in Jinshan district, Shanghai since April 2017. The aim of present study is to characterize the demography and epidemiology of SARI, to identify the etiologies and to assess the clinical profiles of SARI in hospitalized adult patients in Jinshan, Shanghai during 12 months of surveillance.

Materials and methods

Study setting

Jinshan district is a suburb and located in southwest Shanghai, P.R.China. Surveillance was piloted at Jinshan district central hospital since April 2017 and lasted for 12 months. This hospital was selected as it is one of the largest general hospitals in the district and also the national surveillance sentinel for influenza. It serves most of population of Jinshan district with a total of 636 beds. In 2017, the registered population in Jinshan district was 523641, of which 467320 (89.24%) were adults aged more than 18 years [13]. The annual mean temperature in this subtropical region is 14.8˚C and the average annual rainfall is about 1300mm.

Study subjects

All patients over 16 years old who were admitted to the sentinel hospital were screened by a trained physician between April 2017 and March 2018. Patients were defined as SARI case according to WHO definition if they have acute respiratory infection with measured fever of

≥38˚C, cough, with onset within the last 10 days and require hospitalization.

Data collection

After hospital admission, a standard case report form was completed for each eligible patient. The form comprised information on demographic characteristics (sex, age, weight, height, residence), vaccination (vaccinating influenza ~~vaccine during~~ 1 year before illness onset, ~~vaccinating~~ pneumococcal conjugate vaccine), admitting diagnosis, comorbidities (asthma, chronic bronchitis, chronic obstructive pulmonary disease(COPD), hypertension, diabetes, cardiovascular disease, tumor, ~~etc~~), clinical presentation (fever, cough, difficult breathing, sore throat, ~~etc~~), antibiotic treatments prior hospitalization, exposure history (smoking, visiting a live poultry market, contact with live poultry, contact with patient with fever and respiratory symptoms during 2 weeks before illness onset). At discharge, the form was updated to include information on treatment accepted in hospital, chest computed tomographic (CT) scans, complications and prognosis. Data were collected by the trained physician. To ensure the accuracy of data, spouses or caregivers who lived with the patients for more than 2 weeks before illness onset were interviewed and medical records of the patients were reviewed as well. Two radiologists interpreted chest CT scans independently. When disagreement arose, a third radiologist was consulted to reach a final decision. All the information that could identify the identification of patients was masked during or after data collection.

Specimen collection and laboratory testing

A single pharyngeal swab was collected from each SARI patient by nurse within 24 hours of admission following standard procedure. The swab was inserted into cryovial containing 3ml viral transport medium. The specimens were stored at 4 ˚C in the hospital and transferred to the

laboratory at Jinshan district center for disease control and prevention(CDC), where they were preserved at -70˚C until tests were performed. A total of 200 μl samples were adopted to extract viral RNA and DNA using the QIAamp Viral RNA Mini Kit (Qiagen, Hilden, Germany) following the manufacturer's instruction. Specimens were lysed at ~~strongly~~ denaturing conditions to deactivate RNases. ~~After adding alcohol and loading lysates onto the QIAamp spin column, viral RNA and DNA combined to the QIAamp silica membrane while contaminants passed through.~~ Pure viral RNA and DNA were eluted in low-salt buffer whereas impurities were removed. Total nucleic acid extracts were ~~further~~ ~~processed by~~ multiplex real-time reverse transcription polymerase chain reaction (RT-PCR). Respiratory pathogens 15 multiplex real-time RT-PCR diagnostic strategy was adopted to detect PIV (types 1, 2, 3 and 4), HCoV (types 229E, OC43, HKU1 and NL63), RSV (types A and B), HRhV, AdV, human metapneumovirus (HMPV), human bocavirus(HBoV) and *M. pneumoniae* using CFX96™ Real-time PCR System (Bio-Rad, Hercules, CA, USA) according to manufacturer's protocols. Besides, RNA from each specimen was identified for specific primers and probes that target Flu A/B using another real-time RT-PCR following the US CDC's protocol. Specimens found positive for Flu A and Flu B were subsequently sub typed for pandemic influenza A/H1N1(pH1N1) , seasonal A (H3N2) , together with Flu B/ Yamagata and Flu B/ Victoria, respectively [14]. These testing were performed in bio-safety level 2 laboratory of Jinshan CDC.

Statistics

The collected data were double-entered into a database constructed by EpiData 3.1. Logical checking for quality of data entry was conducted. The definition of single infection referred to an infection caused by one pathogen and multiple infections was defined as an infection caused by at

least 2 pathogens (virus/virus, virus/*M. pneumoniae*) in a single specimen. Categorical data were expressed as frequency and proportions, and continuous data were reported as median and inter-quartile range (IQR). Chi-squared test, Fisher's exact test and Mann-Whitney U test, as appropriate, were used to compare patients with and without confirmed pathogen in terms of demographic, clinical, epidemiologic characteristics, treatment and prognosis. For proportions, the binomial 95% confidence-interval was reported. The analysis was performed using SPSS v. 25.0 (IBM Corporation, Armonk, NY, USA) and all tests were performed two-sided at the 5% significance level.

Results

Demographic characteristics

From April 2017 to March 2018, a total of 397 patients meeting the SARI case definition were admitted to our sentinel site, of whom one or more positive pathogen were detected from 250 patients (63.0%; 95%CI: 58.2-67.7%). The median age of patients were 68 years (IQR: 59-78; range: 16 to 99 years) and 194(48.9%) were male. The majority of patients were elderly aged equal to or more than 60 years (295 cases) and accounting for 74.3% of total patients. Those ~~aged less~~ than 30 years represented only 6.3% of patients (25 cases). The percentage of body mass index (BMI) <20, between 20 and 25, >25 accounted for 29.7%, 52.4% and 17.9%, respectively. ~~Totally,~~ 278 SARI patients (70.0%) had at least one comorbidity (Table 1). The difference in proportion of gender, age, BMI and underlying chronic medical conditions between SARI patients with confirmed pathogen and those without confirmed pathogen did not show any statistical significance (*P*>0.05).

Etiology

Of 397 SARI patients, 198 (49.9%; 95% CI: 45.0-54.8%) were identified as single infection and 52 (13.1%; 95% CI: 9.8-16.4%) were multiple infections. The pathogen identified most frequently were *M. pneumoniae* in 95 (23.9% of the total samples) cases, followed by AdV in 46 (11.6%) cases, Flu A/H3N2 in 44 (11.1%) cases, HRhV in 32 (8.1%) cases, and Flu B/Yamagata in 25 (6.3%). Other viruses including HBoV, RSV, HMPV, HCoV, PIV, Flu B/Victoria and Flu A/pH1N1 were detected in a proportion ranging from 0.3% to 4.0% of infection (Table 2). The most frequently detected pathogens in patients with multiple infection were *M. pneumoniae* (84.6%, 44/52), AdV(28.8%, 15/52), HRhV(25.0%, 13/52), and Flu A/H3N2(17.3%, 9/52).

Clinical and epidemiologic characteristics

Pneumonia (222 cases, 55.9%) was the most common clinical diagnosis made by clinicians on admission and followed by bronchiolitis (68 cases, 17.1%). The most common symptom on admission was cough (99.2%) and sputum production (88.4%), followed by thoracalgia (7.1%) and pharyngalgia (6.8%). A temperature ≥ 39°C was recorded in 47.6% of SARI patients on admission. A total of 382 SARI patients (96.2%) had the chest CT performed, in which 258 (67.5%) were reported to have the presence of radiographic evidence of pneumonia. Thirty-two SARI patients and 30 patients had exposure of contacting with patients with fever and respiratory symptoms and contacting with live poultry during 2 weeks before their illness onset, respectively. Only 5 and 1 patient had history of vaccinating pneumococcal conjugate vaccine and influenza vaccine, respectively (Table 3). No significant differences in proportion of clinical and epidemiologic characteristics between SARI patients with confirmed pathogen and those without confirmed pathogen were attained except for chest radiographic examination. As for those single-infected patients by one of 6 kinds of main pathogens including *M. pneumoniae*, AdV,

HRhV, Flu A/H3N2, Flu A /pH1N1 and Flu B/Yamagata, the statistically significant differences were observed with respect to the proportions of dyspnea, radiographic diagnosis of pneumonia and presence of at least one comorbidity(Table 4). Notably, the presence of radiographic evidence of pneumonia was the highest in patients infected by *M. pneumonia*(74.5%) and dyspnea was the most common in patients with HRhV(21.1%).

Seasonal trends

Fig 1 showed the monthly variations in the number of SARI patient identified as *M. pneumoniae*, AdV, Flu A/H3N2, Flu A /pH1N1, HRhV, and Flu B/Yamagata infection. Over the 12-month period, the temporal distribution of pathogen-confirmed SARI patients had a bimodal shape, with the first peak in the summer and the second peak in the winter. The duration of first positive peak was 2 months from August to September, but the second peak only lasted for 1 month. Peaks of pathogen seemed to be more attributable to the number of *M. pneumoniae* and AdV detection. Besides, Flu A/H3N2 was responsible for summer peak, whereas Flu B/Yamagata and Flu A/pH1N1 were dominantly representative of winter peak. Unlike other pathogen, HRhV appeared to be detected all year along and did not show apparent seasonality. Distributions of seasonal pattern of positive rate of main 6 pathogens were shown in Fig. 2. The prevalence of Flu A/H3N2 peaked in summer (Jun-Aug) and autumn (Sep-Nov) with positive rate being 21.1% (20/95) and 22.3%(21/94), respectively (P<0.01). However, those of Flu A/pH1N1 and Flu B/Yamagata peaked dominantly in winter (Dec-Feb) with positive rate being 9.8% (13/132) and 18.9%(25/132), respectively (P<0.01). It's worth noting that no SARI patients linked to Flu B/Yamagata infection were detected in spring (Mar-May), summer and autumn. Significantly higher positive rate of *M. pneumoniae* was observed in autumn (43.6%, 41/94), as compared with other season (P<0.01).

HRhV in spring had a positive rate (18.4%,14/76) significantly higher than that of other season (*P*<0.01). The positive rate of AdV did not demonstrate obvious seasonality throughout the year (*P*>0.05).

Age distribution

The age group distributions of positive rate of main pathogens in SARI patients identified as *M. pneumoniae*, AdV, Flu A/H3N2, Flu A/pH1N1, HRhV, and Flu B/Yamagata infection were shown in Fig. 3. The prevalence of Flu A/pH1N1(8.0%) and AdV(20.0%) peaked in the group younger than 30 year old although the difference was not significant (*P*>0.05). The positive rate of *M. pneumonia*(36.2%) and Flu B/Yamagata(6.9%) were the highest in the group of 40-59 year old without statistical significance (*P*>0.05). Moreover, no significant difference between different age groups was observed with regard to the positive rate of Flu A/H3N2 and HRhV. Interestingly, no patients infected with Flu A/H3N2 and HRhV were detected in 30-39 year old group.

Treatment and prognosis

The median duration from illness onset to admission for SARI patients was 3 days (IQR: 2-5.5; range: 0 to 14 days), and median duration of hospitalization was 10 days (IQR: 8-13 days). Complications were present in 61 SARI patients, with electrolyte metabolism disorder (19 cases), respiratory failure (14 cases) and cardiac insufficiency (8 cases) being the most common as compared with other complications. Compared with SARI patients without confirmed pathogen, those with confirmed pathogen did not demonstrate more frequent use of antibiotics(levofloxacin, cephalosporin, azithromycin—), antivirals (oseltamivir), glucocorticoid and oxygen therapy (*P*>0.05). The duration of antibiotic use during hospitalization was 1-15 days (median: 9 days [IQR 5-11]) for SARI patients without confirmed pathogen and 1-20 days (median: 9 days [IQR

6-11]) for SARI patients with confirmed pathogen (*P*=0.68). ~~Totally 3~~ SARI patients died ~~in the periods of~~ hospitalization (Table 5).

Discussion

Hospital-based sentinel surveillance associated with SARI can provide a mechanism to monitor trends in this relatively severe disease and is critical for establishing platform to understand the epidemiologic and etiologic profiles at the local level. A monitoring study with regard to SARI patients in Georgia demonstrated that the proportion of patients positive for respiratory pathogens varied widely between seasons, from no positive for any of influenza in summer and early autumn (from July to October) to 30% of RSV in March in 2015–2017[1]. Another surveillance study of SARI patients in several countries revealed that the positive rate for influenza fluctuated extensively depending on country and season from 2.1% in Armenia in 2011–2012 to 100% in Albania in 2009–2010 [15]. A comparative study of viral profile in hospitalized SARI children in Beijing and Shanghai, China showed different patterns of viral profiles in 2 cities, in which RSV(52.9%) and HRhV/enterovirus(34.7%) were the most prevalent etiological agents of SARI in Beijing, whereas HRhV/enterovirus(33.6%) and HBoV(17.7%) were the main pathogens of SARI in Shanghai[16]. The early detection of divergent SARI pathogens through the sentinel surveillance network can measure the burden of disease severity and better prepare a region for emergency response. To our knowledge, this pilot study is the first description of continuous surveillance covering 19 respiratory pathogens among adult SARI patients in Shanghai of eastern China, which has provided better understanding of the epidemiology, etiologic spectrum and clinical profile. ~~Within~~ 1 year of active surveillance, 397 patients ~~meeting~~ the established case definition of SARI were eligible for enrolling in this study, and 63.0% of patients were tested

positive for at least one pathogen. Our finding reached consensus with those reported elsewhere which revealed etiologies ranging from 50% to 85% of hospitalized SARI cases [7, 17-18].

During the pilot phase from April 2017 to March 2018 in Jinshan district, the main etiologies of SARI varied seasonally and *M. pneumoniae*, AdV, Flu A/H3N2, HRhV, together with Flu B/Yamagata were predominant pathogen depending on months, although other viruses such as Flu A/ pH1N1, HBoV, RSV, HMPV, HCoV, PIV and Flu B/Victoria were also present. Since our sentinel aims at surveillance of adult SARI patients, most of the enrolled patients were elder persons between 60-79 years old (52.1%) and the second most in those aged 80 and above (22.2%) . This was different from that observed in Madagascar [7], where the most suffered was observed in the less than 5-year-old age group (81.1%). Another similar study in Egypt also showed that children <5 years old accounted for 35.3% of SARI cases, whereas the elderly more than 64 years old only made up 25.2%[2]. Our study demonstrates that, unlike the tropical region, elder individuals are the most vulnerable group for suffering from SARI in subtropical region. In the present study, at least one chronic medical condition occurred in 70% of SARI patients. Our study population presented a high prevalence of comorbidities compared with the study in Hubei province, China [10], and may be partially explained by the inconsistence of socio-economic development between 2 regions. Hypertension and cardiovascular disease was observed in 38.3% and 7.6% of our population, respectively. And patients with confirmed pathogen had higher prevalence of cardiovascular disease than those without confirmed pathogen. One study suggested that diagnosed cardiovascular disease was commonly related to fatal endpoints among influenza positive SARI cases [19]. Our study revealed that the proportion of vaccinating influenza vaccine and pneumococcal conjugate vaccine was quite low, so the respiratory disease vaccination

programs with the target of individuals with cardiovascular related disease should be recommended. In this study, most patients presented with cough, sputum production and fever. These clinical features bear some resemblance to the report in previous study [1]. It should be alerted that empirical use of antibiotics use during hospitalization occurred in 99% patients in present study due to unavailability of rapid determining etiological diagnoses.

M. pneumoniae (23.9%) was the most ~~frequent~~ pathogen in present study. The positive detection rate of *M. pneumoniae* echoed the published data (19.7%) in north China [20]. A prospective study conducted in Hong Kong among adults hospitalized with pneumonia in 2004 to 2005 found that *M. pneumoniae* was detected in 78/1,193 patients (6.5%)[21]. *M. pneumoniae* occurs endemically worldwide in many different climates. *M. pneumoniae* was mostly detected in autumn (43.6%) and spring (27.6%) in our study, but *M. pneumoniae* in Istanbul was more commonly identified in summer (44.9%) and winter (22.4 %) [22]. As the second common pathogen in this study, the positive rate of AdV did not significantly differ along with season change, and this trend in seasonality was consistent with previous reported seasonality of AdV detecting rate in China [23]. In contrast with the seasonality of viral respiratory SARI observed in Georgia in 2015-2017 and in northern China in 2014-2016, where the distinct winter-only peak of influenza circulation was observed[24,25], we found influenza peaked both in winter and in summer. Overall, influenza virus was common in this study, with Flu A/H3N2 dominating in summer, and Flu B/Yamagata and Flu A/pH1N1 dominating in winter. Our finding suggested that SARI illness caused by Flu B virus were nearly twice the illness associated with Flu A virus infections in winter, which was different from one study in USA in which estimated excess hospitalization rates associated with Flu B were lower than for Flu A/H3N2 [26]. In this

study, we also noted that no statistical difference were found in the positive rate of pathogens identified as *M. pneumoniae*, AdV, Flu A/H3N2, Flu A /pH1N1, HRhV, and Flu B /Yamagata among different age group. This phenomenon was basically the same as the previous study in north China [23], and may be attributable to susceptibility to these common viruses in different age group of adults. As reported elsewhere [27], co-infections were found relatively common in present study. ~~Totally~~ 13.1% of SARI patients were reported to have more than one pathogen infection, and the percentage was consistent with the finding of previous study (11.7%) [17].

Limitations

Our study was subject to several limitations. First, as a pilot project, this study was ~~enforced~~ at only 1 hospital although this hospital is the biggest hospital in the ~~site~~, so the finding may have relatively limited generalizability. Actually, prevalence of each pathogen may differ for regions having different climatic, demographic patterns and access to healthcare. Second, the result was based on SARI surveillance of a 12-month period, and the burden derived from SARI may not reflect the situation over several years. Finally, the pathogens which were included in this piloting surveillance study did not involve in related respiratory bacterium such as pneumococcus and ~~Bordetella pertussis~~ owing to limited financial support. Indeed, the inclusion of bacterium surveillance is under consideration to ~~incorporate~~ into our program.

Conclusions

In conclusion, the current study is the first study ~~surveilling~~ hospitalized adult SARI patients for most respiratory viruses and *M. pneumoniae* in Shanghai, and confirms that multiple respiratory pathogens may circulate among the SARI population and vary with the climatic and demographic characteristics. The finding highlights the importance of sustaining ~~enhancing of~~ sentinel

surveillance of SARI patients at the local and national levels, which can contribute to accurately evaluate the prevalence of etiology associated to SARI and the burden of disease, and, more importantly, to shape public policy on SARI prevention and response to SARI activity.

Abbreviations

AdV: Adenovirus; BMI: Body mass index; CDC: Center for disease control and prevention; COPD: Chronic obstructive pulmonary disease; Flu: Influenza; HBoV: Human bocavirus; HCoV: Human coronaviruses; HMPV:human metapneumovirus; HRhV: Human rhinovirus; IQR: Inter-quartile range; *M. pneumonia*: Mycoplasma pneumonia; PIV: Parainfluenza virus; RSV: Respiratory syncytial virus; RT-PCR: Reverse transcription polymerase chain reaction; SARI: Severe acute respiratory infection

Declarations

Acknowledgements

We thank physicians and nurses from Jinshan district central hospital for their significant contribution to the data collection and specimen collection.

Funding

This work was supported by the Research Project of Shanghai Municipal Health Commission (201940428) and the Infectious Disease and Epidemiology Project of the 6th Jinshan District Medical Key Specialty Construction (JSZK2019B05). The funder had no role in study design, data collection and analysis, decision to publish or preparation of manuscript.

Availability of data and materials

The datasets used and/or analyzed during this study are available from the corresponding author on reasonable request.

Authors' contributions

JL and JMZ conceived and designed the study and critically revised the manuscript. YLY and JRD performed the experiments. JL, CLS and TW analyzed the data and drafted the manuscript. CLS, TW and SHL participated in the study investigation, data entry and clean. All the authors have read and approved the final manuscript.

Ethics approval and consent to participate

This study belonged to the part of hospital-based surveillance program of SARI of Shanghai, and approved by the ethical review committee of the Shanghai Municipal Center for Disease Control and Prevention. Written informed consent was obtained from patients or proxies before enrollment. Data were accessible only to authorized members of the core study team.

Consent for publication

Not applicable.

Competing interests

The authors declare that they have no competing interests.

References

[1]Chakhunashvili G, Wagner AL, Power LE, Janusz CB, Machablishvili A, Karseladze I, et al. Severe Acute Respiratory Infection (SARI) sentinel surveillance in the country of Georgia, 2015-2017. PLos One.2018;13(7):e0201497.

[2]Elhakim MM, Kandil SK, Abd Elaziz KM, Anwar WA. Epidemiology of Severe Acute Respiratory Infection (SARI) cases at a sentinel site in Egype,2013-15. J Public Health (Oxf). 2019.doi: 10.1093/pubmed/fdz053.

[3]Williams B, Gouws E, Boschi-Pinto C, Bryce J, Dye C. Estimates of world-wide distribution of child deaths from acute respiratory infections. Lancet Infect Dis. 2002;2:25–32.

[4]WHO. The global burden of disease: 2004 update. Geneva: World Health Organization. 2008. http://www.who.int/healthinfo/global_burden_disease/GBD_report_2004update_full.pdf.

[5] Watt JP, Wolfson LJ, O'Brien KL, Henkle E, Deloria-Knoll M, McCall N,et al. Burden of disease caused by Haemophilus influenzae type b in children younger than 5 years: global estimates. Lancet. 2009;374(9693): 903–911.

[6]Subramoney K, Hellferscee O, Pretorius M, Tempia S, McMorrow M, von Gottberg A, et al. Human bocavirus, coronavirus,and polyomavirus detected among patients hospitalised with severe acute respiratory illness in South Africa, 2012 to 2013. Health Sci Rep. 2018;1(8):e59.

[7]Razanajatovo NH, Guillebaud J, Harimanana A, Rajatonirina S, Ratsima EH, Andrianirina ZZ, et al. Epidemiology of severe acute respiratory infections from hospital-based surveillance in Madagascar, November 2010 to July 2013. PLos One.2018;13(11):e0205124.

[8]World Health Organization. WHO global technical consultation: global standards and tools for influenza surveillance. Geneva: World Health Organization; 2011.

[9] Byington CL, Enriquez FR, Hoff C, et al. Serious bacterial infections in febrile infants 1 to 90 days old with and without viral infections. Pediatrics. 2004;113(6):1662–6.

[10]Yu H, Huang J, Huai Y, Guan X, Klena J, Liu S, et al. The substantial hospitalization burden of influenza in central China: surveillance for severe, acute respiratory infection, and influenza viruses, 2010-2012.Influenza Other Respir Viruses. 2014;8(1):53-65.

[11] Foy HM, Cooney MK, McMahan R, et al. Viral and mycoplasmal pneumonia in a prepaid medical care group during an eight-year period. Am J Epidemiol. 1973; 97: 93-102.

[12]Waites KB, Xiao L, Liu Y, et al. Mycoplasma pneumoniae from the respiratory tract and beyond. Clin Microbiol Rev.2017; 30: 747-809.

[13] Bureau of statistics of Jinshan district. Jinshan statistical yearbook 2018. http://www.jinshan.gov.cn/Attach/Attaches/201911/20191114051723 7545.pdf.

[14] WHO. Manual for the laboratory diagnosis and virological surveillance of influenza. 2011. http://whqlibdoc.who.int/publications/2011/9789241548090_eng.pdf.

[15] Meerhoff TJ, Simaku A, Ulqinaku D, Torosyan L, Gribkova N, Shimanovich V, et al. Surveillance for severe acute respiratory infections (SARI) in hospitals in the WHO European region—an exploratory analysis of risk factors for a severe outcome in influenza-positive SARI cases. BMC Infect Dis. 2015;15: 1.

[16]Zhao Y, Lu R, Shen J, Xie Z, Liu G, Tan W. Comparison of viral and epidemiological profiles of hospitalized children with severe acute respiratory infection in Beijing and Shanghai, China. BMC Infect Dis. 2019;19(1):729.

[17]Huo X, Qin Y, Qi X, Zu R, Tang F, Li L, et al. Surveillance of 16 respiratory viruses in patients with influenza-like illness in Nanjing, China. J Med Virol. 2012;84(12):1980-4.

[18]Breiman RF, Cosmas L, Njenga M, Williamson J, Mott JA, Katz MA, et al. Severe acute respiratory infection in children in a densely populated urban slum in Kenya, 2007–2011. BMC Infect Dis. 2015; 15: 95.

[19]Butsashvili M, Kandelaki G, Zakhashvili K, Machablishvili A, Avaliani N. Influenza-associated mortality in georgia (2009-2011). Influenza Res Treat. 2012;2012:480763.

[20]Xu W, Guo L, Dong X, Li X, Zhou P, Ni Q, et al. Detection of viruses and mycoplasma pneumonia in hospitalized patients with severe acute respiratory infection in northern

China,2015-2016. Jpn J Infec Dis. 2018;71(2):134-139.

[21] Lui G, Ip M, Lee N, Rainer TH, Man SY, Cockram CS, et al. Role of 'atypical pathogens' among adult hospitalized patients with community-acquired pneumonia. Respirology.2009; 14:1098–1105.

[22] Goktas S, Sirin MC. Prevalence and seasonal distribution of respiratory viruses during the 2014 – 2015 season in Istanbul. Jundishapur J Microbiol. 2016; 9(9): e39132.

[23]Zhao Y, Lu R, Shen J, Xie Z, Liu G, Tan W. Comparison of viral and epidemiological profiles of hospitalized children with severe acute respiratory infection in Beijing and Shanghai, China. BMC Infect Dis.2019;19(1):729.

[24]Azziz Baumgartner E, Dao CN, Nasreen S, Bhuiyan MU, Mah-E-Muneer S, Ai Mamun A. et al. Seasonality, timing, and climate drivers of influenza activity worldwide. J Infect Dis. 2012; 206(6):838-46.

[25]Zhang Y, Muscatello DJ, Wang Q, Yang P, Pan Y, Huo D, et al. Hospitalizations for influenza-associated severe acute respiratory infection, Beijing, China, 2014-2016. Emerg Infect Dis. 2018;24(11):2098-2102.

[26] Thompson WW, Shay DK, Weintraub E, Brammer L, Bridges CB, Cox NJ, et al. Influenza-associated hospitalizations in the United States. JAMA. 2004; 292(11):1333–1340.

[27]Hasman H, Pachucki CT, Unal A, Nguyen D, Devlin T, Peeples ME, et al. Aetiology of influenza-like illness in adults includes parainfluenzavirus type 4. J Med Microbiol. 2009;58(Pt4):408–413.

Table 1. Demographic characteristics of adult SARI patients in a surveillance hospital in Jinshan, Shanghai, April 2017 to March 2018

Characteristics	All SARI patients (%) [n=397]	SARI patients with confirmed pathogen (%) [n=250]	SARI patients without confirmed pathogen (%) [n=147]	*P* value*
Male gender	194(48.9)	127(50.8)	67(45.6)	0.315
Age group(median, years)	68.0	67.0	69.0	0.357
<30	25(6.3)	17(6.8)	8(5.4)	0.786
30-39	19(4.8)	10(4.0)	9(6.1)	
40-59	58(14.6)	39(15.6)	19(12.9)	
60-79	207(52.1)	128(51.2)	79(53.7)	
≥80	88(22.2)	56(22.4)	32(21.9)	
BMI				0.657
<20	118(29.7)	73(29.2)	45(30.6)	
20-25	208(52.4)	135(54.0)	73(49/7)	
>25	71(17.9)	42(16.8)	29(19.7)	
Chronic medical conditions				
At least one	278(70.0)	178(71.2)	100(68.0)	0.505
Asthma	12(3.0)	6(2.4)	6(4.1)	0.345
Chronic bronchitis	49(12.3)	30(12.0)	19(12.9)	0.787
COPD	28(7.1)	13(5.2)	15(10.2)	0.060
Hypertension	152(38.3)	95(38.0)	57(38.8)	0.878
Cardiovascular disease	30(7.6)	22(8.8)	8(5.4)	0.222
Diabetes	61(15.4)	38(15.2)	23(15.6)	0.905
Cerebrovascular disorder	20(5.0)	14(5.6)	6(4.1)	0.504
Tumor	19(4.8)	14(5.6)	5(3.4)	0.322

*The *P* values denoted comparisons between SARI patients with confirmed pathogen and SARI patients without confirmed pathogen.

Table 2. Etiology distribution of adult SARI patients in a surveillance hospital in Jinshan, Shanghai, April 2017 to March 2018

Viral etiology	Frequency(n)	Percent of patients (%)
Influenza virus A		
pH1N1	16	4.0
H3N2	44	11.1
Influenza virus B		
Yamagata	25	6.3
Victoria	2	0.5
Parainfluenza virus		
Type 1	8	2.0
Type 2	0	0
Type 3	6	1.5
Type 4	5	1.3
Human coronavirus		
Type 229E	6	1.5
Type OC43	4	1.0
Type HKU1	6	1.5
Type NL63	8	2.0
Respiratory syncytial virus		
Type A	0	0
Type B	2	0.5
Human rhinovirus	32	8.1
Adenovirus	46	11.6
Human metapneumovirus	6	1.5
Human bocavirus	1	0.3
Mycoplasma pneumonia	95	23.9
Single infection	198	49.9
Multiple infection		

2 pathogens	43	10.8
3 pathogens	8	2.0
4 pathogens	1	0.3

Table 3. Clinical and epidemiologic characteristics of adult SARI patients in a surveillance hospital in Jinshan, Shanghai, April 2017 to March 2018

Characteristics	All SARI patients (%) [n=397]	SARI patients with confirmed pathogen (%) [n=250]	SARI patients without confirmed pathogen (%) [n=147]	*P* value*
Temperature ≥39˚C	189(47.6)	126(50.4)	63(42.9)	0.176
Cough	394(99.2)	249(99.6)	145(98.6)	0.558
Sputum production	351(88.4)	219(87.6)	132(89.8)	0.509
Pharyngalgia	27(6.8)	18(7.2)	9(6.1)	0.680
Thoracalgia	28(7.1)	19(7.6)	9(6.1)	0.687
Dyspnea	19(4.8)	11(4.4)	8(5.4)	0.808
Runny nose	11(2.8)	7(2.8)	4(2.7)	1.000
Vomiting	15(3.8)	10(4.0)	5(3.4)	0.795
Chest radiographic exam	382(96.2)	236(94.4)	146(99.3)	0.013
Presence of radiographic diagnosis of pneumonia	258/382(67.5)	153/236(64.8)	105/146(71.9)	0.349
Visiting a live poultry market	3(0.8)	3(1.2)	0(0)	0.299
Contact with live poultry	30(7.6)	19(7.6)	11(7.5)	1.000
Contact with patient with fever	32(8.1)	24(9.6)	8(5.4)	0.182
Smoking				0.860
Current s	43(10.8)	28(11.2)	15(10.2)	
Former	66(16.6)	43(17.2)	23(15.6)	
Never	288(72.6)	179(71.6)	109(74.2)	
Vaccinating pneumococcal conjugate vaccine	5(1.3)	3(1.2)	2(1.4)	1.000
Vaccinating influenza vaccine	1(0.3)	1(0.4)	0(0)	1.000

*The *P* values denoted comparisons between SARI patients with confirmed pathogen and SARI patients without confirmed pathogen.

Table 4. Characteristics of single-infected SARI patients in a surveillance hospital in Jinshan, Shanghai, April 2017 to March 2018 by six main pathogens

	M. pneumoniae (%) [n=51]	AdV(%) [n=31]	HRhV(%) [n=19]	Flu A/H3N2(%) [n=35]	Flu B/Yamagata(%) [n=21]	Flu A /pH1N1(%) [n=16]	*P* value*
Male	28(54.9)	19(61.3)	8(42.1)	18(51.4)	9(42.9)	8(50.0)	0.750
Age group(years)							0.247
<30	5(9.8)	3(9.7)	1(5.3)	1(2.9)	0(0)	2(12.5)	
30-39	3(5.9)	3(9.7)	0(0)	0(0)	1(4.8)	1(6.3)	
40-59	12(23.5)	1(3.2)	2(10.5)	5(14.3)	3(14.3)	2(12.5)	
60-79	20(39.2)	15(48.4)	8(42.1)	24(68.6)	12(57.1)	9(56.3)	
≥80	11(21.6)	9(29.0)	8(42.1)	5(14.3)	5(23.8)	2(12.5)	
at least one comorbidity	25(49.0)	23(74.2)	13(68.4)	26(74.3)	18(85.7)	11(68.8)	0.034
Temperature ≥39˚C	30(58.8)	16(51.6)	6(31.6)	16(45.7)	9(42.9)	8(50.0)	0.444
Cough	51(100)	31(100)	19(100)	34(97.1)	21(100)	16(100)	0.705
Sputum production	39(76.5)	29(93.5)	15(78.9)	30(85.7)	19(90.5)	16(100)	0.120
Pharyngalgia	3(5.9)	3(9.7)	2(10.5)	2(5.7)	2(9.5)	2(12.5)	0.876
Thoracalgia	4(7.8)	2(6.5)	1(5.3)	0(0)	2(9.5)	1(6.3)	0.523
Dyspnea	0(0)	1(3.2)	4(21.1)	1(2.9)	1(4.8)	0(0)	0.007
Runny nose	1(2.0)	1(3.2)	1(5.3)	1(2.9)	0(0)	2(12.5)	0.360
Vomiting	0(0)	3(9.7)	0(0)	3(8.6)	1(4.8)	1(6.3)	0.123
Presence of radiographic diagnosis of pneumonia	38(74.5)	17(54.8)	13(68.4)	15(42.9)	13(61.9)	7(43.8)	0.042
Contact with live poultry	6(11.8)	3(9.7)	2(10.5)	1(2.9)	1(4.8)	1(6.3)	0.753
Contact with patient with fever	3(5.9)	4(12.9)	2(10.5)	1(2.9)	3(14.3)	2(12.5)	0.442
Current Smoking	2(3.9)	4(12.9)	2(10.5)	6(17.1)	3(14.3)	3(18.8)	0.333
Former Smoking	10(19.6)	7(22.6)	2(10.5)	7(20.0)	3(14.3)	0(0)	
Never Smoking	39(76.5)	20(64.5)	15(78.9)	22(62.9)	15(71.4)	13(81.3)	

*The *P* values denoted comparisons among six main pathogens.

Table 5. Treatment and prognosis of adult SARI patients in a surveillance hospital in Jinshan, Shanghai, April 2017 to March 2018

Characteristics	All SARI patients (%) [n=397]	SARI patients with confirmed pathogen (%) [n=250]	SARI patients without confirmed pathogen (%) [n=147]	*P* value*
Clinical course(median, days)				
From illness onset to admission	3	3	3	0.567
Length of hospitalization	10	10	10	0.545
Antibiotics prior hospitalization	241（61.0）	151（60.9）	90（61.2）	0.723
Antibiotics during hospitalization	393(99.0)	246(98.4)	147(100)	0.301
Antiviral	11(2.8)	7(2.8)	4(2.7)	1.000
Glucocorticoids	112(28.2)	72(27.2)	40(28.8)	0.734
Oxygen therapy	196(49.4)	124(49.6)	72(49.0)	0.918
Complications	61(15.4)	37(14.8)	24(16.3)	0.684
Death	3(0.8)	2(0.8)	1(0.7)	1.000

*The *P* values denoted comparisons between SARI patients with confirmed pathogen and SARI patients without confirmed pathogen.

Figure Click here to access/download;Figure;Figure.docx

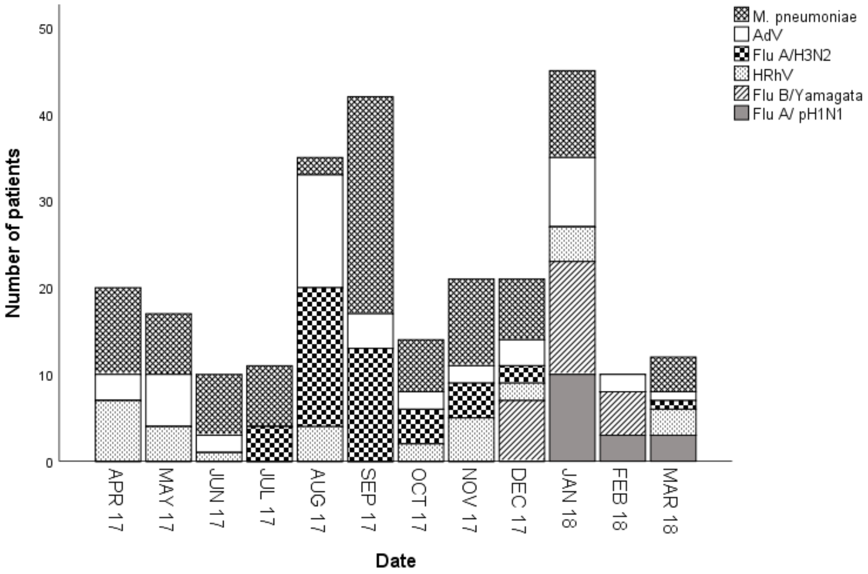

Fig. 1 Monthly variation of six main pathogens detected among adult SARI patients in a surveillance hospital in Jinshan, Shanghai, April 2017 to March 2018

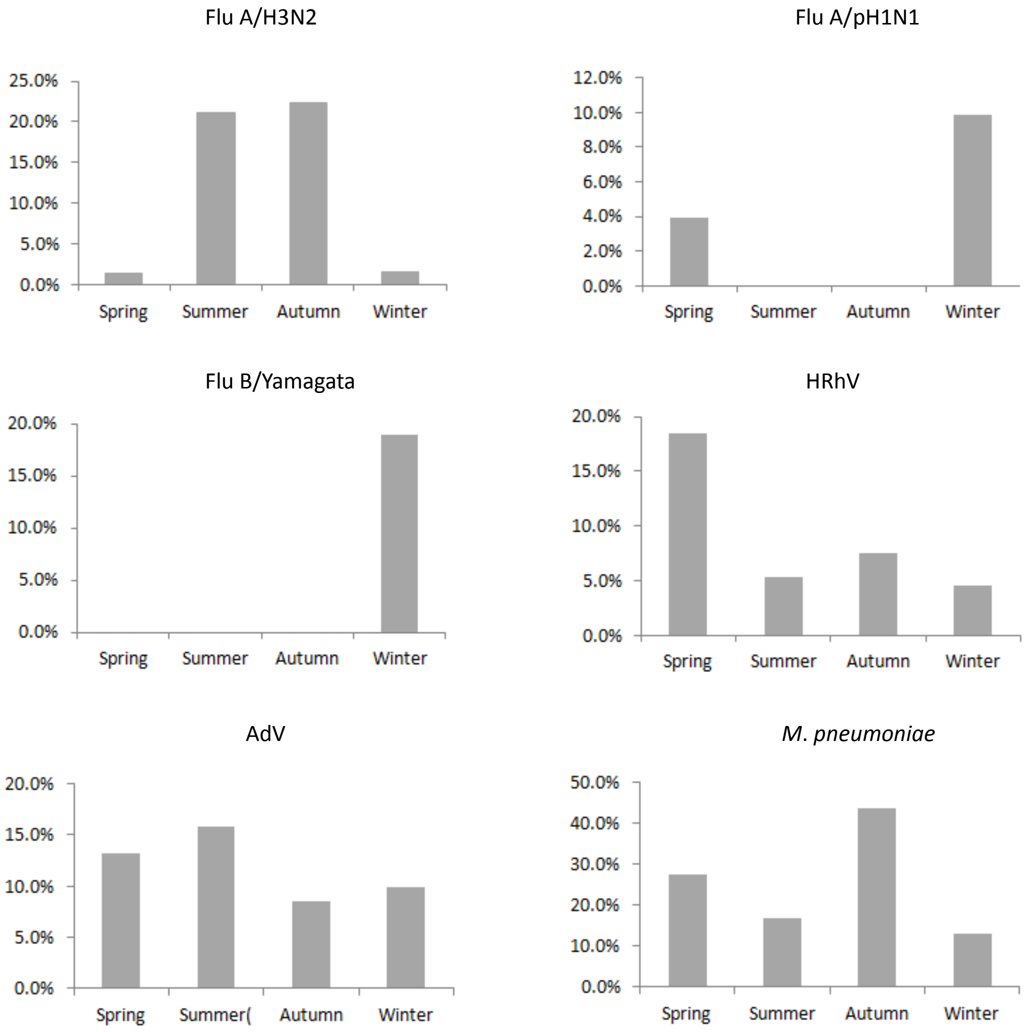

Fig. 2 Detection rate of six main pathogens among adult SARI patients in different seasons in a surveillance hospital in Jinshan, Shanghai.

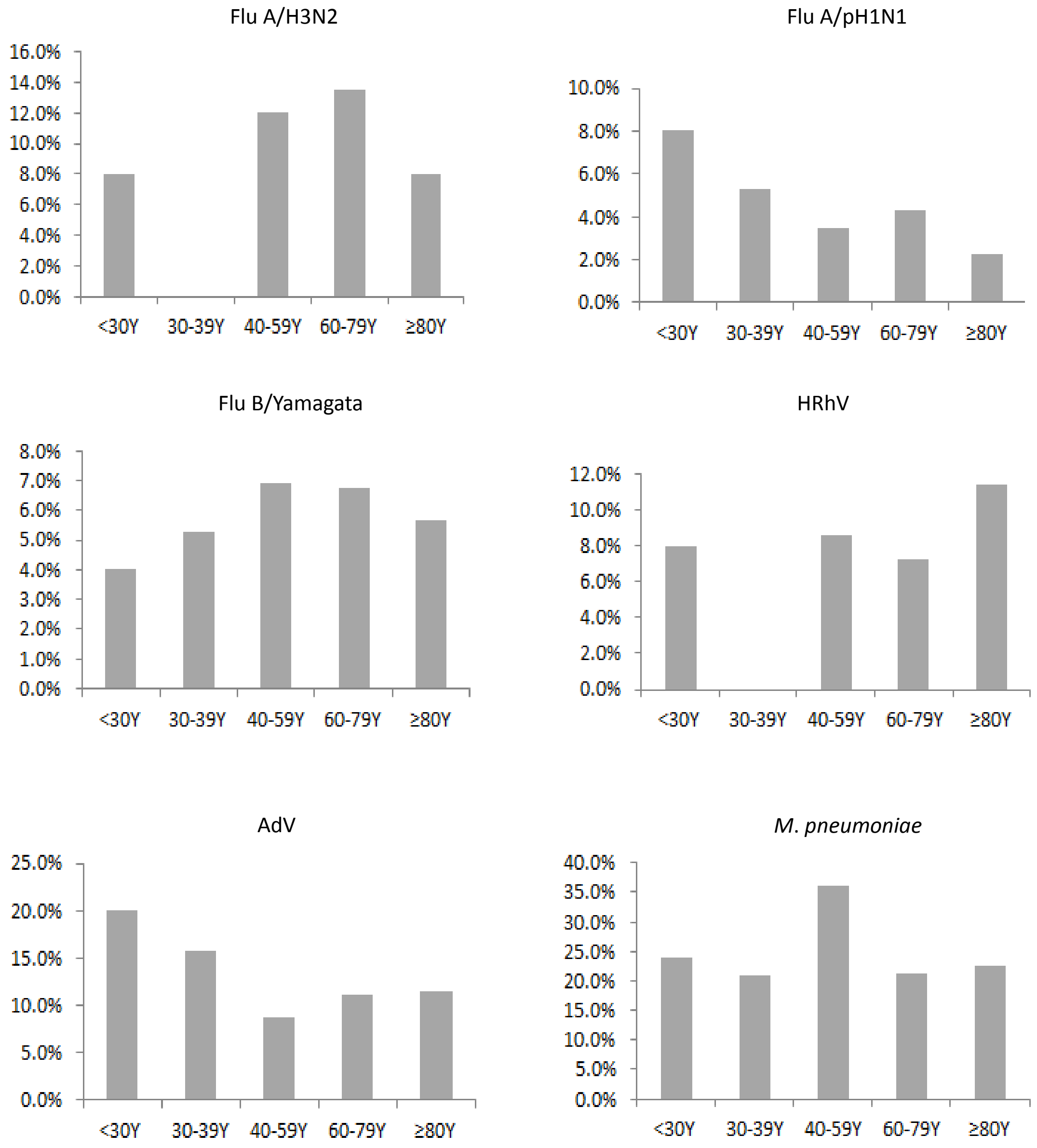

Fig. 3 Detection rate of six main pathogens among SARI patients according to age groups in a surveillance hospital in Jinshan, Shanghai, April 2017 to March 2018

Click here to access/download
Supporting Information
STROBE_checklist_v4_combined_PlosMedicine.docx

Click here to access/download
Other
PLOSOne_Clinical_Studies_Checklist.docx

Click here to access/download
Other
Shanghai CDC IRB Approval English Version.docx

Click here to access/download
Other
Shanghai CDC IRB Approval CHN.pdf
