# Supplementary material for: Etiological and epidemiological characteristics of severe acute respiratory infection caused by multiple viruses and Mycoplasma pneumoniae in adult patients in Jinshan, Shanghai: A pilot hospital-based surveillance study
Source: PLoS One. 2021 Mar 22;16(3):e0248750. doi: 10.1371/journal.pone.0248750 (PMC7984646; doi:10.1371/journal.pone.0248750)
Supplement: Attachment — Submitted filename: PONE-D-20-19561_R1_reviewer-comments.pdf [file pone.0248750.s006.pdf]

PLOS ONE

Etiological and Epidemiological Characteristics of Severe Acute Respiratory Infection Caused by Multiple Viruses and Mycoplasma Pneumoniae in Adult Patients in Jinshan, Shanghai: A Pilot Hospital-based Surveillance Study

--Manuscript Draft--

**Manuscript Number:**	PONE-D-20-19561R1
**Article Type:**	Research Article
**Full Title:**	Etiological and Epidemiological Characteristics of Severe Acute Respiratory Infection Caused by Multiple Viruses and Mycoplasma Pneumoniae in Adult Patients in Jinshan, Shanghai: A Pilot Hospital-based Surveillance Study
**Short Title:**	Etiological and epidemiological characteristics of severe acute respiratory infection
**Corresponding Author:**	JIAN LI<br>Ruijin Hospital, Shanghai Jiao Tong University, School of Medicine<br>Shanghai, CHINA
**Keywords:**	Severe acute respiratory infection, sentinel surveillance, pathogen, epidemiology
**Abstract:**	Background<br>Severe acute respiratory infection (SARI) results in a tremendous disease burden worldwide. Available research on active surveillance among hospitalized adult patients suffering from SARI in China is limited. This pilot study aimed to identify associated etiologies and describe the demographic, epidemiological and clinical profiles of hospitalized SARI patients aged over 16 years in Jinshan, Shanghai.<br>Methods<br>Active surveillance was conducted at 1 sentinel hospital in Jinshan district, Shanghai, from April 2017 to March 2018. Hospitalized SARI patients aged more than 16 years were enrolled, and nasopharyngeal swabs were collected within 24 hours of admission and tested for multiple respiratory viruses (including 18 common viruses) and Mycoplasma pneumoniae (M. pneumoniae) with real-time polymerase chain reaction. Demographic, epidemiological and clinical information was obtained from case report forms.<br>Results<br>In total, 397 SARI patients were enrolled; the median age was 68 years, and 194 (48.9%) patients were male . A total of 278 (70.0%) patients had at least one underlying chronic medical condition . The most frequent symptoms were cough (99.2%) and sputum production (88.4%). The median duration of hospitalization was 10 days. A total of 250 infection patients (63.0%) were positive for at least one pathogen, of whom 198 (49.9%) were positive for a single pathogen and 52 (13.1%) were positive for multiple pathogens. The pathogens identified most frequently were M. pneumoniae (23.9%, 95/397), followed by adenovirus (AdV) (11.6%, 46/397), influenza virus A/H3N2 (Flu A/H3N2) (11.1%, 44/397), human rhinovirus (HRhV) (8.1%, 32/397), influenza virus B/Yamagata (Flu B/Yamagata) (6.3%, 25/397), pandemic influenza virus A/H1N1 (Flu A/pH1N1) (4.0%, 16/397), parainfluenza virus (PIV) type 1 (2.0%, 8/397), human coronavirus (HCoV) type NL63 (2.0%, 8/397), HCoV type 229E (1.5%, 6/397), HCoV type HKU1 (1.5%, 6/397), PIV type 3 (1.5%, 6/397), human metapneumovirus (HMPV) (1.5%, 6/397), PIV type 4 (1.3%, 5/397), HCoV type OC43 (1.0%, 4/397), influenza virus B/Victoria (Flu B/Victoria) (0.5%, 2/397), respiratory syncytial virus (RSV) type B (0.5%, 2/397), and human bocavirus (HBoV) (0.3%, 1/397). The seasonality of pathogen-confirmed SARI patients had a bimodal distribution, with the first peak in summer and the second peak in winter. Statistically significant differences were observed with respect to the rates of dyspnea, radiographically diagnosed pneumonia and the presence of at least one comorbidity in patients who were infected with only M . pneumoniae , AdV, HRhV, Flu A/H3N2, Flu A /pH1N1 or Flu B/Yamagata. The differences in the positivity rates of the above 6 pathogens among the different age groups were nonsignificant.<br>Conclusions<br>M . pneumoniae , AdV and Flu A/H3N2 were the main pathogens detected in hospitalized SARI patients aged more than 16 years in Jinshan district, Shanghai. Our findings highlight the importance of sustained multipathogen surveillance among SARI patients in sentinel hospitals, which can provide useful information on SARI etiologies,

Powered by Editorial Manager® and ProduXion Manager® from Aries Systems Corporation

	epidemiology, and clinical characteristics.
**Order of Authors:**	JIAN LI
	Can-Lei Song
	Tang Wang
	Yu-Long Ye
	Jian-Ru Du
	Shu-Hua Li
	Jian-Min Zhu
**Response to Reviewers:**	Response to Specific Comments:<br>1.Specimen collection and laboratory testing: This section need further clarification. Please specify the multiplex PCR used. Did the authors used method described in previous literatures or commercial kit?<br>Answer: We thank for these suggestions and have made further clarification. The multiplex PCR used is the commercial kit. We added the data about multiplex PCR and made further clarification (see Page 7, line 154 to Page 8, line 156 in Revised Manuscript with Track Changes, the same below).<br>Line 219 – 223: Not sure what the authors wish to convey, please rephrase for clarification.<br>Answer: We are sorry and have rephrased these sentences (see Page 11, line232-236).<br>2.Line 260 – 265: Not clear on what the authors' intention on these statement, please clarify.<br>Answer: These sentences in line 260-265 mean to show that there were no significant differences of therapy between SARI patients with confirmed pathogen and those without confirmed pathogen. We have modified our text as advised (see Page 13, line 280-286).<br>3.Line 295 – 299: This argument does not hold. These are not fair comparison since this study excluded children.<br>Answer: This comment is appreciated highly. We deleted these sentences in line 295-298 following this comment, and revised the next sentence in line 298-299(see Page 15, line321-323).<br>4.Ethical statement: This needs to be included in the Materials and Methods section and needs to include approval number.<br>Answer: The ethical statement has been moved to the Materials and Methods section, and the approval number has been added (see Page 9, line 181-186).<br><br>Response to Journal Requirements:<br>1. Please ensure that your manuscript meets PLOS ONE's style requirements, including those for file naming. The PLOS ONE style templates can be found at https://journals.plos.org/plosone/s/file?id=wjVg/PLOSOne_formatting_sample_main_body.pdf and https://journals.plos.org/plosone/s/file?id=ba62/PLOSOne_formatting_sample_title_authors_affiliations.pdf<br>Answer: We ensure that our manuscript meets the journal's style.<br>2. In your methods and ethics statement, please state whether you obtained consent from parents or guardians of minors under 18 years old.<br>Answer: We have stated that the consent from parents or guardians of those under 18 years old have been obtained in the section of "ethics statement" (see Page 9, line 184-186).<br>3. PLOS ONE requires experimental methods to be described in enough detail to allow suitably skilled investigators to fully replicate and evaluate your study. See https://journals.plos.org/plosone/s/submission-guidelines#loc-materials-and-methods for more information.<br>To comply with PLOS ONE submission guidelines, in your Methods section, please provide a more detailed description of your methodology, specifically about your respiratory pathogens 15 multiplex real-time RT-PCR, Flu A/B RT-PCR, and flu typing methods.<br>Answer: We have provided a more detailed description of methodology in the section of specimen collection and laboratory testing as advised (see Page 7, line 142 to Page

Powered by Editorial Manager® and ProduXion Manager® from Aries Systems Corporation

8, line 166).
4. We note that you have indicated that data from this study are available upon request. PLOS only allows data to be available upon request if there are legal or ethical restrictions on sharing data publicly. For information on unacceptable data access restrictions, please see http://journals.plos.org/plosone/s/data-availability#loc-unacceptable-data-access-restrictions.

In your revised cover letter, please address the following prompts:
a) If there are ethical or legal restrictions on sharing a de-identified data set, please explain them in detail (e.g., data contain potentially identifying or sensitive patient information) and who has imposed them (e.g., an ethics committee). Please also provide contact information for a data access committee, ethics committee, or other institutional body to which data requests may be sent.
b) If there are no restrictions, please upload the minimal anonymized data set necessary to replicate your study findings as either Supporting Information files or to a stable, public repository and provide us with the relevant URLs, DOIs, or accession numbers. Please see http://www.bmj.com/content/340/bmj.c181.long for guidelines on how to de-identify and prepare clinical data for publication. For a list of acceptable repositories, please see http://journals.plos.org/plosone/s/data-availability#loc-recommended-repositories.
We will update your Data Availability statement on your behalf to reflect the information you provide.
Answer: We agree to provide the minimal anonymized data set as Supporting Information files for data-sharing. And Data Availability statement has been updated, and you can revise it on our behalf.
5.Your ethics statement should only appear in the Methods section of your manuscript. If your ethics statement is written in any section besides the Methods, please move it to the Methods section and delete it from any other section. Please ensure that your ethics statement is included in your manuscript, as the ethics statement entered into the online submission form will not be published alongside your manuscript.
Answer: We have moved the ethics statement to the Methods sections of manuscript.
6. Please include captions for your Supporting Information files at the end of your manuscript, and update any in-text citations to match accordingly. Please see our Supporting Information guidelines for more information:
http://journals.plos.org/plosone/s/supporting-information.
Answer: We have added captions for the Supporting Information files at the end of the revised manuscript (see Page 20, line 420-421), and updated in-text citations as advised.

Response to Reviewer #1' comments:
Reviewer #1: Dear Author
Thank you for the very nice work, indeed it generated comprehensive and very informative data. The active surveillance is much appreciated. I understand that such surveillance produced a lot of data which I believe is a big challenge to make the best out of it which you did through a very nice data presentation and analysis. In addition SARI surveillance in adult is not addressed much in the literature especially in developing areas. Moreover it seems that you described surveillance from a special geographical area characterized with unique pattern of SARI surveillance especially for the influenza B as well as the summer seasonal influenza H3 peak.

Comments:
1- The 1st letters in the title are to be capitalized.
Answer: The first letters in the title have been capitalized as advised.
Abstract
1- In the abstract line 71-73, the statement “No significant difference among … rate of main pathogens.” is unclear, please rephrase.
Answer: We have modified the statement of this sentence (see Page 3, line 62-64).
Methods
2- Line 132 please insert a reference for Sari definition.
Answer: We thank for this suggestion. A reference for SARI definition has been inserted (see Page 6, line 123).

3- Please specify details of sample collection: oropharyngeal or nasopharyngeal or

Powered by Editorial Manager® and ProduXion Manager® from Aries Systems Corporation

	both, type of the swabs used and manufacturer, VTM inhouse prepared or commercial and it's manufacturer, duration of sample storage till transportation.<br>Answer: We have specified the details of sample collection including the type of swab and manufacturer. The information of VTM manufacturer and duration of sample storage till transportation have been provided as advised (see Page 7, line 142-147).<br>4- Please specify the type of kits used : catalogue number, manufacturer or if it is inhouse made, provide primers and reagent used along with the reference.<br>Answer: The information of PCR kits has been specified (see Page 7, line 154 to Page 8, line 156). Both of the primers and reagent came from the PCR kit. The testing process of PCR was conducted according to the manufacturer's protocols.<br>5- Study subjects: Are the patients admitted in ICU or regular wards?<br>Answer: The patients in this study included those admitted in ICU, respiratory medicine department and general wards, which was specified in the Study Subject section (see Page 6, line 118-119).<br>6- Line 158-159 "Specimens were lysed at strongly denaturing conditions to deactivate RNases" please provide a reference as I believe that harsh conditions may affect the target fragile viral RNA.<br>Answer: We have followed the comment, deleted the term of "strongly" and rephrased the sentence in line 158, also, a reference has been provided according to your suggestion (see Page 7, line 151-152).<br>7- Line 160: using term "contaminant" is incorrect<br>Answer: Another reviewer thought that it was unnecessary to keep the sentence which was located in line 159-160, namely, "After adding alcohol and loading lysates onto the QIAamp spin column, viral RNA and DNA combined to the QIAamp silica membrane while contaminants passed through". We followed this suggestion and deleted this sentence which included the term of "contaminant".<br><br><br>Results<br>8- Line 237: it is not clear where did these numbers came from (20/95, 21/94) and how can the P value show significant difference between these very close findings. Please recheck and clarify.<br>Answer: The denominator (95,94) were the total number of monitoring patients in summer(Jun-Aug) and autumn(Sep-Nov) respectively, and the numerator(20,21) were the positive number of patients in summer(Jun-Aug) and autumn(Sep-Nov) respectively. As for the P value, we are sorry for negligence. The P value should be 0.83 and the difference is not significant. Thanks for point to this mistake, we have corrected it (see Page 12, line 254).<br><br>9- Line 239 and 240 please clarify what this P value indicates.<br>Answer: We have clarified the significance of this P value (see Page 12, line 254-256).<br><br>Discussion<br>10- For the significant P values, you addressed the comorbidities in the discussion. What about the dyspnea and the radiologic examination.<br>Answer: We thanks for this comment. We have addressed the dyspnea and presence of radiographic diagnosis of pneumonia in the discussion (see Page 16, line 344 to Page 17, line 364).<br>11- Findings in the result section line 224 and 225 were not discussed regarding the Xray finding in the mycoplasma and rhino causing dyspnea.<br>Answer: We thanks for this comment and have discussed them accordingly (see Page 16, line 344 to Page 17, line 357).<br>12- In the discussion, comparison of the patients from Madagascar and yours is irrelevant as they enrolled pediatric patients that were excluded from your study.<br>Answer: This comment is appreciated and we deleted this comparison in the discussion.<br>13- Line 311: You discuss cough as being the most common symptom, this is obvious as it in part of the inclusion criteria. Rather, you should address elaboration about the pneumonia and bronchiolitis.<br>Answer: We are sorry for no discussing the pneumonia in discussion on account of space limitation of original manuscript. In the revised paper, we have discussed the pneumonia and bronchiolitis following the suggestion (see Page 16, line 340-344).

Powered by Editorial Manager® and ProduXion Manager® from Aries Systems Corporation

	Figures and tables:<br>14- Figure 3: Percentage of the y axis is not clear (is it from the total enrolled or from the positive cases only). Please provide your definition of the detection rate.<br>Answer: We have clarified the significance of y axis and provided the definition of the detection rate in Fig 2 and Fig3.<br>15- Table 5: please draw lines between columns as it is confusing.<br>Answer: We have drawn lines between columns in all 5 tables according to this comment(see Table 5).<br><br>16- Table 4: Title is not informative. Significant P values need further analysis to detect the significance is between which 2 groups.<br>Answer: Title of table 4 has been revised (see Page 31, line 609-610). As for 3 variables with significant P value, we conducted the pairwise comparison (see Page 32, line 611-615). Also, we revised the statistics section accordingly (see Page 9, line 177-178).<br><br>17- Table3: It is not clear what is meant by “Chest radiographic exam”, please clarify especially that it shows significant P value and should be addressed in the discussion.<br>Answer: It means the acceptance of chest radiographic exam, we have revised it and clarified especially in bold font in table 3. And we addressed it in the discussion (see Page 17, line 357-364).<br>18- In table 2 : Percent is done from the total enrolled cases or from the positive ones. Please clarify and add the total number at the end.<br>Answer: Percent refers to the frequency of positive etiology divided by the total enrolled samples (397 cases). We have provided the explanation for it under the table 2 and added the total number at the end (see Page 29, line 590-591).<br><br><br>GENERAL:<br>19- Please specify that the surveillance addresses the community acquired infections.<br>Answer: We have specified this important significance of surveillance system in the Background section (see Page 5, line 99-101).<br>20- When you mention “Presence of radiographic diagnosis of pneumonia” you mean, lobar pneumonia denoting mostly bacterial origin, or atypical pneumonia denoting viral or atypical bacterial origin (Mycoplasma). These details need to be mentioned especially for the negative cases as they may indicate other non-tested bacterial etiology.<br>Answer: We are sorry that our case report form is the standard structural questionnaire, and it just collected the result whether has the presence of radiographic diagnosis of pneumonia, and can not show lobar pneumonia or atypical pneumonia. Meanwhile, the pathogens tested in this piloting study only covered common respiratory viruses and Mycoplasma pneumonia, and did not include respiratory bacterium. We agreed this comment and we address it in the limitation section (see Page 19, line 400-408).<br>21- Some sentences are ambiguous and need to be rephrased or corrected:<br>a. Line 149<br>Answer: The sentence in line 149 has been revised (see Page 7, line 139-140).<br>b. Line 188: remove “positive”<br>Answer: The term of “positive” in line 188 has been removed (see Page 9, line 190).<br>c. Line 191<br>Answer: The sentence in line 191 has been revised (see Page 9, line 194-195).<br>d. Line 273-274<br>Answer: The sentence in line 273-274 has been revised (see Page 14, line 295-297).<br>e. Line 295<br>Answer: The previous comment thought the sentence in line 295 did not hold, so we delete this sentence in line295-298.<br>f. Line 323<br>Answer: The “viral respiratory SARI” in line 323 has been changed to “viral SARI” (see Page 18, line 376).<br>g. Line 341<br>Answer: The sentence in line 341 has been revised (see Page 19, line 397-398).<br>h. Line 345<br>Answer: The sentence in line 345 has been revised (see Page 19, line 404-407).

Powered by Editorial Manager® and ProduXion Manager® from Aries Systems Corporation

Recommendations:
1- The title include many details that can be removed as the age group and the study period
Answer: We deleted the study period (April 2017 to March 2018) from the title following the recommendation. Meanwhile, we respect the editor's suggestion about this point. Since SARI surveillance in adults is not addressed much in the literatures especially in developing areas, we think it'd better to keep 'adult' in the title to show the difference from other studies.
2- Seasonality is better described in Epidemiologic weeks (Epi-weeks)
Answer: We respect this recommendation and it is accepted that seasonality can be described in both weeks and months. Some studies about SARI surveillance described seasonality in months, such as reference of 10 and 20. Also , our piloting study only last for 12 months and did not include enough patients. In the case of relatively small sample size of patients with confirmed pathogens, the use of weeks will make the seasonality character can not be better displayed. So we thought it is better to describe seasonality in months in order to show the characteristics of seasonality of SARI clearly.
Response to Reviewer #2' comments:
Reviewer #2: The authors described the etiological and epidemiological characters of severe acute respiratory infection caused by multiple viruses and mycoplasma pneumoniae in adult patients in Jinshan of Shanghai, April 2017 to March 2018. So befor publication there are some points need to revise as following:-

Major questions Must be clarified:-
1- Why did the authors not represent the values of real time PCR / RT-PCR for the detected pathogens as an indicator for the load of different pathogens and if there are variations among their load in relation to seasonal variation?
Answer: The PCR kit this study used is a qualitative detection kit. The detecting results were judged by Tm value of various pathogens according to melting curve. The kit didn't provide the quantitative value for the load of different pathogens. So, we are sorry that we can't state if there are variations among loads in relation of seasonal variation. We have clarified the qualitative characteristic of PCR kit in the manuscript following in this comment (see Page 7, line 154 to Page 8, line 156).
2- Only pathogens from males (173 positive cases) were statistically analyzed in relation to different variants such as type of pathogen, clinical and diagnostic parameters, age......etac Why did not authors do the same data analysis for female samples (77 positive cases) as in table 4? Also, Table 1 based manily on male cases (194) and no data concerning the female (203), why?
Answer: Please allow us to clarify these problems. Both of the differences between males and females for the proportions in table 4 and table 1 have been analyzed, and initially, we omitted to display the information of female patients on consideration of controlling the length of table. We have added a row to show the female information in table 1 and table 4.
3- Among 19 pathogens have been detected authors decided to focus on only 6 pathogens although other studies stated the predominance of other pathogens such as RSV?
Answer: This study detected 17 kinds of pathogens, in which the number of six pathogens exceeds 10. So we focus on these 6 main pathogens as the number of other seven pathogens all was fewer than 10. Table 2 described all detected pathogens. We have clarified this in the discussion (see Page 15, line 313-319).

Minor comments
1-The manuscript should be revised carefulley for typographical errors.
Answer: We have revised carefully for typographical error of the manuscript.
Abstract
2-abbravietions in line 71 should be defined at its first appearance as in line 66 then use the abbreviations
Answer: The names of viruses in line 71 have been defined with their full names at their first appearance (see Page 2, line 38 and Page 3, line 49-52). Other abbreviations in the manuscript have also been checked and revised.
3-lines 66-67 only 217 pathogens reported while in line 63 they are 250, could you mention the other type of etological agent and its frequency.
Answer: 217 pathogens in lines 66-67 refers to the total frequency of 4 main pathogens, and 250 in line 63 is the total number of patients who were identified as at

Powered by Editorial Manager® and ProduXion Manager® from Aries Systems Corporation

	least 1 pathogen. We have followed this suggestion and added the other type of etiological agents and their frequency in the abstract (see Page 3, line 51-57).<br>Background<br>4-line 100:- "owing to the lack of gold standard methods to swiftly determine etiological diagnoses" change to "owing to the lack of gold standard diagnostic methods to swiftly determine etiological agents"<br>Answer: We thanks for this suggestion and revised this sentence accordingly(see Page 4, line 84-85).<br>Materials and methods<br>5-Line 133:- "≥38˚C, cough, with onset within the last 10 days and require hospitalization" change to "≥38˚C, cough onset within the last 10 days and require hospitalization"<br>Answer: We thanks for this suggestion and revised this sentence accordingly (see Page 6, line 122-123).<br>6-Lines 137-138:- "vaccination (vaccinating influenza vaccine during 1 year before illness onset, vaccinating pneumococcal conjugate vaccine)" change to "vaccination (receiving influenza vaccine during 1 year before illness onset,and pneumococcal conjugate vaccine)"<br>Answer: We thanks for this suggestion and revised this sentence accordingly (see Page 6, line 127-128).<br>7-Line 149:- "149 information that could identify the identification of patients was masked during or after data" change to "149 information that could identify the personality of patients was masked during or after data"<br>Answer: We thanks for this suggestion and revised this sentence accordingly (see Page 7, line 139-140).<br>8-Line 157:- "viral RNA and DNA using the QIAamp Viral RNA Mini Kit (Qiagen, Hilden, Germany) following " change to "viral RNA and DNA using the QIAamp Viral RNA/DNA Mini Kit (Qiagen, Hilden, Germany) following"<br>Answer: We are sorry for this negligence and revised this sentence according to the suggestion (see Page 7, line 148-150).<br>9-Lines 161-162:- "Total nucleic acid extracts were further processed by multiplex real-time reverse transcription" change to "Viral nucleic acid extracts were further processed by multiplex real-time reverse transcription" since you used kit for viral nucleic acid (RNA or DNA)<br>Answer: We thanks for this suggestion and revised this sentence accordingly(see Page 7, line 153-154).<br>10-Lines 163-163:- "Respiratory pathogens 15 multiplex real-time RT-PCR diagnostic strategy was adopted to detect PIV (types 1, ..." change to "The multiplex real-time RT-PCR diagnostic strategy was adopted to detect 15 respiratory pathogens, PIV (types 1, ..."<br>Answer: We thanks for this suggestion and revised this sentence following the suggestion (see Page 7, line 154 to Page 8, line 156).<br>Results<br>11-As general when you describe the results please make full description of the full cases either positive or not and do not leave unclear such as line 212 you mentioned 382 cases and ignored the residue 15 cases and this was repeated allover the manuiscript, do not leave anything for guessing.<br>Answer: We thank for this suggestion, and have tried our best to clarify these unclear descriptions all over the manuscript as advised (see Page 9, line 191-197; Page 10, line 199-200; Page 11, line 222-225; Page 13, line 277-280).<br><br>12-Lines 199-203:- Authors described the frequency and type of pathogens,however in compare to table 2 there is confusion concerning the pathogen frequency as in text 198 singl and 52 multiple, while later on the number will be 232 and in table 312, how can this occur? please clarify this.<br>Answer: Number of 198 and 52 in line 199 were the number of patients with single and multiple infections, respectively. Numbers from line 201 to line 203 including 95 (M. pneumoniae), 46 (AdV), 44 (Flu A/H3N2), 32 (HRhV), 25 (Flu B/Yamagata) represent the frequency of identified pathogen which was detected most frequently, and their meaning was different from that in line 199.Numbers from the 3rd row(16 for Flu A/pH1N1) to the 25th row(95 for M. pneumoniae) in table 2 also represent the frequency of identified pathogens and their total number equals to 312. We have revised the corresponding description in section of etiology (see Page 10, line 206-214), and added the explanation for frequency under the table 2.

	13-lines 213-215:- "Thirty-two SARI patients and 30 patients had exposure of contacting with patients with fever and respiratory symptoms and contacting with live poultry during 2 weeks before their illness onset, respectively" change to "Thirty-two SARI patients had exposures with fever and respiratory symptoms patients while 30 SARI patients contacted with live poultry during 2 weeks before their illness onset"<br>Answer: We thanks for this suggestion and revised this sentence following the suggestion (see Page 11, line 225-227).<br>Tables<br>1- Table 1 1st row change " All SARI SARI patient with confirmed pathogens SARI patient without confirmed pathogens" to "All with confirmed pathogens without confirmed pathogens" and add SARI patient above as another row.<br>Answer: We have revised the 1st row of Table 1 and added SARI patient above as another row following this suggestion (see Table1).<br>2- Table 2 1st clonumn please change "viral etiology" to "etiology" only because there is a bacteria also mentioned there.<br>Answer: We are sorry for this negligence and have changed it according to the suggestion (see Table 2).<br>3- Table 3 1st row change " All SARI SARI patient with confirmed pathogens SARI patient without confirmed pathogens" to "All with confirmed pathogens without confirmed pathogens" and add SARI patient above as another row. Visiting a live poultry market and Contact with live poultry in table 3 looks the same where in table 4 it become one catogery Contact with live poultry.<br>Answer: We have revised the 1st row of Table 3 and added SARI patient above as another row following this suggestion, so does the Table 5. Contact with live poultry included contacting with live poultry at home and other place (such as live poultry market), so it is different from visiting a live poultry market. Since the number of patients visiting a live poultry market was just 3 cases, and it only included 1 case with single-infected M. pneumoniae positivity and 1 case with single-infected AdV positivity, the third case belonged to multiple infections, so the initial table 4 didn't analyze this variable. We have analyzed it in table 4 according to this comment (see Table 4).<br>Figures<br>The presented pathogens in Fig 1-3 based only on male SARI cases with confirmed pathogens or included all pathogens from male and female cases.<br>Answer: The pathogens in Fig1-3 based on all SARI cases with confirmed pathogens including male and female cases.
**Additional Information:**	
**Question**	**Response**
**Financial Disclosure**<br><br>Enter a financial disclosure statement that describes the sources of funding for the work included in this submission. Review the submission guidelines for detailed requirements. View published research articles from *PLOS ONE* for specific examples.<br><br>This statement is required for submission and **will appear in the published article** if the submission is accepted. Please make sure it is accurate.	This work was supported by the Research Project of Shanghai Municipal Health Commission (201940428) for Can-Lei Song and the Infectious Disease and Epidemiology Project of the 6th Jinshan District Medical Key Specialty Construction (JSZK2019B05) for Shu-Hua Li . The funder had no role in study design, data collection and analysis, decision to publish or preparation of manuscript.

**Unfunded studies**<br>Enter: *The author(s) received no specific funding for this work.*<br><br>**Funded studies**<br>Enter a statement with the following details:<br>• Initials of the authors who received each award<br>• Grant numbers awarded to each author<br>• The full name of each funder<br>• URL of each funder website<br>• Did the sponsors or funders play any role in the study design, data collection and analysis, decision to publish, or preparation of the manuscript?<br>• **NO** - Include this sentence at the end of your statement: *The funders had no role in study design, data collection and analysis, decision to publish, or preparation of the manuscript.*<br>• **YES** - Specify the role(s) played.<br><br>* typeset	
**Competing Interests**<br><br>Use the instructions below to enter a competing interest statement for this submission. On behalf of all authors, disclose any competing interests that could be perceived to bias this work—acknowledging all financial support and any other relevant financial or non-financial competing interests.<br><br>This statement **will appear in the published article** if the submission is accepted. Please make sure it is accurate. View published research articles from *PLOS ONE* for specific examples.	The authors have declared that no competing interests exist.

Powered by Editorial Manager® and ProduXion Manager® from Aries Systems Corporation

**NO authors have competing interests**<br><br>Enter: *The authors have declared that no competing interests exist.*<br><br>**Authors with competing interests**<br><br>Enter competing interest details beginning with this statement:<br><br>*I have read the journal's policy and the authors of this manuscript have the following competing interests: [insert competing interests here]*<br><br>* typeset	
**Ethics Statement**<br><br>Enter an ethics statement for this submission. This statement is required if the study involved:<br><br>• Human participants<br>• Human specimens or tissue<br>• Vertebrate animals or cephalopods<br>• Vertebrate embryos or tissues<br>• Field research<br><br>Write "N/A" if the submission does not require an ethics statement.<br><br>General guidance is provided below. Consult the submission guidelines for detailed instructions. **Make sure that all information entered here is included in the Methods section of the manuscript.**	This study belonged to the part of hospital-based surveillance program of SARI of Shanghai, and approved by the ethical review committee of the Shanghai Municipal Center for Disease Control and Prevention. Written informed consent was obtained from patients or proxies before enrollment.

Powered by Editorial Manager® and ProduXion Manager® from Aries Systems Corporation

**Format for specific study types**<br><br>**Human Subject Research (involving human participants and/or tissue)**<br>• Give the name of the institutional review board or ethics committee that approved the study<br>• Include the approval number and/or a statement indicating approval of this research<br>• Indicate the form of consent obtained (written/oral) or the reason that consent was not obtained (e.g. the data were analyzed anonymously)<br><br>**Animal Research (involving vertebrate animals, embryos or tissues)**<br>• Provide the name of the Institutional Animal Care and Use Committee (IACUC) or other relevant ethics board that reviewed the study protocol, and indicate whether they approved this research or granted a formal waiver of ethical approval<br>• Include an approval number if one was obtained<br>• If the study involved *non-human primates*, add *additional details* about animal welfare and steps taken to ameliorate suffering<br>• If anesthesia, euthanasia, or any kind of animal sacrifice is part of the study, include briefly which substances and/or methods were applied<br><br>**Field Research**<br><br>Include the following details if this study involves the collection of plant, animal, or other materials from a natural setting:<br>• Field permit number<br>• Name of the institution or relevant body that granted permission	
**Data Availability**<br><br>Authors are required to make all data underlying the findings described fully available, without restriction, and from the time of publication. PLOS allows rare exceptions to address legal and ethical concerns. See the PLOS Data Policy and FAQ for detailed information.	No - some restrictions will apply

A Data Availability Statement describing where the data can be found is required at submission. Your answers to this question constitute the Data Availability Statement and **will be published in the article**, if accepted.<br><br>**Important:** Stating 'data available on request from the author' is not sufficient. If your data are only available upon request, select 'No' for the first question and explain your exceptional situation in the text box.<br><br>Do the authors confirm that all data underlying the findings described in their manuscript are fully available without restriction?	
**Describe where the data may be found in full sentences. If you are copying our sample text, replace any instances of XXX with the appropriate details.**<br><br>• If the data are **held or will be held in a public repository**, include URLs, accession numbers or DOIs. If this information will only be available after acceptance, indicate this by ticking the box below. For example: *All XXX files are available from the XXX database (accession number(s) XXX, XXX.)*.<br>• If the data are all contained **within the manuscript and/or Supporting Information files**, enter the following: *All relevant data are within the manuscript and its Supporting Information files.*<br>• If neither of these applies but you are able to provide **details of access elsewhere**, with or without limitations, please do so. For example:<br><br>*Data cannot be shared publicly because of [XXX]. Data are available from the XXX Institutional Data Access / Ethics Committee (contact via XXX) for researchers who meet the criteria for access to confidential data.*<br><br>*The data underlying the results presented in the study are available from (include the name of the third party*	All relevant data are contained within the manuscript and its Supporting Information files.

and contact information or URL).<br>• This text is appropriate if the data are owned by a third party and authors do not have permission to share the data.<br><br>* typeset	
Additional data availability information:	

Powered by Editorial Manager® and ProduXion Manager® from Aries Systems Corporation

Manuscript Click here to access/download;Manuscript;Manuscript.docx

Etiological and Epidemiological Characteristics of Severe Acute Respiratory Infection Caused by Multiple Viruses and *Mycoplasma Pneumoniae* in Adult Patients in Jinshan, Shanghai: A Pilot Hospital-based Surveillance Study

Jian Li [1†], Can-Lei Song [2†], Tang Wang [2†], Yu-Long Ye [3], Jian-Ru Du [3], Shu-Hua Li [2], Jian-Min Zhu[2]

[†] These authors contributed equally to this work.

[1]*Clinical Research Center, Ruijin Hospital, Shanghai Jiao Tong University School of Medicine, Shanghai, China*

[2] *Department of Acute Infectious Diseases Control, Jinshan District Center for Diseases Control and Prevention, Shanghai, China*

[3]*Department of Microbiology, Jinshan District Center for Diseases Control and Prevention, Shanghai, China*

Email addresses:

Jian Li : nclijian@163.com

Can-Lei Song: 629309340@163.com

Tang Wang: 1090595528@qq.com

Yu-Long Ye: yeyulong99@163.com

Jian-Ru Du: 646947222@qq.com

Shu-Hua Li: hsli167@163.com

Jian-Min Zhu: zhujm12@163.com

Corresponding author:

Dr. Jian Li: Clinical Research Center, Ruijin Hospital, Shanghai Jiao Tong University School of Medicine, 197 Ruijin Er Rd, Huangpu District, Shanghai, China, 200025

Dr. Jian-Min Zhu: Department of Acute Infectious Diseases Control, Jinshan District Center for Diseases Control and Prevention, 94 Weisheng Rd, Jinshan District, Shanghai, China, 201599

Abstract

Background

Severe acute respiratory infection (SARI) results in a tremendous disease burden worldwide. Available research on active surveillance among hospitalized adult patients suffering from SARI in China is limited. This pilot study aimed to identify associated etiologies and describe the demographic, epidemiological and clinical profiles of hospitalized SARI patients aged over 16 years in Jinshan, Shanghai.

Methods

Active surveillance was conducted at 1 sentinel hospital in Jinshan district, Shanghai, from April 2017 to March 2018. Hospitalized SARI patients aged more than 16 years were enrolled, and nasopharyngeal swabs were collected within 24 hours of admission and tested for multiple respiratory viruses (including 18 common viruses) and *Mycoplasma pneumoniae* (*M. pneumoniae*) with real-time polymerase chain reaction. Demographic, epidemiological and clinical information was obtained from case report forms.

Results

In total, 397 SARI patients were enrolled; the median age was 68 years, and 194 (48.9%) patients were male**.** A total of 278 (70.0%) patients had at least one underlying chronic medical condition**.** The most frequent symptoms were cough (99.2%) and sputum production (88.4%). The median duration of hospitalization was 10 days. A total of 250 infection patients (63.0%) were positive for

at least one pathogen, of whom 198 (49.9%) were positive for a single pathogen and 52 (13.1%) were positive for multiple pathogens. The pathogens identified most frequently were *M. pneumoniae* (23.9%, 95/397), followed by adenovirus (AdV) (11.6%, 46/397), influenza virus A/H3N2 (Flu A/H3N2) (11.1%, 44/397), human rhinovirus (HRhV) (8.1%, 32/397), influenza virus B/Yamagata (Flu B/Yamagata) (6.3%, 25/397), pandemic influenza virus A/H1N1 (Flu A/pH1N1) (4.0%, 16/397), parainfluenza virus (PIV) type 1 (2.0%, 8/397), human coronavirus (HCoV) type NL63 (2.0%, 8/397), HCoV type 229E (1.5%, 6/397), HCoV type HKU1 (1.5%, 6/397), PIV type 3 (1.5%, 6/397), human metapneumovirus (HMPV) (1.5%, 6/397), PIV type 4 (1.3%, 5/397), HCoV type OC43 (1.0%, 4/397), influenza virus B/Victoria (Flu B/Victoria) (0.5%, 2/397), respiratory syncytial virus (RSV) type B (0.5%, 2/397), and human bocavirus (HBoV) (0.3%, 1/397). The seasonality of pathogen-confirmed SARI patients had a bimodal distribution, with the first peak in summer and the second peak in winter. Statistically significant differences were observed with respect to the rates of dyspnea, radiographically diagnosed pneumonia and the presence of at least one comorbidity in patients who were infected with only *M. pneumoniae*, AdV, HRhV, Flu A/H3N2, Flu A /pH1N1 or Flu B/Yamagata. The differences in the positivity rates of the above 6 pathogens among the different age groups were nonsignificant.

Conclusions

M. pneumoniae, AdV and Flu A/H3N2 were the main pathogens detected in hospitalized SARI patients aged more than 16 years in Jinshan district, Shanghai. Our findings highlight the importance of sustained multipathogen surveillance among SARI patients in sentinel hospitals, which can provide useful information on SARI etiologies, epidemiology, and clinical characteristics.

Key words: Severe acute respiratory infection, sentinel surveillance, pathogen, epidemiology

Background

Severe acute respiratory infection (SARI) has been considered an important contributor to morbidity and mortality in all age groups, particularly children, elderly individuals and individuals with compromised immune, cardiac and pulmonary systems, worldwide [1-3]. It is estimated that SARI causes approximately 4.2 million deaths annually. Of these, up to 90% are believed to occur in developing countries [4]. Various viral and bacterial pathogens are associated with SARI. Due to their extremely high potential for human-to-human transmission, these pathogens pose a substantial risk to human health. While bacterial infection has a substantial influence on the development of severe pneumonia [5], a significant proportion of SARIs are attributed to viral pathogens, such as influenza viruses A and B (Flu A/B), parainfluenza viruses (PIV), adenoviruses (AdVs), respiratory syncytial viruses (RSVs), human coronaviruses (HCoVs) and human rhinoviruses (HRhVs) [6]. Nevertheless, owing to the lack of gold standard diagnostic methods to rapidly identify etiological agents, most patients are treated with antibiotics empirically [7]. Rapid etiologic diagnosis therefore remains a significant public health challenge.

Routine pathogen monitoring is critical for preparedness for and response to the SARI epidemic. Since SARI is the leading cause of hospitalization in children under the age of 5 years and of febrile episodes in infants younger than 3 months old, most available studies regarding the burden of SARI focus on viral infections in children [8-11]. A SARI surveillance study in China revealed that 90% of patients were aged <15 years [12]. In addition, the majority of the data on the epidemiology of the etiologic agents of SARI was collected in developed regions. The epidemiological characteristics and distributions of the major viral pathogens in adult SARI

patients are still limited in developing regions [13]. *Mycoplasma pneumoniae* (*M. pneumoniae*) has long been considered an important etiology of respiratory disease and is more frequently isolated among children and young adults [14, 15]. Research on active surveillance in hospitalized adult patients suffering from SARI in China is scarce. Accordingly, a pilot study on active surveillance of SARI was initiated to characterize community-acquired pulmonary infections and monitor the epidemiologic and etiologic characteristics of SARI caused by various viral pathogens and *M. pneumoniae* in adult inpatients in Jinshan district, Shanghai, in April 2017. The aim of the present study was to characterize the demographic and epidemiologic characteristics of SARI, identify the etiologies and assess the clinical profiles associated with SARIs in hospitalized adult patients in Jinshan, Shanghai, by performing 12 months of active surveillance.

Materials and methods

Study setting

Jinshan district is a suburb located in southwest Shanghai, P.R. China. Active surveillance was initiated at Jinshan District Central Hospital in April 2017 and was conducted for 12 months. This hospital was selected because it is one of the largest general hospitals in the district and a national surveillance sentinel site for influenza virus. It serves most of the population of Jinshan district, with a total of 636 beds. In 2017, the registered population in Jinshan district was 523,641, of which 467,320 (89.24%) were adults aged more than 18 years [16].

Study subjects

All patients aged over 16 years who were admitted to the intensive care unit, respiratory medicine department and general wards in the hospital were screened by a trained physician between April 2017 and March 2018. Patients were diagnosed with SARI according to the World Health

Organization (WHO) definition, which includes acute respiratory infection with a measured fever of ≥38°C, cough onset within the last 10 days and required hospitalization [1].

Data collection

After hospital admission, a standard case report form was completed for each eligible patient. The form comprised information on demographic characteristics (sex, age, weight, height, residence), vaccination (received an influenza vaccine 1 year before illness onset, ever received a pneumococcal conjugate vaccine), admission diagnosis, comorbidities (asthma, chronic bronchitis, chronic obstructive pulmonary disease (COPD), hypertension, diabetes, cardiovascular disease, tumor), clinical presentation (fever, cough, difficult breathing, sore throat), antibiotic treatments prior to hospitalization, exposure history (smoking, visiting a live poultry market, contact with live poultry, contact with a patient with fever and respiratory symptoms within 2 weeks before illness onset). At discharge, the form was updated to include information about treatment in the hospital, chest computed tomographic (CT) findings, complications and prognosis. Data were collected by the trained physician. To ensure the accuracy of the data, spouses or caregivers who lived with the patients for more than 2 weeks before illness onset were interviewed, and the medical records of the patients were reviewed. Two radiologists interpreted chest CT scans independently. In the case of a disagreement, a third radiologist was consulted to reach a final decision. All the information that could identify the personality of patients was masked during or after data collection.

Specimen collection and laboratory testing

A single flocked polyester nasopharyngeal swab (Becton Dickinson, USA, MD) sample was collected from each SARI patient by a nurse within 24 hours of admission following a standard procedure. The swab was inserted into a cryovial containing 3 ml of viral transport medium

(Tiandz, China, Beijing). The specimens were stored at 4°C in the hospital and transferred within 24 hours of collection to the laboratory at Jinshan District Center for Disease Control and Prevention (CDC), where they were preserved at -70°C until testing was performed. Viral RNA and DNA were extracted from 200-μl samples using the QIAamp Viral RNA/DNA Mini Kit (Qiagen, Hilden, Germany) following the manufacturer's instructions. To guarantee integrity, specimens were lysed under denaturing conditions to deactivate RNases [1]. Pure viral RNA and DNA were eluted in 60 μl of low-salt buffer, and impurities were removed. Viral nucleic acid extracts were further processed by multiplex real-time reverse transcription polymerase chain reaction (RT-PCR). The qualitative RespiFinder 2SMART multiplex real-time RT-PCR diagnostic strategy (Geneodx, Shanghai, China) was adopted to detect 15 respiratory pathogens, including PIV (types 1, 2, 3 and 4), HCoV (types 229E, OC43, HKU1 and NL63), RSV (types A and B), HRhV, AdV, human metapneumovirus (HMPV), human bocavirus (HBoV) and *M. pneumoniae*, using the CFX96™ real-time PCR system (Bio-Rad, Hercules, CA, USA) according to the manufacturer's protocols. In addition, RNA from each specimen was identified for specific primers and probes that target Flu A/B using real-time RT-PCR following the US CDC's protocol. Specimens that were positive for Flu A and Flu B were subsequently subtyped for pandemic influenza virus A/H1N1 (Flu A/pH1N1) and seasonal influenza virus A/H3N2 (Flu A/H3N2) and Flu B/Yamagata and Flu B/Victoria, respectively [17]. These tests were performed in the biosafety level 2 laboratory of the Jinshan CDC.

Statistics

The collected data were double-entered into a database constructed in EpiData 3.1. Logic checks to assess the quality of data entry were conducted. Single infection was defined as infection

caused by one pathogen, and multiple infection was defined as infection caused by at least 2 pathogens (virus/virus, virus/*M. pneumoniae*) in a single specimen. Continuous data are reported as medians and interquartile ranges (IQRs), and the Mann-Whitney U test was used to compare differences between groups. Categorical data are expressed as frequencies and proportions, and the chi-squared test or Fisher's exact test, as appropriate, was used to compare patients with and without confirmed pathogens in terms of demographics, clinical characteristics, epidemiologic characteristics, treatment and prognosis. Bonferroni's correction was used for pairwise comparisons. For proportions, the binomial 95% confidence interval is reported. The analysis was performed using SPSS v. 25.0 (IBM Corporation, Armonk, NY, USA), and all tests were two-sided with a 5% significance level.

Ethics statement

This study was part of a hospital-based SARI surveillance program in Shanghai and was approved by the ethical review committee of the Shanghai Municipal Center for Disease Control and Prevention (Ref #: 2015-14). Written informed consent was obtained from patients or proxies before enrollment and from parents or guardians of those under 18 years old. This study was conducted in accordance with the Declaration of Helsinki.

Results

Demographic characteristics

From April 2017 to March 2018, a total of 397 patients meeting the SARI case definition were admitted to our hospital. One or more pathogens were detected in 250 patients (63.0%; 95% CI: 58.2-67.7%), and negative results were obtained from the remaining 147 patients. The median age of the patients was 68 years (IQR: 59-78; range: 16 to 99 years). Among the SARI patients, 194

(48.9%) were male, and 203 (51.9%) were female. The majority of patients were elderly patients aged 60 or more years (295 cases), accounted for 74.3% of the total patients; 58 (14.6%) patients were 40-59 years of age, and 19 (4.8%) patients were 30-39 years of age. Those less than 30 years old represented only 6.3% of the total patients (25 cases). The percentages of patients with a body mass index (BMI) <20, between 20 and 25, and >25 were 29.7%, 52.4% and 17.9%, respectively. A total of 278 SARI patients (70.0%) had at least one comorbidity, and 119 patients had no comorbidity (Table 1). There were no significant differences in sex, age, BMI and underlying chronic medical conditions between SARI patients with confirmed pathogens and those without confirmed pathogens (P>0.05).

Etiologies

Of the 397 SARI patients, 198 (49.9%; 95% CI: 45.0-54.8%) patients had single infection, while 52 (13.1%; 95% CI: 9.8-16.4%) patients had multiple infection. The most prevalent pathogen identified was *M. pneumoniae* in 95 (23.9% of the total samples) patients, followed by AdV in 46 (11.6%) patients, Flu A/H3N2 in 44 (11.1%) patients, HRhV in 32 (8.1%) patients, Flu B/Yamagata in 25 (6.3%) patients, and Flu A /pH1N1 in 16 (4.0%) patients. Other viruses, including PIV type 1, HCoV type NL63, HCoV type 229E, HCoV type HKU1, PIV type 3, HMPV, PIV type 4, HCoV type OC43, Flu B/Victoria, RSV type B and HBoV, were detected in a 0.3% to 2.0% of samples (Table 2). The most frequently detected pathogens in patients with multiple infection were *M. pneumoniae* (84.6%, 44/52), AdV (28.8%, 15/52), HRhV (25.0%, 13/52), and Flu A/H3N2 (17.3%, 9/52).

Clinical and epidemiologic characteristics

Pneumonia (222 cases, 55.9%) was the most common clinical diagnosis made by clinicians on

admission, followed by bronchiolitis (68 cases, 17.1%). The most common symptoms on admission were cough (99.2%) and sputum production (88.4%), followed by thoracalgia (7.1%) and pharyngalgia (6.8%). Of the 397 SARI patients, a temperature ≥ 39°C was recorded in 189 SARI patients (47.6%) on admission. A total of 382 patients (96.2%) underwent chest CT, of whom 258 (67.5%) were reported to have radiographic evidence of pneumonia; the remaining 15 patients did not undergo chest CT examination. Thirty-two SARI patients had exposure to a patient with fever and respiratory symptoms, while 30 SARI patients had contact with live poultry 2 weeks before illness onset. Among the 397 patients, only 5 patients had received a pneumococcal conjugate vaccine, and 1 patient was vaccinated against influenza (Table 3). No significant differences in the proportions of clinical and epidemiologic characteristics between SARI patients with confirmed pathogens and those without confirmed pathogens were found, except for chest radiographic examination findings. As illustrated in Table 4, the differences in the proportions of dyspnea, radiographic diagnosis of pneumonia and the presence of at least one comorbidity among patients infected with only one of the 6 main pathogens, including *M. pneumoniae*, AdV, HRhV, Flu A/H3N2, Flu A /pH1N1 and Flu B/Yamagata, were statistically significant. Notably, the proportion of patients with radiographic evidence of pneumonia was highest in patients infected by *M. pneumonia* (74.5%), and dyspnea was the most common presentation in patients with HRhV (21.1%).

Seasonal trends

Figure 1 shows monthly variations in the number of SARI patients infected with *M. pneumoniae*, AdV, Flu A/H3N2, Flu A /pH1N1, HRhV, and Flu B/Yamagata. Over the 12-month period, the temporal distribution of pathogen-confirmed SARI patients had a bimodal shape, with the first

peak in the summer and the second peak in the winter. The duration of the first positive peak was 2 months, from August to September, but the second peak lasted only 1 month. The infection peaks seemed to be attributable to the number of *M. pneumoniae* and AdV cases detected. In addition, Flu A/H3N2 contributed to the summer peak, whereas Flu B/Yamagata and Flu A/pH1N1 dominantly contributed to the winter peak. Unlike other pathogens, HRhV was detected all year along and did not show apparent seasonality. The distributions of the seasonal patterns of the positivity rates of the main 6 pathogens are shown in Figure 2. Flu A/H3N2 prevalence peaked in summer (Jun-Aug) and autumn (Sep-Nov), with positivity rates of 21.1% (20/95) and 22.3% (21/94), respectively (P>0.05). However, Flu A/pH1N1 and Flu B/Yamagata peaked in winter (Dec-Feb), with positivity rates of 9.8% (13/132) and 18.9% (25/132), respectively; the differences were statistically significant (P<0.01). It is worth noting that no SARI patients infected by Flu B/Yamagata were detected in spring (Mar-May), summer or autumn. The positivity rate of *M. pneumoniae* was significantly higher in autumn (43.6%, 41/94) than in other seasons (P<0.01). The positivity rate (18.4%, 14/76) of HRhV was significantly higher in spring than that in the other seasons (P<0.01). The positivity rate of AdV did not demonstrate obvious seasonality throughout the year (P>0.05).

Age distribution

The age group distributions of the positivity rates of the main pathogens, *M. pneumoniae*, AdV, Flu A/H3N2, Flu A/pH1N1, HRhV, and Flu B/Yamagata, are shown in Figure 3. The prevalence rates of Flu A/pH1N1 (8.0%) and AdV (20.0%) peaked in the group younger than 30 years old, although the difference was not significant (P>0.05). The positivity rates of *M. pneumonia* (36.2%) and Flu B/Yamagata (6.9%) were the highest in the 40-59-year-old group, without statistical

significance (*P*>0.05). Moreover, no significant differences among the different age groups were observed with regard to the positivity rates of Flu A/H3N2 and HRhV. Interestingly, no patients infected with Flu A/H3N2 and HRhV were detected in the 30- to 39-year-old group.

Treatment and prognosis

The median duration from illness onset to admission in SARI patients was 3 days (IQR: 2-5.5; range: 0 to 14 days), and the median duration of hospitalization was 10 days (IQR: 8-13 days). Complications occurred in 61 SARI patients, with electrolyte metabolism disorder (19 cases), respiratory failure (14 cases) and cardiac insufficiency (8 cases) being the most common complications. The remaining 336 patients did not report any complications. No significant differences between SARI patients with confirmed pathogens and those without confirmed pathogens were observed with regard to the use of antibiotics (levofloxacin, cephalosporin, azithromycin), antivirals (oseltamivir), glucocorticoids and oxygen therapy (*P*>0.05). The duration of antibiotic use during hospitalization was 1-15 days (median: 9 days [IQR 5-11]) in SARI patients without confirmed pathogens and 1-20 days (median: 9 days [IQR 6-11]) in those with confirmed pathogens, though the difference was nonsignificant (*P*=0.68). Three SARI patients died during hospitalization (Table 5).

Discussion

Hospital-based sentinel surveillance of SARI can be used as a strategy to monitor trends in this relatively severe disease and is critical for establishing a platform to understand the epidemiologic and etiologic profiles at the local level. A monitoring study involving SARI patients in Georgia demonstrated that the proportions of patients positive for respiratory pathogens varied widely between seasons; there was no influenza detected in summer and early autumn (from July to

October) but a 30% RSV positivity rate from March 2015–2017[1]. Another surveillance study involving SARI patients in several countries found that the positivity rates of influenza viruses varied widely depending on country and season, from 2.1% in Armenia in 2011–2012 to 100% in Albania in 2009–2010 [18]. A comparative study of viral profiles in hospitalized pediatric SARI patients in Beijing and Shanghai, China, showed different viral profile patterns in the 2 cities; RSV (52.9%) and HRhV/enterovirus (34.7%) were the most prevalent etiological agents of SARI in Beijing, whereas HRhV/enterovirus (33.6%) and HBoV (17.7%) were the main pathogens of SARI in Shanghai [10]. The early detection of divergent SARI pathogens through sentinel surveillance can measure the burden of disease on the basis of severity and better prepare a region for an emergency response. To our knowledge, this pilot study is the first study to continuously surveil 19 respiratory pathogens in adult SARI patients in Shanghai, eastern China, providing an improved understanding of the epidemiology, etiologic spectrum and clinical profile of SARI. During 1 year of active surveillance, 397 patients who met the established case definition of SARI were eligible for enrollment in this study, and 63.0% of these patients tested positive for at least one pathogen. Our findings were in accordance with those reported elsewhere, which revealed etiologies in 50% to 85% of hospitalized SARI cases [7, 19-20].

From April 2017 to March 2018 in Jinshan district, the main etiologies of SARI varied seasonally; *M. pneumoniae*, AdV, Flu A/H3N2, HRhV, Flu B/Yamagata, and Flu A/pH1N1 were the predominant pathogens depending on the month. Other viruses, such as PIV type 1, HCoV type NL63, HCoV type 229E, HCoV type HKU1, PIV type 3, HMPV, PIV type 4, HCoV type OC43, Flu B/Victoria, RSV type B and HBoV, were also present, although the numbers of patients infected with these viruses were relatively small. Since our surveillance system aimed to detect

SARI in adult patients, most of the enrolled patients were elderly individuals aged 60-79 years (52.1%) 80 years and above (22.2%). Our study demonstrates that individuals in the over 60 age group are the most vulnerable to SARI in Jinshan, a subtropical region. In the present study, at least one chronic medical condition was present in 70% of SARI patients. Our study population had a high prevalence of comorbidities compared with that in a study in Hubei Province, China [12]. This may be partially explained by the inconsistency in socioeconomic development between the 2 regions. Hypertension and cardiovascular disease were observed in 38.3% and 7.6% of our population, respectively. Patients with confirmed pathogens had a higher prevalence of cardiovascular disease than those without confirmed pathogens. One study suggested that diagnosed cardiovascular disease was related to a fatal outcome in influenza-positive SARI patients [21]. Our study revealed that the proportions of patients who received influenza and pneumococcal conjugate vaccines were quite low, so respiratory disease vaccination programs targeting individuals with cardiovascular-related diseases should be recommended. In this study, most patients presented with cough, sputum production and fever. These clinical features bear some resemblance to those reported in a previous study [1]. It should be noted that empirical administration of antibiotics during hospitalization occurred in 99% of patients in the present study due to the unavailability of rapid pathogen identification tests. The current study found that pneumonia was the main reason for hospital admission of SARI patients (55.9%), followed by bronchiolitis (17.1%) in Jinshan, a region in eastern China. A similar study in northern China showed that pneumonia (88.95%) and bronchiolitis (6.37%) were also the top 2 admission diagnoses among SARI patients [22]. HRhV has emerged as an independent causative agent of lower respiratory tract infection. To date, the majority of investigations on HRhV-associated lower

respiratory tract infection in adults have focused on immunocompromised patients [23-25] or those with hospital-acquired pneumonia [26-27]. We compared the patients with single infection in terms of signs and symptoms, and the results showed that dyspnea was the most frequent symptom (21.1%) in community-acquired SARI patients infected by HRhV, which was consistent with the results of a similar multicenter study (30%) in China [28]. *M. pneumoniae* is an important cause of community-acquired pneumonia. Depending on the setting, 10%-40% of community-acquired pneumonia patients are infected with *M. pneumoniae* [20]. Our study also showed that patients infected by *M. pneumoniae* had the highest rate of radiographic evidence of pneumonia (74.5%) compared with those infected by other single pathogens, demonstrating that community-acquired pneumonia is a heterogeneous disease. Among the 382 SARI patients who underwent chest CT, there was a significant difference in the proportion of patients who accepted a chest radiographic examination between SARI patients with confirmed pathogens and those without confirmed pathogens. However, a significant difference in the proportion of patients presenting a radiographic diagnosis of pneumonia between SARI patients with confirmed pathogens and those without confirmed pathogens was not observed, suggesting that the etiologies and disease courses of community-acquired pneumonia were highly variable.

M. pneumoniae (23.9%) was the most common pathogen in the present study. The positive detection rate of *M. pneumoniae* was similar to the published rate (19.7%) in northern China [20]. A prospective study in Hong Kong including adults hospitalized with pneumonia from 2004 to 2005 found that *M. pneumoniae* was detected in 78/1,193 patients (6.5%) [29]. *M. pneumoniae* occurs endemically worldwide in many different geographic regions. *M. pneumoniae* was mostly detected in autumn (43.6%) and spring (27.6%) in our study, but *M. pneumoniae* in

Istanbul was more commonly identified in summer (44.9%) and winter (22.4%) [30]. As the second most common pathogen in this study, the positivity rate of AdV did not significantly differ seasonally; this trend in seasonality was consistent with previously reported AdV seasonality data from China [10]. In contrast with the seasonality of viral SARI observed in Georgia in 2015-2017 and in northern China in 2014-2016, where a distinct winter-only influenza peak was observed [31,32], we found that influenza peaked in both the winter and in summer. Overall, influenza virus was common in this study, with Flu A/H3N2 dominating in summer and Flu B/Yamagata and Flu A/pH1N1 dominating in winter. According to our findings, the positivity rate of Flu B/Yamagata (18.9%) was nearly twice that of Flu A/pH1N1 (9.8%) in winter; this result was different from that of a study in the USA in which estimated excess hospitalization rates associated with influenza B were lower than those associated with Flu A/H3N2 [33]. In this study, we also noted that no significant differences were found in the positivity rates *M. pneumoniae*, AdV, Flu A/H3N2, Flu A /pH1N1, HRhV, and Flu B/Yamagata among the different age groups. This result was basically the same as that in a previous study in China [10] and may be attributable to susceptibility to these common viruses in different age groups of adults. As reported elsewhere [34], coinfections were relatively common in the present study. A total of 13.1% of SARI patients were reported to have more than one pathogen infection; this percentage was consistent with that in a previous study (11.7%) [19].

Limitations

Our study was subject to several limitations. First, as a pilot study, this study was conducted at only 1 hospital. Even though this hospital is the largest hospital in the district, the findings may have relatively limited generalizability. The prevalence of each pathogen may vary in regions with

different climates, demographic patterns and accessibility to healthcare. Second, the result was based on SARI surveillance over a 12-month period, and the burden due to SARI may not reflect the actual situation over several years. Third, the case report form in this study was a standard structured questionnaire, and the results were collected to determine whether the patient had received a radiographic diagnosis of pneumonia. It was impossible to pinpoint the type of pneumonia, such as lobar pneumonia or atypical pneumonia. The pathogens detected in this pilot study covered only common respiratory viruses and *M. pneumoniae* and did not include related respiratory bacterial pathogens, such as *Pneumococcus* and *Bordetella pertussis*, owing to limited financial support, so SARI patients without confirmed pathogens may have been positive for other nontested bacterial pathogens. Indeed, the inclusion of bacterial surveillance is under consideration for integration into our program.

Conclusions

In conclusion, the current study was the first to monitor hospitalized adult SARI patients for most respiratory viruses and *M. pneumoniae* in Shanghai and confirmed that multiple respiratory pathogens may circulate among the SARI population and vary with climatic and demographic characteristics. This finding highlights the importance of sustained sentinel surveillance of SARIs at the local and national levels, which can guide accurate evaluations of the prevalence of etiological agents of SARI and the burden of disease and, most importantly, shape public policies on SARI prevention and responses to SARI activity.

Supporting information

S1 File. Minimal data set.

S2 File. Sequences of primers targeting Flu A/B used in real-time RT-PCR.

Acknowledgments

We would like to express our gratitude to the physicians and nurses from Jinshan District Central Hospital for their significant contribution to the data collection and specimen collection.

Funding

This work was supported by the Research Project of Shanghai Municipal Health Commission (201940428) and the Infectious Disease and Epidemiology Project of the 6th Jinshan District Medical Key Specialty Construction (JSZK2019B05). The funder had no role in the study design, data collection and analysis, decision to publish or preparation of the manuscript.

Availability of data and materials

All relevant data are contained within the manuscript and its Supporting Information files.

Author Contributions

Conceptualization: Jian Li, Jian-Min Zhu

Data curation: Can-Lei Song, Tang Wang, Shu-Hua Li

Formal analysis: Jian Li, Can-Lei Song, Tang Wang

Funding acquisition: Can-Lei Song, Shu-Hua Li

Investigation: Can-Lei Song, Tang Wang, Shu-Hua Li

Methodology: Jian Li, Can-Lei Song, Yu-Long Ye , Jian-Ru Du

Project administration: Jian-Min Zhu

Supervision: Can-Lei Song, Tang Wang, Jian-Min Zhu

Validation: Jian Li, Can-Lei Song, Tang Wang, Yu-Long Ye, Jian-Ru Du, Shu-Hua Li, Jian-Min Zhu

Writing-original draft: Jian Li, Can-Lei Song, Tang Wang

Writing-review & editing: Jian Li, Can-Lei Song, Tang Wang, Yu-Long Ye, Jian-Ru Du, Shu-Hua Li, Jian-Min Zhu

Competing interests

The authors declare that they have no competing interests.

References

1. Chakhunashvili G, Wagner AL, Power LE, Janusz CB, Machablishvili A, Karseladze I, et al. Severe Acute Respiratory Infection (SARI) sentinel surveillance in the country of Georgia, 2015-2017. PLos One.2018;13(7):e0201497. https://doi.org/10.1371/journal.pone.0201497 PMID:30059540. PubMed Central PMCID: PMC6066249.

2. Elhakim MM, Kandil SK, Abd Elaziz KM, Anwar WA. Epidemiology of Severe Acute Respiratory Infection (SARI) cases at a sentinel site in Egypt, 2013-15. J Public Health (Oxf). 2020;42(3):525-33.https:// doi.org/ 10.1093/pubmed/fdz053 PMID: 31090911. PubMed Central PMCID: PMC7107553.

3. Williams B, Gouws E, Boschi-Pinto C, Bryce J, Dye C. Estimates of world-wide distribution of child deaths from acute respiratory infections. Lancet Infect Dis. 2002;2(1):25–32. https:// doi.org/10.1016/s1473-3099(01)00170-0 PMID: 11892493.

4. WHO. The global burden of disease: 2004 update. May 21th, 2020. Available from: http://www.who.int/healthinfo/global_burden_disease/GBD_report_2004update_full.pdf.

5. Watt JP, Wolfson LJ, O'Brien KL, Henkle E, Deloria-Knoll M, McCall N,et al. Burden of disease caused by Haemophilus influenzae type b in children younger than 5 years: global estimates. Lancet. 2009;374(9693): 903–11. https:// doi.org/10.1016/S0140-6736(09)61203-4 PMID: 19748399.

6. Subramoney K, Hellferscee O, Pretorius M, Tempia S, McMorrow M, von Gottberg A, et al. Human bocavirus, coronavirus,and polyomavirus detected among patients hospitalised with sev ere acute respiratory illness in South Africa, 2012 to 2013. Health Sci Rep. 2018;1(8):e59. https:// doi.org/10.1002/hsr2.59 PMID: 30623094. PubMed Central PMCID: PMC6266378.

7. Razanajatovo NH, Guillebaud J, Harimanana A, Rajatonirina S, Ratsima EH, Andrianirina ZZ, et al. Epidemiology of severe acute respiratory infections from hospital-based surveillance in Madagascar, November 2010 to July 2013. PLos One.2018;13(11):e0205124. https:// doi.org/10.1371/journal.pone.0205124 PMID: 30462659. PubMed Central PMCID: PMC6248916.

8. WHO. WHO global technical consultation: global standards and tools for influenza surveillance. May 21th, 2020. Available from: https://extranet.who.int/iris/restricted/bitstream/handle/10665/70724/WHO_HSE_GIP_2011.1_eng.pdf;jsessionid=53A32B0F697E2FEDAA77BF5F971ED037?sequence=1.

9. Byington CL, Enriquez FR, Hoff C, Tuohy R, Taggart EW, Hillyard DR, et al. Serious bacterial infections in febrile infants 1 to 90 days old with and without viral infections. Pediatrics. 2004;113(6):1662–6. https:// doi.org/10.1542/peds.113.6.1662 PMID: 15173488.

10. Zhao Y, Lu R, Shen J, Xie Z, Liu G, Tan W. Comparison of viral and epidemiological profiles of hospitalized children with severe acute respiratory infection in Beijing and Shanghai, China. BMC Infect Dis. 2019;19(1):729. https:// doi.org/10.1186/s12879-019-4385-5 PMID: 31429710. PubMed Central PMCID: PMC6701130.

11. Nguenha N, Tivane A, Pale M, Machalele L, Nacoto A, pPires G, et al. Clinical and epidemiological characterization of influenza virus infections in children with severe acute

respiratory infection in Maputo, Mozambique: Results from the implementation of sentinel surveillance,2014-2016. PLoS One.2018;13(3):e0194138. https:// doi.org/10.1371/journal.pone.0194138 PMID: 29590162. PubMed Central PMCID: PMC5874022.

12. Yu H, Huang J, Huai Y, Guan X, Klena J, Liu S, et al. The substantial hospitalization burden of influenza in central China: surveillance for severe, acute respiratory infection, and influenza viruses, 2010-2012.Influenza Other Respir Viruses. 2014; 8(1):53-65. https:// doi.org/10.1111/irv.12205 PMID: 24209711. PubMed Central PMCID: PMC4177798.

13. Fica A, Sotomayor V, Fasce R, Dabanch J, Soto A, Charpentier P, et al. Severe acute respiratory infections (SARI) from influenza in adult patients in Chile: the experience of a sentinel hospital. Rev Panam Salud Publica. 2019;43:e1. https://doi.org/10.26633/RPSP.2019.1 PMID:31093225. PubMed Central PMCID: PMC6393720.

14. Foy HM, Cooney MK, McMahan R, et al. Viral and mycoplasmal pneumonia in a prepaid medical care group during an eight-year period. Am J Epidemiol. 1973; 97: 93-102. https://doi.org/10.1093/oxfordjournals.aje.a121493 PMID: 4347511.

15. Waites KB, Xiao L, Liu Y, Balish MF, Atkinson TP. Mycoplasma pneumoniae from the respiratory tract and beyond. Clin Microbiol Rev.2017; 30: 747-809. https://doi.org/10.1128/CMR.00114-16 PMID: 28539503. PubMed Central PMCID: PMC5475226.

16. Bureau of statistics of Jinshan district. Jinshan statistical yearbook 2018. May 20th, 2020. Available from: http://www.jinshan.gov.cn/Attach/Attaches/201911/201911140517237545.pdf.

17. WHO. Manual for the laboratory diagnosis and virological surveillance of influenza. May 21th,

2020. Available from:http://whqlibdoc.who.int/publications/2011/9789241548090_eng.pdf.

18. Meerhoff TJ, Simaku A, Ulqinaku D, Torosyan L, Gribkova N, Shimanovich V, et al. Surveillance for severe acute respiratory infections (SARI) in hospitals in the WHO European region—an exploratory analysis of risk factors for a severe outcome in influenza-positive SARI cases. BMC Infect Dis. 2015; 15: 1.https://doi.org/10.1186/s12879-014-0722-x PMID: 25567701. PubMed Central PMCID: PMC4314771.

19. Huo X, Qin Y, Qi X, Zu R, Tang F, Li L, et al. Surveillance of 16 respiratory viruses in patients with influenza-like illness in Nanjing, China. J Med Virol. 2012; 84(12):1980-4. https://doi.org/ 10.1002/jmv.23401 PMID: 23080506. PubMed Central PMCID: PMC7166984.

20. Breiman RF, Cosmas L, Njenga M, Williamson J, Mott JA, Katz MA, et al. Severe acute respiratory infection in children in a densely populated urban slum in Kenya, 2007–2011. BMC Infect Dis. 2015; 15: 95.https://doi.org/10.1186/s12879-015-0827-x PMID: 25879805. PubMed Central PMCID: PMC4351931.

21.Butsashvili M, Kandelaki G, Zakhashvili K, Machablishvili A, Avaliani N. Influenza-associated mortality in georgia (2009-2011). Influenza Res Treat. 2012;2012:480763. https://doi.org/10.1155/2012/480763 PMID: 23074667.PubMed Central PMCID: PMC3447290.

22. Xu W, Guo L, Dong X, Li X, Zhou P, Ni Q, et al. Detection of viruses and mycoplasma pneumonia in hospitalized patients with severe acute respiratory infection in northern China, 2015-2016. Jpn J Infec Dis. 2018; 71(2):134-139. https://doi.org/10.7883/yoken.JJID.2017.412 PMID: 29491245.

23. Costa C, Bergallo M, Astegiano S,Sidoti F, Terlizzi ME, Gambarino S, et al. Detection of human rhinoviruses in the lower respiratory tract of lung transplant recipients. Arch Virol 2011;156(8):1439-43. https://doi.org/10.1007/s00705-011-0986-z PMID: 21461933. PubMed Central PMCID: PMC7087099.

24. Hicks LA, Shepard CW, Britz PH, Erdman DD, Fischer M, Flannery BL, et al. Two outbreaks of severe respiratory disease in nursing homes associated with rhinovirus. J Am Geriatr Soc 2006; 54(2):284-9. https://doi.org/10.1111/j.1532-5415.2005.00529.x PMID: 16460380.

25. Mikulska M, Del Bono V, Gandolfo N, Dini S, Dominietto A, Di Grazia C, et al. Epidemiology of viral respiratory tract infections in an outpatient haematology facility. Ann Hematol 2014;93(4):669-76. https://doi.org/10.1007/s00277-013-1912-0 PMID: 24097084. PubMed Central PMCID: PMC7079995.

26. Louie JK, Yagi S, Nelson FA, Kiang D, Glaser CA, Rosenberg J, et al. Rhinovirus outbreak in a long term care facility for elderly persons associated with unusually high mortality. Clin Infect Dis 2005;41(2):262-5. https://doi.org/10.1086/430915 PMID: 15983926. PubMed Central PMCID: PMC7107978.

27. Wald TG, Shult P, Krause P, Miller BA, Drinka P, Gravenstein S. A rhinovirus outbreak among residents of a long-term care facility. Ann Intern Med 1995;123(8):588-93. https://doi.org/10.7326/0003-4819-123-8-199510150-00004 PMID: 7677299.

28. Wang KQ, Xi W, Yang DH, Zheng YL, Zhang YQ, Chen YS, et al. Rhinovirus is associated with severe adult community-acquired pneumonia in China. J Thorac Dis; 2017;9(11):4502-4511. https://doi.org/10.21037/jtd.2017.10.107 PMID: 29268520. PubMed Central PMCID: PMC5721016.

29. Lui G, Ip M, Lee N, Rainer TH, Man SY, Cockram CS, et al. Role of ‘atypical pathogens’ among adult hospitalized patients with community-acquired pneumonia. Respirology.2009; 14(8):1098–1105. https://doi.org/10.1111/j.1440-1843.2009.01637.x PMID: 19818051.

30. Goktas S, Sirin MC. Prevalence and seasonal distribution of respiratory viruses during the 2014 – 2015 season in Istanbul. Jundishapur J Microbiol. 2016; 9(9): e39132. https://doi.org/10.5812/jjm.39132 PMID: 27800148. PubMed Central PMCID: PMC5086027.

31. Azziz Baumgartner E, Dao CN, Nasreen S, Bhuiyan MU, Mah-E-Muneer S, Ai Mamun A. et al. Seasonality, timing, and climate drivers of influenza activity worldwide. J Infect Dis. 2012; 206(6):838-46. https://doi.org/10.1093/infdis/jis467 PMID: 22829641.

32. Zhang Y, Muscatello DJ, Wang Q, Yang P, Pan Y, Huo D, et al. Hospitalizations for influenza-associated severe acute respiratory infection, Beijing, China, 2014-2016. Emerg Infect Dis. 2018;24(11):2098-2102.https://doi.org/10.3201/eid2411.171410 PMID: 30334719. PubMed Central PMCID: PMC6199980.

33. Thompson WW, Shay DK, Weintraub E, Brammer L, Bridges CB, Cox NJ, et al. Influenza-associated hospitalizations in the United States. JAMA. 2004; 292(11):1333–1340. https://doi.org/10.1001/jama.292.11.1333 PMID: 15367555.

34. Hasman H, Pachucki CT, Unal A, Nguyen D, Devlin T, Peeples ME, et al. Aetiology of influenza-like illness in adults includes parainfluenzavirus type 4. J Med Microbiol. 2009;58 (Pt4):408–413. https://doi.org/10.1099/jmm.0.006098-0 PMID: 19273634. PubMed Central PMCID: PMC2778239.

Table 1. Demographic characteristics of adult SARI patients in a surveillance hospital in Jinshan, Shanghai, April 2017 to March 2018

Characteristics	SARI patients			*P* value*
	All (%) [n=397]	With confirmed pathogens (%) [n=250]	Without confirmed pathogens (%) [n=147]	
Sex				0.315
Male	194(48.9)	127(50.8)	67(45.6)	
Female	203(51.1)	123(49.2)	80(54.4)	
Age group (median, years)	68.0	67.0	69.0	0.357
<30	25(6.3)	17(6.8)	8(5.4)	0.786
30-39	19(4.8)	10(4.0)	9(6.1)	
40-59	58(14.6)	39(15.6)	19(12.9)	
60-79	207(52.1)	128(51.2)	79(53.7)	
≥80	88(22.2)	56(22.4)	32(21.9)	
BMI				0.657
<20	118(29.7)	73(29.2)	45(30.6)	
20-25	208(52.4)	135(54.0)	73(49/7)	
>25	71(17.9)	42(16.8)	29(19.7)	
Chronic medical conditions				
At least one	278(70.0)	178(71.2)	100(68.0)	0.505
Asthma	12(3.0)	6(2.4)	6(4.1)	0.345
Chronic bronchitis	49(12.3)	30(12.0)	19(12.9)	0.787
COPD	28(7.1)	13(5.2)	15(10.2)	0.060
Hypertension	152(38.3)	95(38.0)	57(38.8)	0.878
Cardiovascular disease	30(7.6)	22(8.8)	8(5.4)	0.222
Diabetes	61(15.4)	38(15.2)	23(15.6)	0.905
Cerebrovascular disorder	20(5.0)	14(5.6)	6(4.1)	0.504

Tumor	19(4.8)	14(5.6)	5(3.4)	0.322

**P* values denote comparisons between SARI patients with confirmed pathogens and SARI patients without confirmed pathogens.

Table 2. Etiological agent distributions among adult SARI patients in a surveillance hospital in Jinshan, Shanghai, April 2017 to March 2018

Etiological agent	Frequency# (n)	Percent of samples* (%)
Influenza virus A		
pH1N1	16	4.0
H3N2	44	11.1
Influenza virus B		
Yamagata	25	6.3
Victoria	2	0.5
Parainfluenza virus		
Type 1	8	2.0
Type 2	0	0
Type 3	6	1.5
Type 4	5	1.3
Human coronavirus		
Type 229E	6	1.5
Type OC43	4	1.0
Type HKU1	6	1.5
Type NL63	8	2.0
Respiratory syncytial virus		
Type A	0	0
Type B	2	0.5
Human rhinovirus	32	8.1
Adenovirus	46	11.6

Human metapneumovirus	6	1.5
Human bocavirus	1	0.3
Mycoplasma pneumoniae	95	23.9
Single infection	198	49.9
Multiple infection		
2 pathogens	43	10.8
3 pathogens	8	2.0
4 pathogens	1	0.3

[#]The frequency of each pathogen may include both the samples with single infection and those with multiple infection, and their total number is larger than the sum of samples with single infection and multiple infection. *Percent of samples is the frequency of samples with a positive etiology divided by the total enrolled samples (397 cases).

Table 3. Clinical and epidemi[illegible]c characteristics of adult SARI patients in a surveillance hospital in Jinshan, Shanghai, April 2017 to March 2018

Characteristics	SARI patients			*P* value*
	All (%) [n=397]	With confirmed pathogens (%) [n=250]	Without confirmed pathogens (%) [n=147]	
Temperature ≥39°C	189(47.6)	126(50.4)	63(42.9)	0.176

Cough	394(99.2)	249(99.6)	145(98.6)	0.558
Sputum production	351(88.4)	219(87.6)	132(89.8)	0.509
Pharyngalgia	27(6.8)	18(7.2)	9(6.1)	0.680
Thoracalgia	28(7.1)	19(7.6)	9(6.1)	0.687
Dyspnea	19(4.8)	11(4.4)	8(5.4)	0.808
Runny nose	11(2.8)	7(2.8)	4(2.7)	1.000
Vomiting	15(3.8)	10(4.0)	5(3.4)	0.795
Acceptance of chest radiographic exam	382(96.2)	236(94.4)	146(99.3)	**0.013**
Presence of radiographic diagnosis of pneumonia	258/382(67.5)	153/236(64.8)	105/146(71.9)	0.349
Visited a live poultry market	3(0.8)	3(1.2)	0(0)	0.299
Contact with live poultry	30(7.6)	19(7.6)	11(7.5)	1.000
Contact with patient with fever	32(8.1)	24(9.6)	8(5.4)	0.182
Smoking				0.860
Current	43(10.8)	28(11.2)	15(10.2)	
Former	66(16.6)	43(17.2)	23(15.6)	
Never	288(72.6)	179(71.6)	109(74.2)	
Vaccinated with pneumococcal conjugate vaccine	5(1.3)	3(1.2)	2(1.4)	1.000
Vaccinated with influenza vaccine	1(0.3)	1(0.4)	0(0)	1.000

**P* values denote comparisons between SARI patients with confirmed pathogens and SARI patients without confirmed pathogens.

Table 4. Comparison of characteristics of SARI patients infected with only one of the 6 main pathogens in a surveillance hospital in Jinshan, Shanghai, April 2017 to March 2018

Characteristics	*M. pneumoniae* (%) [n=51]	AdV (%) [n=31]	HRhV (%) [n=19]	Flu A/H3N2 (%) [n=35]	Flu B/Yamagata (%) [n=21]	Flu A /pH1N1 (%) [n=16]	*P* value*
Sex							0.750
Male	28(54.9)	19(61.3)	8(42.1)	18(51.4)	9(42.9)	8(50.0)	
Female	23(45.1)	12(38.7)	11(57.9)	17(48.6)	12(57.1)	8(50.0)	
Age group(years)							0.247
<30	5(9.8)	3(9.7)	1(5.3)	1(2.9)	0(0)	2(12.5)	
30-39	3(5.9)	3(9.7)	0(0)	0(0)	1(4.8)	1(6.3)	
40-59	12(23.5)	1(3.2)	2(10.5)	5(14.3)	3(14.3)	2(12.5)	
60-79	20(39.2)	15(48.4)	8(42.1)	24(68.6)	12(57.1)	9(56.3)	
≥80	11(21.6)	9(29.0)	8(42.1)	5(14.3)	5(23.8)	2(12.5)	
At least one comorbidity	25(49.0)	23(74.2)	13(68.4)	26(74.3)	18(85.7)	11(68.8)	**0.034**
Temperature ≥39°C	30(58.8)	16(51.6)	6(31.6)	16(45.7)	9(42.9)	8(50.0)	0.444
Cough	51(100)	31(100)	19(100)	34(97.1)	21(100)	16(100)	0.705
Sputum production	39(76.5)	29(93.5)	15(78.9)	30(85.7)	19(90.5)	16(100)	0.120
Pharyngalgia	3(5.9)	3(9.7)	2(10.5)	2(5.7)	2(9.5)	2(12.5)	0.876
Thoracalgia	4(7.8)	2(6.5)	1(5.3)	0(0)	2(9.5)	1(6.3)	0.523
Dyspnea	0(0) †	1(3.2)	4(21.1) †	1(2.9)	1(4.8)	0(0)	**0.007**
Runny nose	1(2.0)	1(3.2)	1(5.3)	1(2.9)	0(0)	2(12.5)	0.360
Vomiting	0(0)	3(9.7)	0(0)	3(8.6)	1(4.8)	1(6.3)	0.123
Presence of radiographic diagnosis of pneumonia	38(74.5)#	17(54.8)	13(68.4)	15(42.9) #	13(61.9)	7(43.8)	**0.042**
Visited a live poultry market	1(2.0)	1(3.2)	0(0)	0(0)	0(0)	0(0)	0.880
Contact with live poultry	6(11.8)	3(9.7)	2(10.5)	1(2.9)	1(4.8)	1(6.3)	0.753
Contact with a patient with fever	3(5.9)	4(12.9)	2(10.5)	1(2.9)	3(14.3)	2(12.5)	0.442
Current Smoker	2(3.9)	4(12.9)	2(10.5)	6(17.1)	3(14.3)	3(18.8)	0.333
Former Smoker	10(19.6)	7(22.6)	2(10.5)	7(20.0)	3(14.3)	0(0)	

Never Smoked	39(76.5)	20(64.5)	15(78.9)	22(62.9)	15(71.4)	13(81.3)	

**P* values denote comparisons among the six main pathogens. † and # signify *P*<0.05 for pairwise comparisons. † refers to comparisons between the single-infected SARI patients with *M. pneumoniae* and those with HRhV. # refers to comparisons between SARI patients infected with *M. pneumoniae* and those infected with Flu A/H3N2.

Table 5. Treatments and prognoses in adult SARI patients in a surveillance hospital in Jinshan, Shanghai, April 2017 to March 2018

Characteristics	SARI patients			*P* value*
	All (%) [n=397]	With confirmed pathogens (%) [n=250]	Without confirmed pathogens (%) [n=147]	
Clinical course (median, days)				
From illness onset to admission	3	3	3	0.567
Length of hospitalization	10	10	10	0.545
Antibiotics prior to hospitalization	241（61.0）	151（60.9）	90（61.2）	0.723
Antibiotics during hospitalization	393(99.0)	246(98.4)	147(100)	0.301
Antivirals	11(2.8)	7(2.8)	4(2.7)	1.000
Glucocorticoids	112(28.2)	72(27.2)	40(28.8)	0.734
Oxygen therapy	196(49.4)	124(49.6)	72(49.0)	0.918
Complications	61(15.4)	37(14.8)	24(16.3)	0.684
Death	3(0.8)	2(0.8)	1(0.7)	1.000

**P* values denote comparisons between SARI patients with confirmed pathogens and SARI patients without confirmed pathogens.

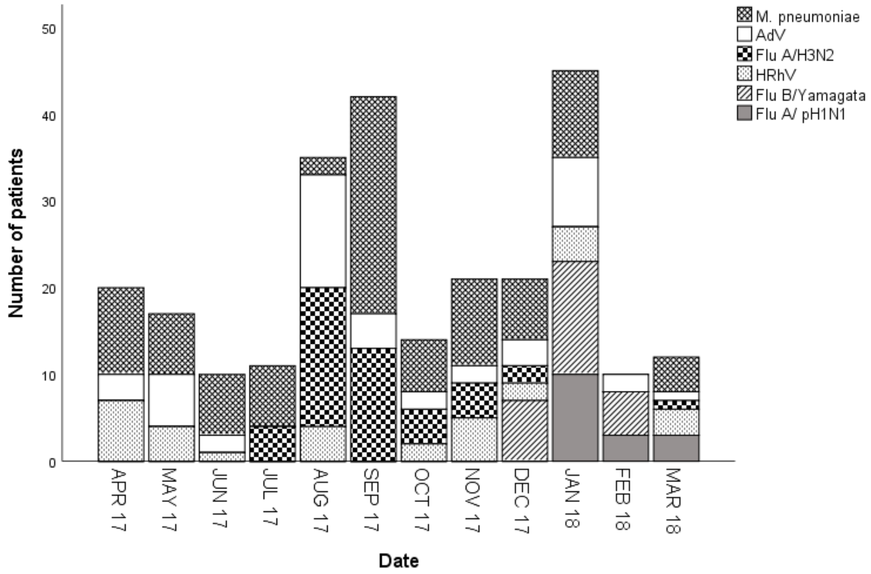

Fig. 1 Monthly variations in the six main pathogens detected in adult SARI patients in a surveillance hospital in Jinshan, Shanghai, April 2017 to March 2018

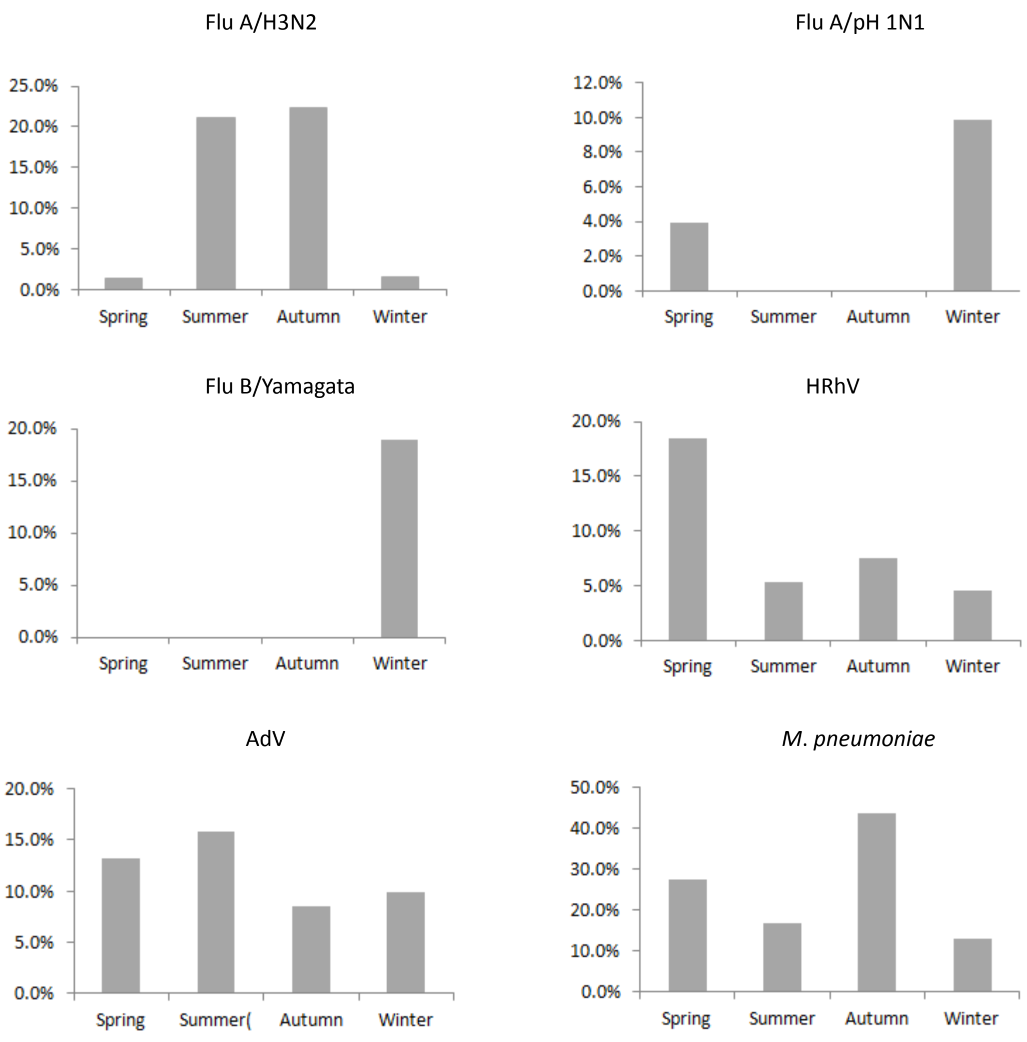

Fig. 2 Detection rates of the six main pathogens in adult SARI patients in different seasons in a surveillance hospital in Jinshan, Shanghai. Each panel shows the seasonal distribution of a pathogen in SARI patients. For each pathogen, the detection rate on the y-axis refers to the number of positive patients divided by the total number of patients tested in a season.

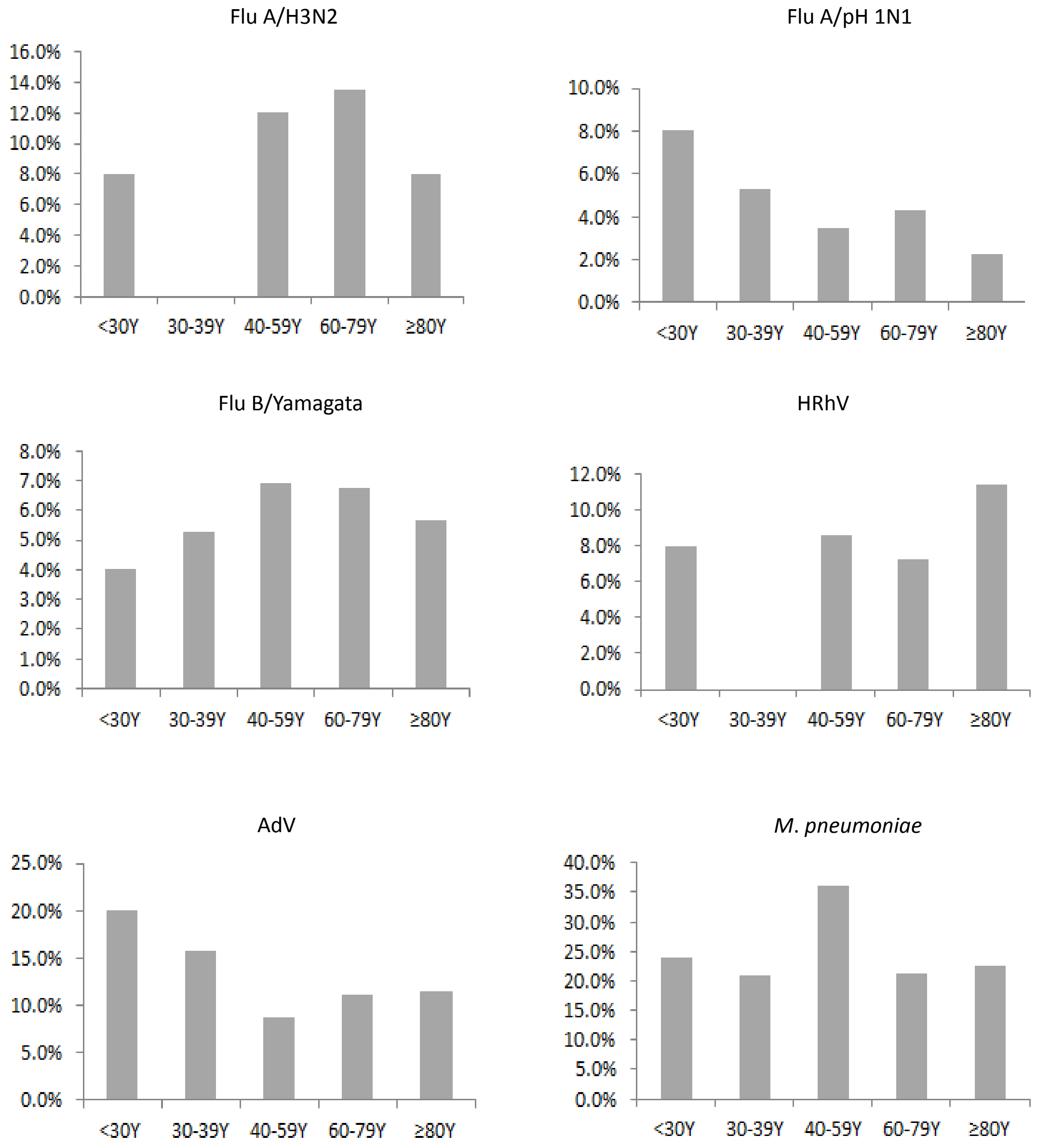

Fig. 3 Detection rates of the six main pathogens in SARI patients according to age group in a surveillance hospital in Jinshan, Shanghai, April 2017 to March 2018. Each panel shows the age group-specific detection rate of one pathogen in SARI patients. For each pathogen, the detection rate on the y-axis refers to the number of positive patients divided by the total number of patients tested in each age group.

Revised Figure with Track Changes
Click here to access/download;Figure;Revised Figure with Track Changes.docx

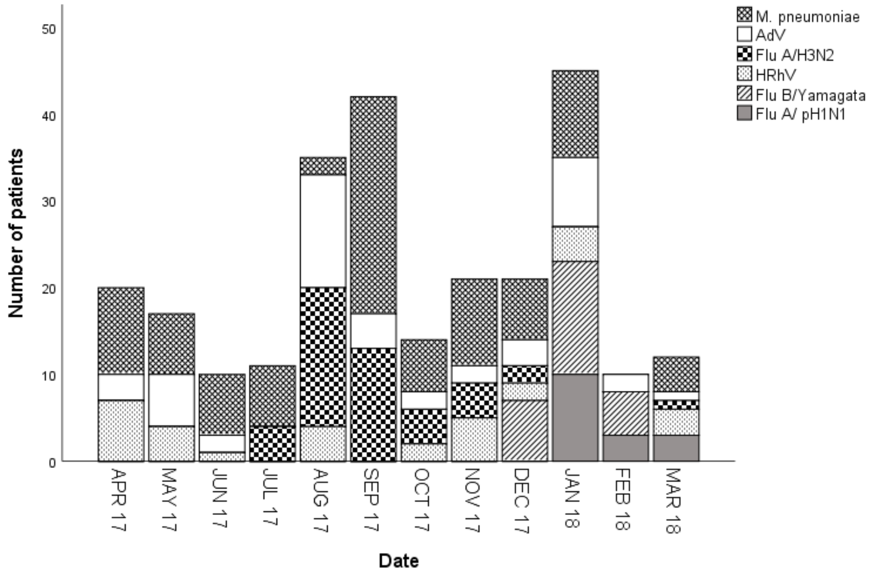

Fig. 1 — Monthly variations ~~of~~ in the six main pathogens detected ~~among~~ in adult SARI patients in a surveillance hospital in Jinshan, Shanghai, April 2017 to March 2018

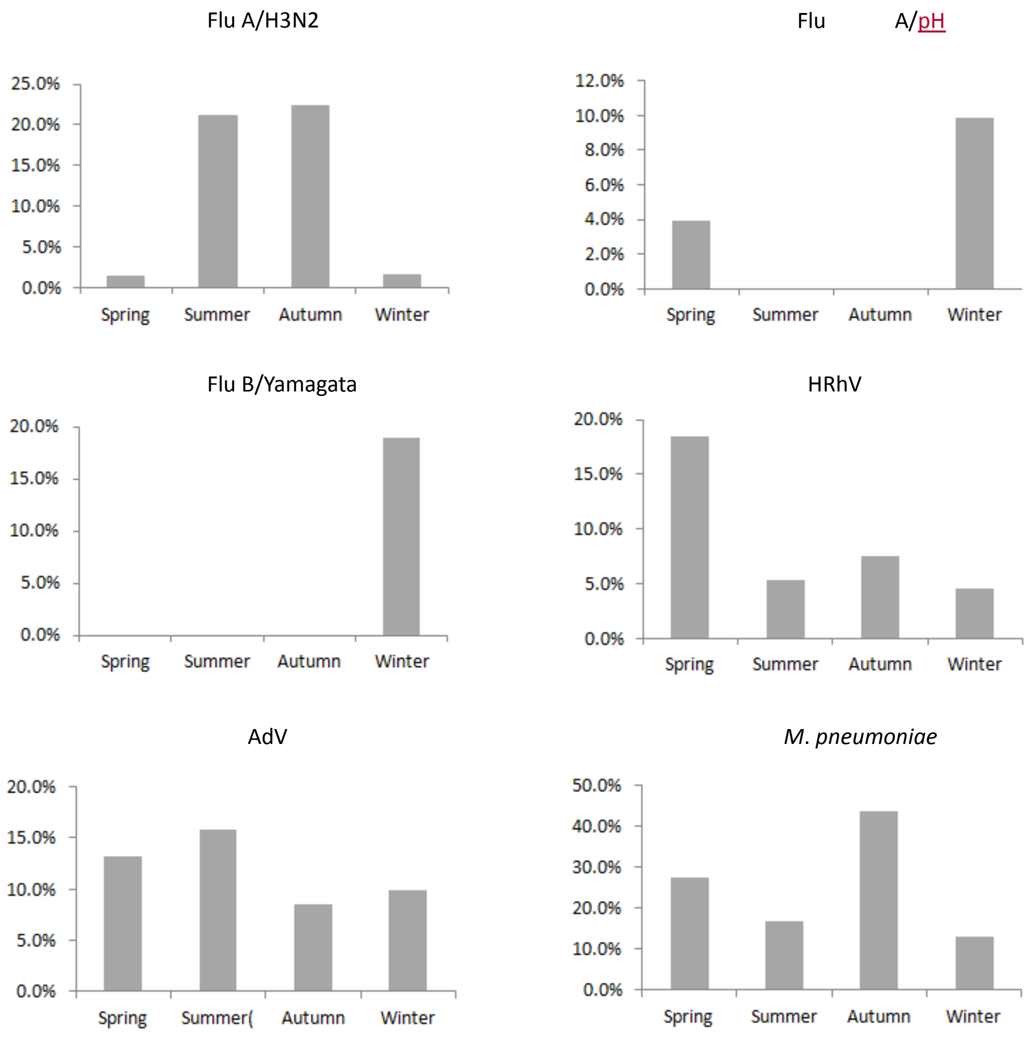

Fig. 2_—Detection rates of the six main pathogens among in adult SARI patients in different seasons in a surveillance hospital in Jinshan, Shanghai. Each panel showsed the seasonal detection distribution of one a pathogen from in SARI patients. For each pathogen, the detection rate at on the y-axis refersred to the number of positive patients divided by the total number of

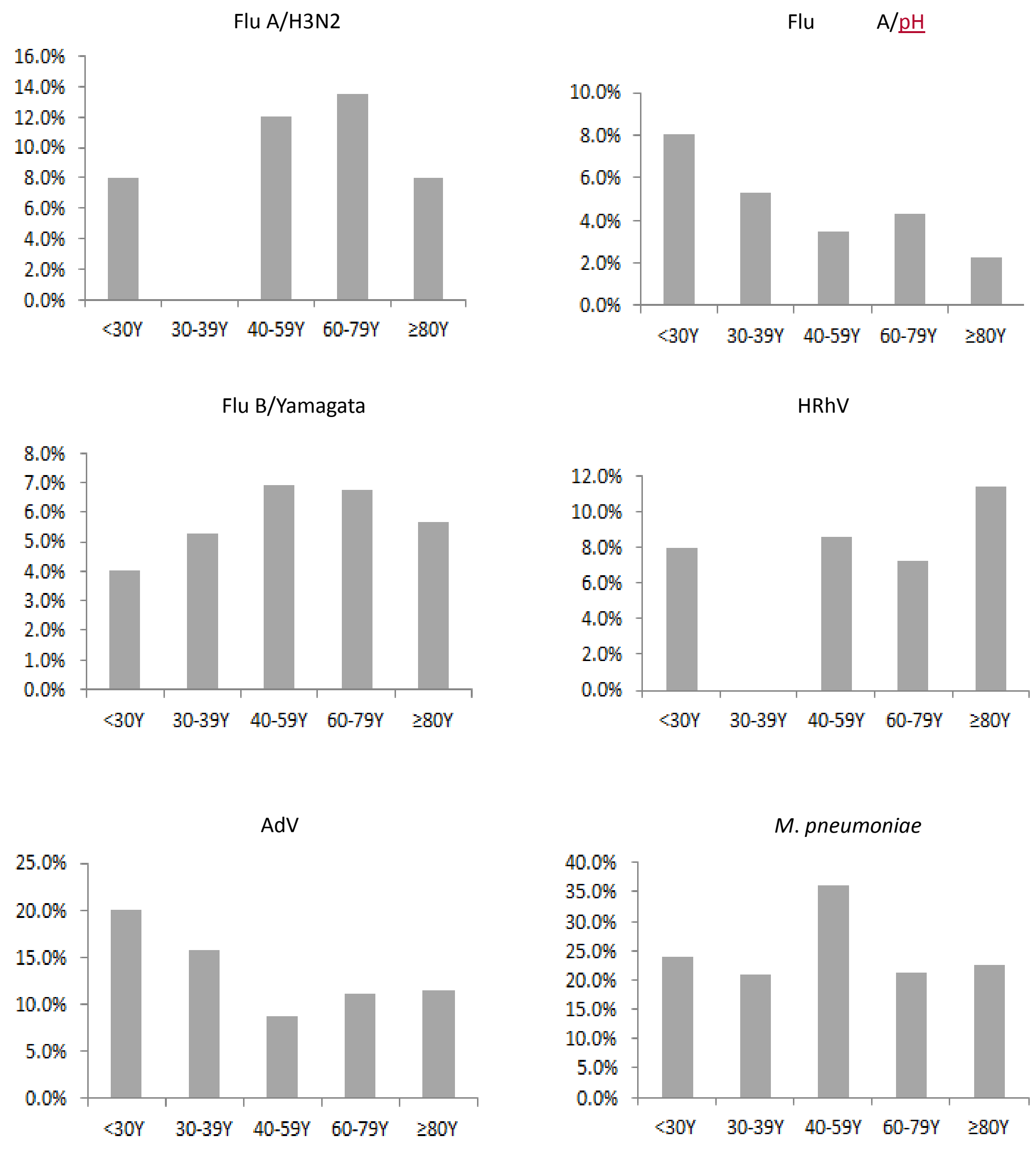

Fig. 3 –Detection rates of the six main pathogens ~~among~~ in SARI patients according to age groups in a surveillance hospital in Jinshan, Shanghai, April 2017 to March 2018. Each panel shows~~ed~~ the age -group-specific detection rate of one pathogen ~~from~~ in SARI patients. For each pathogen, the detection rate ~~at~~ on the y-axis refer~~r~~s~~ed~~ to the number of positive patients divided by the total number of patients tested in ~~an~~ each age group.

Click here to access/download
Supporting Information
STROBE_checklist_v4_combined_PlosMedicine.docx

Minimal data set

Click here to access/download
Supporting Information
Minimal data set.xlsx

Click here to access/download
Supporting Information
Sequences of primers targeting Flu AB.doc

Etiological and Epidemiological ~~Characters~~Characteristics of Severe Acute Respiratory Infection Caused by Multiple Viruses and *Mycoplasma Pneumoniae* in Adult Patients in Jinshan, ~~of~~ Shanghai: A Pilot Hospital-based Surveillance Study

Jian Li [1†], Can-Lei Song [2†], Tang Wang [2†], Yu-Long Ye [3], Jian-Ru Du [3], Shu-Hua Li [2], Jian-Min Zhu[2]

[†] These authors contributed equally to this work.~~Contributed equally~~

[1]*Clinical Research Center, Ruijin Hospital, Shanghai Jiao Tong University School of Medicine, Shanghai, China*

[2] *Department of Acute Infectious Diseases Control, Jinshan District Center for Diseases Control and Prevention, Shanghai, China*

[3]*Department of Microbiology, Jinshan District Center for Diseases Control and Prevention, Shanghai, China*

Email addresses:

Jian Li : nclijian@163.com

Can-Lei Song: 629309340@163.com

Tang Wang: 1090595528@qq.com

Yu-Long Ye: yeyulong99@163.com

Jian-Ru Du: 646947222@qq.com

Shu-Hua Li: hsli167@163.com

Jian-Min Zhu: zhujm12@163.com

Corresponding author:

Dr. Jian Li: Clinical Research Center, Ruijin Hospital, Shanghai Jiao Tong University School of

Medicine, 197 Ruijin Er Rd, Huangpu District, Shanghai, China, 200025

Dr. Jian-Min Zhu: Department of Acute Infectious Diseases Control, Jinshan District Center for Diseases Control and Prevention, 94 Weisheng Rd, Jinshan District, Shanghai, China, 201599

Abstract

Background

Severe acute respiratory infection (SARI) ~~presents~~ results in a tremendous disease burden worldwide. Available research on ~~the~~ active surveillance ~~of~~ among hospitalized adult patients ~~suffered~~suffering from SARI in China ~~was~~is limited. This pilot study aimed to identify associated etiologies and describe the demographic, epidemiological and clinical profiles of hospitalized SARI~~-associated~~ patients aged over 16 years ~~old~~ in Jinshan, Shanghai.

Methods

Active surveillance was conducted at 1 sentinel hospital in Jinshan district, Shanghai, from April 2017 to March 2018. Hospitalized SARI patients aged ~~over~~ more than 16 years ~~old~~ were enrolled, ~~the~~and nasopharyngeal swabs were collected within 24 hours of admission and tested for multiple respiratory viruses (including 18 common viruses) and *Mycoplasma pneumoniae* (*M. pneumoniae*) with real-time polymerase chain reaction. Demographic, epidemiological and clinical information ~~were~~was obtained from case report forms.

Results

~~Of~~ In total, 397 SARI patients were enrolled~~;~~, the median age was 68 years, and 194 (48.9%) patients were male**.** A total of 278 (70.0%) patients had at least one underlying chronic medical ~~conditions~~condition**.** The most frequent ~~symptom was~~symptoms were cough (99.2%) and sputum

production (88.4%). The median duration of hospitalization was 10 days. A total of 250 infection patients (63.0%) were positive ~~identified as~~for ~~having~~ at least one ~~positive~~ pathogen ~~infection~~, of whom 198 (49.9%) ~~were~~~~had~~were positive for a single ~~infection~~ pathogen and 52 (13.1%) ~~were~~~~had~~were positive for multiple ~~infections~~pathogens. The pathogens identified most frequently were *M. pneumoniae* (23.9%, 95/397), followed by adenovirus (AdV) (11.6%, 46/397), influenza virus A/H3N2 (Flu A/H3N2) (11.1%, 44/397), human rhinovirus (HRhV) (8.1%, 32/397), influenza virus B/Yamagata (Flu B/Yamagata) (6.3%, 25/397), pandemic influenza virus A/H1N1 (Flu A~~–~~/pH1N1) (4.0%, 16/397), parainfluenza virus (PIV) t~~T~~ype 1 (2.0%, 8/397), human coronavirus (HCoV) t~~T~~ype NL63 (2.0%, 8/397), HCoV t~~T~~ype 229E (1.5%, 6/397), HCoV ~~T~~type HKU1 (1.5%, 6/397), PIV t~~T~~ype 3 (1.5%, 6/397), human metapneumovirus (HMPV) (1.5%, 6/397), PIV t~~T~~ype 4 (1.3%, 5/397), HCoV t~~T~~ype OC43 (1.0%, 4/397), influenza virus B/Victoria (Flu B/Victoria) (0.5%, 2/397), respiratory syncytial virus (RSV) ~~T~~type B (0.5%, 2/397), and human bocavirus (HBoV) (0.3%, 1/397). The seasonality of pathogen-confirmed SARI patients ~~conformed~~ had~~to~~ a bimodal ~~shape~~distribution, with the first peak in summer and the second peak in winter. ~~The statistically~~Statistically significant differences were observed with respect to the ~~proportion~~rates of ~~s of~~ dyspnea, radiographically diagnosed~~is of~~ pneumonia and the presence of at least one comorbidity ~~among~~ in patients who were ~~were single-~~infected ~~by~~with only *M. pneumoniae*, AdV, HRhV, Flu A/H3N2, Flu A /pH1N1 ~~and~~ or Flu B/Yamagata. The differences ~~of~~in the positivitye rates of the above 6 ~~main~~ pathogens among the different age groups were no~~t~~n ~~statistically~~ significant.

Conclusions

M. pneumoniae, AdV and Flu A/H3N2 were the ~~leading~~ main pathogens detected ~~among~~ in

hospitalized SARI patients aged more than 16 years ~~old~~ in Jinshan district, Shanghai. Our ~~finding highlights~~findings highlight the importance of ~~sustaining~~ sustained ~~multi-pathogen~~multipathogen surveillance ~~of~~ among SARI patients in sentinel hospitals, which can ~~provides~~provide useful information on SARI etiologies~~y~~, epidemiology, and clinical characteristics.

Key words: Severe acute respiratory infection, sentinel surveillance, pathogen, epidemiology

Background

Severe acute respiratory infection (SARI) has been considered an important ~~cause~~ contributor to~~of~~ morbidity and mortality ~~inat~~in all age groups~~s worldwide~~, particularly ~~in~~ children, ~~the~~ elderly individuals and individuals with compromised immune, cardiac and pulmonary systems, worldwide [1-3]. It is estimated that SARI ~~caused~~causes approximately 4.2 million ~~of~~ deaths annually. Of these, up to 90% are believed to occur in developing countries [4]. Various viral and bacterial pathogens are associated with SARI. Due to their extremely high potential ~~to~~for human-to-human transmission, these pathogens pose a substantial risk to human health. While bacterial infection~~s~~ ~~exert~~ has a ~~critical~~ substantial influence on ~~causing~~ the development of severe pneumonia [5], a significant proportion of ~~SARI~~SARIs are attributed to viral ~~infections~~ pathogens, such as influenza viruses A and B (Flu A/B), parainfluenza viruses (PIV), adenoviruses (AdVs), respiratory syncytial viruses (RSVs), human coronaviruses (HCoVs) and human rhinoviruses (HRhVs) [6]. Nevertheless, owing to the lack of gold standard diagnostic methods to ~~swiftly~~ rapidly identify~~determine~~ etiological agents, most ~~of the~~ patients ~~may be~~are treated with antibiotics empirically [7]. Rapid etiologic diagnosis therefore remains a significant public health challenge.

Routine pathogen monitoring is critical for preparedness for and response to the SARI epidemic

~~of SARI~~. Since SARI is the leading cause of hospitalization in children under the age of 5 years and of febrile episodes in infants younger than 3 months old, most available studies regarding the burden of SARI focus on ~~the~~ viral~~ruses~~ infections ~~of~~in children [8-11]. A ~~study of~~ SARI surveillance study in China revealed that 90% of patients were aged <15 years [12]. ~~Besides,~~ In addition, the majority of the data on the epidemiology of the etiologic agents of SARI ~~come~~ was collected in~~from more~~ developed regions. The ~~E~~epidemiological ~~characterizations~~characteristics and distributions of the major viral ~~agents~~ pathogens in adult SARI patients are still limited in developing regions [13]. *Mycoplasma pneumoniae* (*M. pneumoniae*) has long been considered an important etiology of respiratory disease~~,~~ and is more frequently isolated among children and young adults [14, 15]. ~~Limited~~ R~~r~~esearch ~~is available~~ on ~~the~~ active surveillance ~~of~~ in hospitalized adult patients ~~suffered~~suffering from SARI in China is scarce. ~~In response~~Accordingly, a pilot~~ing,~~ study on active surveillance ~~system for~~of SARI ~~had been~~was initiated to ~~address~~ characterize ~~the~~ community~~-~~-acquired pulmonary infections and ~~conduct epidemiologic and etiologic~~ monitor the~~ing~~ epidemiologic and etiologic characteristics of SARI caused by various viral pathogens and *M. pneumoniae* in adult inpatients in Jinshan district, Shanghai~~ since~~, in April 2017. The aim of the present study ~~is~~was to characterize the demographic~~y~~ and epidemiologic characteristics~~y~~ of SARI, ~~to~~ identify the etiologies and ~~to~~ assess the clinical profiles ~~of~~ associated with SARIs in hospitalized adult patients in Jinshan, Shanghai, ~~during~~ by performing 12 months of active surveillance.

Materials and methods

Study setting

Jinshan district is a suburb ~~and~~ located in southwest Shanghai, P.R. China. Active ~~S~~surveillance

was ~~piloted~~ initiated at Jinshan ~~district central hospital since~~District Central Hospital in April 2017 and ~~lasted~~ was conducted for 12 months. This hospital was selected ~~as~~because it is one of the largest general hospitals in the district and ~~also~~ ~~the~~ a national surveillance sentinel site for influenza virus. It serves most of the population of Jinshan district, ~~–~~with a total of 636 beds. In 2017, the registered population in Jinshan district was 523,641, of which 467,320 (89.24%) were adults aged more than 18 years [16].

Study subjects

All patients aged over 16 years ~~old~~ who were admitted to the intensive care unit, respiratory medicine department and general wards in the ~~sentinel~~ hospital were screened by a trained physician between April 2017 and March 2018. Patients were ~~defined~~ diagnosed~~as~~ with SARI ~~case~~~~cases~~ according to the World Health Organization (WHO) definition, which includes ~~if they have~~~~had~~ acute respiratory infection with a measured fever of ~~≥38°C~~≥38°C, cough onset within the last 10 days and ~~require~~required hospitalization [1].

Data collection

After hospital admission, a standard case report form was completed for each eligible patient. The form comprised information on demographic characteristics (sex, age, weight, height, residence), vaccination (received an~~ing~~ influenza vaccine 1 year before illness onset, ~~and~~ ever received a pneumococcal conjugate vaccine), admission~~tting~~ diagnosis, comorbidities (asthma, chronic bronchitis, chronic obstructive pulmonary disease (COPD), hypertension, diabetes, cardiovascular disease, tumor), clinical presentation (fever, cough, difficult breathing, sore throat), antibiotic treatments prior to hospitalization, exposure history (smoking, visiting a live poultry market, contact with live poultry, contact with a patient with fever and respiratory symptoms ~~during~~ within

2 weeks before illness onset). At discharge, the form was updated to include information ~~on~~ about treatment ~~accepted~~ in the hospital, chest computed tomographic (CT) ~~scans~~findings, complications and prognosis. Data were collected by the trained physician. To ensure the accuracy of the data, spouses or caregivers who lived with the patients for more than 2 weeks before illness onset were interviewed, and the medical records of the patients were reviewed~~ as well~~. Two radiologists interpreted chest CT scans independently. ~~When~~ In the case of a disagreement~~ arose~~, a third radiologist was consulted to reach a final decision. All the information that could identify the personality of patients was masked during or after data collection.

Specimen collection and laboratory testing

A single flocked polyester nasopharyngeal swab (Becton Dickinson, USA, MD) sample was collected from each SARI patient by a nurse within 24 hours of admission following a standard procedure. The swab was inserted into a cryovial containing 3 ~~3ml~~ml of viral transport medium (Tiandz, China, Beijing). The specimens were stored at ~~4˚C~~4°C in the hospital and transferred within 24 hours of collection to the laboratory at Jinshan ~~district center for disease control and prevention~~District Center for Disease Control and Prevention (CDC), where they were preserved at -~~70˚C~~70°C until test~~s~~ing was~~ were~~ performed. Viral RNA and DNA ~~A total of~~were extracted from 200~~-~~ μl samples ~~were adopted~~used~~ to extract viral RNA and DNA~~ using the QIAamp Viral RNA/DNA Mini Kit (Qiagen, Hilden, Germany) following the manufacturer's ~~instruction~~instructions. To guarantee integrity, specimens were lysed ~~at~~under denaturing conditions to deactivate RNases [1]. Pure viral RNA and DNA were eluted in 60 μl of low-salt buffer, and ~~, whereas~~ impurities were removed. Viral nucleic acid extracts were further processed by multiplex real-time reverse transcription polymerase chain reaction (RT-PCR). The qualitative

RespiFinder 2SMART multiplex real-time RT-PCR diagnostic strategy (Geneodx, Shanghai, China) was adopted to detect 15 respiratory pathogens, including PIV (types 1, 2, 3 and 4), HCoV (types 229E, OC43, HKU1 and NL63), RSV (types A and B), HRhV, AdV, human metapneumovirus (HMPV), human bocavirus (HBoV) and *M. pneumoniae*, using the CFX96™ r~~R~~eal-time PCR s~~S~~ystem (Bio-Rad, Hercules, CA, USA) according to the manufacturer's protocols. ~~Besides~~In addition, RNA from each specimen was identified for specific primers and probes that target Flu A/B using ~~another~~ real-time RT-PCR following the US CDC's protocol. Specimens ~~found~~ that were positive for Flu A and Flu B were subsequently ~~sub typed~~ subtyped for pandemic influenza virus A/H1N1 (Flu A~~-~~/pH1N1) and ~~,~~seasonal influenza virus A/H3N2 (Flu A/H3N2)~~,~~ ~~together with~~and Flu ~~B/ Yamagata~~ B/Yamagata and Flu ~~B/ Victoria~~B/Victoria, respectively [17]. These ~~testing~~tests were performed in ~~bio-safety~~the biosafety level 2 laboratory of the Jinshan CDC.

Statistics

The collected data were double-entered into a database constructed ~~by~~ in EpiData 3.1. Logic~~al~~ checks~~ing~~ ~~for~~ to assess the quality of data entry ~~was~~ were conducted. ~~The definition of s~~Single infection ~~referred~~ was defined as ~~to an~~ infection caused by one pathogen, and multiple infection~~s~~ was defined as ~~an~~ infection caused by at least 2 pathogens (virus/virus, virus/*M. pneumoniae*) in a single specimen. Continuous data ~~were~~are reported as ~~the~~ medians and ~~inter-quartile~~interquartile ranges (IQRs), and the Mann-Whitney U test was used to compare ~~the difference~~differences between groups. Categorical data ~~were~~are expressed as ~~frequency~~frequencies and proportions, and the ~~Chi~~chi-squared test or Fisher's exact test, as appropriate, ~~were~~was used to compare patients with and without confirmed ~~pathogen~~pathogens in terms of demographics, clinical characteristics,

epidemiologic characteristics, treatment and prognosis. Bonferroni's correction was used for pairwise ~~comparison~~comparisons. For proportions, the binomial 95% confidence~~-~~ interval ~~was~~ is reported. The analysis was performed using SPSS v. 25.0 (IBM Corporation, Armonk, NY, USA), and all tests were ~~performed~~ two-sided ~~at~~ with a~~the~~ 5% significance level.

Ethics statement

This study ~~belonged~~ was ~~to the~~ part of ~~the~~a hospital-based SARI surveillance program ~~of SARI of~~in Shanghai~~,~~ and was approved by the ethical review committee of the Shanghai Municipal Center for Disease Control and Prevention (Ref #: 2015-14). Written informed consent was obtained from patients or proxies before enrollment~~,~~ and from parents or guardians ~~for~~ of those under 18 years old. This study was conducted in accordance with the Declaration of Helsinki.

Results

Demographic characteristics

From April 2017 to March 2018, a total of 397 patients meeting the SARI case definition were admitted to our ~~sentinel site~~hospital~~,~~. ~~of whom o~~One or more pathogens were detected ~~from~~ in 250 patients (63.0%; 95% CI: 58.2-67.7%), and ~~the~~ negative results were ~~found~~ obtained from~~in~~ the ~~rest of~~ remaining 147 patients. The median age of the patients ~~were~~was 68 years (IQR: 59-78; range: 16 to 99 years). Among the SARI patients, 194 (48.9%) were male, and 203 (51.9%) were female. The ~~majorities~~majority of patients were ~~the elderly~~elderly patients aged ~~equal to or more than~~ 60 or more years (295 cases), ~~and~~ accounted for 74.3% of the total patients;~~,~~ 58 (14.6%) patients were 40-59 years of age, and 19 (4.8%) patients were 30-39 years of age. Those less than 30 years old represented only 6.3% of the total patients (25 cases). ~~The~~The~~A~~ percentages of patients with a body mass index (BMI) <20, between 20 and 25, and >25 ~~accounted for~~were

29.7%, 52.4% and 17.9%, respectively.~~–~~ A total of 278 SARI patients (70.0%) had at least one comorbidity, and 119 patients ~~suffered~~ had no comorbidity (Table 1). ~~The difference in proportion of gender~~~~differences in the proportions~~ There were no significant differences in ~~of~~ sex, age, BMI and underlying chronic medical conditions between SARI patients with confirmed ~~pathogen~~pathogens and those without confirmed ~~pathogen~~pathogens ~~did not show any statistical significance~~ (*P*>0.05).

Etiologies~~y~~

Of the 397 SARI patients, 198 (49.9%; 95% CI: 45.0-54.8%) patients ~~were identified as having a~~had single infection, ~~and~~ while 52 (13.1%; 95% CI: 9.8-16.4%) patients ~~were documented as~~had ~~having~~ multiple infection~~s~~. The most prevalent pathogen identified ~~were~~was *M. pneumoniae* in 95 (23.9% of the total samples) ~~cases~~patients, followed by AdV in 46 (11.6%) ~~samples~~patients, Flu A/H3N2 in 44 (11.1%) ~~samples~~patients, HRhV in 32 (8.1%) ~~samples~~patients, Flu B/Yamagata in 25 (6.3%)~~ c~~ ~~samples~~patients, and Flu A /pH1N1~~–~~ in 16 (4.0%) ~~samples~~patients. Other viruses, including PIV t~~T~~ype 1, HCoV t~~T~~ype NL63, HCoV t~~T~~ype 229E, HCoV t~~T~~ype HKU1, PIV t~~T~~ype 3, HMPV, PIV ~~T~~type 4, HCoV t~~T~~ype OC43, Flu B/Victoria, RSV t~~T~~ype B and HBoV, were detected in a ~~proportion ranging from~~ 0.3% to 2.0% of ~~infection~~ samples (Table 2). The most frequently detected pathogens in patients with multiple ~~infection~~infections were *M. pneumoniae* (84.6%, 44/52), AdV (~~-~~28.8%, 15/52), HRhV (25.0%, 13/52), and Flu A/H3N2 (17.3%, 9/52).

Clinical and epidemiologic characteristics

Pneumonia (222 cases, 55.9%) was the most common clinical diagnosis made by clinicians on admission~~ and~~, followed by bronchiolitis (68 cases, 17.1%). The most common ~~symptom~~symptoms on admission ~~was~~were cough (99.2%) and sputum production (88.4%),

followed by thoracalgia (7.1%) and pharyngalgia (6.8%). Of the 397 SARI patients, a temperature ≥ 39°C was recorded in 189 SARI patients (47.6%) on admission. A total of 382 patients (96.2%) ~~had the~~ underwent chest CT ~~performed~~, ~~in which~~of whom 258 (67.5%) were reported to have ~~the presence of~~ radiographic evidence of pneumonia;~~,~~ ~~and~~ the ~~residual~~remaining 15 patients ~~didn't accept the~~did not undergo chest CT examination. Thirty-two SARI patients had ~~exposures with~~exposure to a patient with fever and respiratory symptoms ~~patients~~ , while 30 SARI patients ~~contacted~~had contact with live poultry 2 weeks before ~~their~~ illness onset. ~~Within~~ Among the 397 patients, only 5 patients had received a pneumococcal conjugate vaccine, and 1 patient was vaccinated against ~~Flu, respectively~~influenza (Table 3). No significant differences in the proportions of clinical and epidemiologic characteristics between SARI patients with confirmed ~~pathogen~~pathogens and those without confirmed ~~pathogen~~pathogens were ~~attained~~ found, except for chest radiographic examination findings. As illustrated in ~~table~~Table 4, the differences ~~of~~in the proportions of dyspnea, radiographic diagnosis of pneumonia and the presence of at least one comorbidity among patients ~~those single-~~infected ~~patients by~~with only one of the 6 ~~kinds of~~ main pathogens, including *M. pneumoniae*, AdV, HRhV, Flu A/H3N2, Flu A /pH1N1~~–~~ and Flu B/Yamagata, were statistically significant. Notably, the proportion of patients with ~~the presence of~~ radiographic evidence of pneumonia was ~~the~~ highest in patients infected by *M. pneumonia* (74.5%), and dyspnea was the most common presentation in patients with HRhV (21.1%).

Seasonal trends

Figure 1 ~~showed~~shows ~~the~~ monthly variations in the number of SARI ~~patient~~patients infected ~~identified as having~~with *M. pneumoniae*, AdV, Flu A/H3N2, Flu A /pH1N1, HRhV, and Flu B/Yamagata ~~infection~~infections. Over the 12-month period, the temporal distribution of

pathogen-confirmed SARI patients had a bimodal shape, with the first peak in the summer and the second peak in the winter. The duration of the first positive peak was 2 months, from August to September, but the second peak ~~only~~ lasted only ~~for~~ 1 month. ~~Peaks of~~The ~~pathogenpathogens~~infection peaks seemed to be ~~more~~ attributable to the number of *M. pneumoniae* and AdV cases ~~detection. Besides~~detected. In addition, Flu A/H3N2 ~~was responsible for~~contributed to the summer peak, whereas Flu B/Yamagata and Flu A/pH1N1 ~~were~~ dominantly ~~representative of~~contributed to the winter peak. Unlike other ~~pathogen~~pathogens, HRhV ~~appeared to~~was ~~be~~ detected all year along and did not show apparent seasonality. ~~Distributions of~~ The distributions of the seasonal ~~pattern~~patterns of the positiv~~e~~ity ~~rate~~rates of the main 6 pathogens ~~were~~are shown in Figure 2. ~~The prevalence of~~ Flu A/H3N2 prevalence peaked in summer (Jun-Aug) and autumn (Sep-Nov), with positiv~~e~~ity ~~rate being~~rates of 21.1% (20/95) and 22.3% (21/94), respectively (*P*>0.05). However, Flu A/pH1N1 and Flu B/Yamagata peaked in winter (Dec-Feb), with positivity~~e~~ ~~rate being~~rates of 9.8% (13/132) and 18.9% (25/132), respectively;~~,~~ ~~and~~ the ~~difference~~differences were statistically significant (*P*<0.01). ~~It's~~It is worth noting that no SARI patients ~~linked to~~ infected by Flu B/Yamagata ~~infection~~ were detected in spring (Mar-May), summer ~~and~~or autumn. The positivity rate of *M. pneumoniae* ~~SignificantlyA~~was significantly higher ~~positive rate of *M. pneumoniae* was observed~~ in autumn (43.6%, 41/94)~~, as compared with~~ than in other ~~season~~seasons (*P*<0.01). The positivity rate (18.4%, 14/76) of HRhV was significantly higher in spring ~~had a positive rate (18.4%, 14/76) significantly higher~~ than that ~~of~~in the other ~~season~~seasons (*P*<0.01). The positivity~~e~~ rate of AdV did not demonstrate obvious seasonality throughout the year (*P*>0.05).

Age distribution

The age group distributions of the positivity~~e~~ rates of the main pathogens, ~~in SARI patients identified as~~ *M. pneumoniae*, AdV, Flu A/H3N2, Flu A/pH1N1, HRhV, and Flu B/Yamagata, ~~infection were~~are shown in Figure 3. The prevalence rates of Flu A/pH1N1 (8.0%) and AdV (20.0%) peaked in the group younger than 30 ~~year~~years old, although the difference was not significant (P>0.05). The positivity~~e~~ ~~rate~~rates of *M. pneumonia* (36.2%) and Flu B/Yamagata (6.9%) were the highest in the ~~group of~~ 40-59~~-~~-year~~-old~~-old group, without statistical significance (P>0.05). Moreover, no significant differences ~~between~~ among the different age groups ~~was~~ were observed with regard to the positiv~~e~~ity rates of Flu A/H3N2 and HRhV. Interestingly, no patients infected with Flu A/H3N2 and HRhV were detected in the 30- to 39~~-~~-year~~-~~-old group.

Treatment and prognosis

The median duration from illness onset to admission ~~for~~ in SARI patients was 3 days (IQR: 2-5.5; range: 0 to 14 days), and the median duration of hospitalization was 10 days (IQR: 8-13 days). Complications ~~were present~~occurred in 61 SARI patients, with electrolyte metabolism disorder (19 cases), respiratory failure (14 cases) and cardiac insufficiency (8 cases) being the most common ~~as compared with other~~ complications. The ~~remainder~~remaining 336 patients ~~didn't~~did not report any complications. No significant differences between SARI patients with confirmed ~~pathogen~~pathogens and those without confirmed ~~pathogen~~pathogens were observed with regard to the use of antibiotics (levofloxacin, cephalosporin, azithromycin), antivirals (oseltamivir), ~~glucocorticoid~~glucocorticoids and oxygen therapy (P>0.05). The duration of antibiotic use during hospitalization was 1-15 days (median: 9 days [IQR 5-11]) ~~for~~ in SARI patients without confirmed ~~pathogen~~pathogens and 1-20 days (median: 9 days [IQR 6-11]) ~~for~~ in those with confirmed ~~pathogen~~pathogens, ~~with~~ though the difference ~~being~~ was non~~in~~significant (P=0.68). Three SARI

patients died during hospitalization (Table 5).

Discussion

Hospital-based sentinel surveillance ~~associated with~~of SARI can ~~provide~~ be used as a ~~mechanism~~ strategy to monitor trends in this relatively severe disease and is critical for establishing a platform to understand the epidemiologic and etiologic profiles at the local level. A monitoring study ~~with regard to~~involving SARI patients in Georgia demonstrated that the proportions of patients positive for respiratory pathogens varied widely between seasons; there was~~,~~ ~~from~~ no influenza ~~positive for any~~detected ~~of influenza~~ in summer and early autumn (from July to October) ~~to~~ but a 30% ~~for~~ RSV positivity rate ~~in~~ from March ~~in~~ 2015–2017[1]. Another surveillance study ~~of~~ involving SARI patients in several countries found that the positiveity rates of influenza viruses varied widely depending on country and season, from 2.1% in Armenia in 2011–2012 to 100% in Albania in 2009–2010 [18]. A comparative study of viral ~~profile~~profiles in hospitalized pediatric SARI ~~children~~ patients in Beijing and Shanghai, China, showed different viral profile patterns ~~of viral profiles~~ in the 2 cities;~~,~~ ~~in which~~ RSV (52.9%) and HRhV/enterovirus (34.7%) were the most prevalent etiological agents of SARI in Beijing, whereas HRhV/enterovirus (33.6%) and HBoV (17.7%) were the main pathogens of SARI in Shanghai [10]. The early detection of divergent SARI pathogens through ~~the~~ sentinel surveillance ~~network~~ can measure the burden of disease on the basis of severity and better prepare a region for an emergency response. To our knowledge, this pilot study is the first study to ~~description of~~ continuously surveil~~lance~~ ~~covering~~ 19 respiratory pathogens ~~among~~ in adult SARI patients in Shanghai, ~~of~~ eastern China, ~~which has~~ providinged an ~~better~~ improved understanding of the epidemiology, etiologic spectrum and clinical profile of SARI. During 1 year of active surveillance, 397 patients who met the established case definition

of SARI were eligible for ~~enrolling~~enrollment in this study, and 63.0% of these patients~~ were~~ tested positive for at least one pathogen. Our ~~finding~~findings ~~reached consensus~~were in accordance with those reported elsewhere, which revealed etiologies ~~ranging from~~in 50% to 85% of hospitalized SARI cases [7, 19-20].

~~During the phase f~~From April 2017 to March 2018 in Jinshan district, the main etiologies of SARI varied seasonally;~~,~~ ~~and~~ *M. pneumoniae*, AdV, Flu A/H3N2, HRhV, Flu B/Yamagata, ~~together with~~and Flu A-/pH1N1 were the predominant pathogens depending on the month. Other viruses, such as PIV t~~T~~ype 1, HCoV t~~T~~ype NL63, HCoV t~~T~~ype 229E, HCoV t~~T~~ype HKU1, PIV t~~T~~ype 3, HMPV, PIV t~~T~~ype 4, HCoV t~~T~~ype OC43, Flu B/Victoria, RSV t~~T~~ype B and HBoV, were also present, although the numbers of patients infected~~ of~~ with these ~~infrequent~~ viruses ~~was~~ were relatively small. Since our ~~sentinel~~ surveillance system aimed~~s~~ to detect SARI~~ a~~ ~~t~~in adult ~~SARI~~ patients, most of the enrolled patients were ~~the elderly~~elderly individuals aged ~~between~~ 60-79 years ~~old~~ (52.1%) ~~and those aged~~ 80 years and above (22.2%). Our study demonstrates that individuals in the over ~~the age of~~ 60 age group are the most vulnerable ~~group for suffering from~~ to SARI in Jinshan, a subtropical region. In the present study, at least one chronic medical condition ~~occurred~~ was present in 70% of SARI patients. Our study population ~~presented~~ had a high prevalence of comorbidities compared with that in ~~the~~ a study in Hubei ~~province~~Province, China [12]~~,~~. ~~and~~ This may be partially explained by the ~~inconsistence of socio-economic~~inconsistency ~~of~~in socioeconomic development between the 2 regions. Hypertension and cardiovascular disease ~~was~~were observed in 38.3% and 7.6% of our population, respectively. ~~And patients~~ Patients with confirmed ~~pathogen~~pathogens had a higher prevalence of cardiovascular disease than those without confirmed ~~pathogen~~pathogens. One study suggested that diagnosed cardiovascular disease

was ~~commonly~~ related to a fatal ~~endpoints~~ outcome ~~among~~ in influenza~~-~~-positive SARI ~~cases~~ patients [21]. Our study revealed that the proportions of patients ~~vaccinating~~ who received influenza ~~vaccine~~vaccines and pneumococcal conjugate ~~vaccine~~vaccines ~~was~~ were quite low, so ~~the~~ respiratory disease vaccination programs ~~with the target of~~ targeting individuals with cardiovascular~~-~~-related ~~disease~~diseases should be recommended. In this study, most patients presented with cough, sputum production and fever. These clinical features bear some resemblance to ~~the report~~those reported in a previous study [1]. It should be ~~alerted~~noted that empirical ~~use~~ administration of antibiotics ~~use~~ during hospitalization occurred in 99% of patients in the present study due to the unavailability of ~~rapid~~rapid~~ly~~ pathogen identification ~~determining etiological diagnoses~~tests. The current study found that pneumonia was the main reason for hospital admission ~~among~~ of SARI patients ~~with SARI~~ (55.9%)~~ and~~, followed by bronchiolitis (17.1%) in Jinshan, a region in eastern China. A similar study in northern China showed that pneumonia (88.95%) and bronchiolitis (6.37%) were also ~~were~~ the top 2 admission ~~diagnosis~~diagnoses ~~of~~ among SARI patients [22]. HRhV has emerged as an independent causative agent ~~in~~ of lower respiratory tract infection~~s~~. ~~So far~~To date, the majority of investigations ~~about~~on HRhV-associated lower respiratory tract infection~~s~~ in adults ~~focus~~have focused on ~~the~~ immunocompromised ~~cases~~ patients [23-25] or those with hospital-acquired pneumonia [26-27]. We compared the ~~single-infected~~ patients with single infection ~~groups~~ in terms of signs and symptoms ~~in this study~~, and the results showed that ~~the~~ dyspnea was the most frequent symptom (21.1%) ~~for~~ in community-acquired SARI patients infected by HRhV, which was ~~comparatively~~ consistent ~~to~~with the results of a similar multicenter study (30%) in China [28]. *M. pneumoniae* is an important cause of community-acquired pneumonia. Depending on the setting, 10%-40% of

community-acquired pneumonia patients are caused by haveare infected with *M. pneumoniae* [20]. Our study also showed that patients infected by *M. pneumoniae* presented had the highest rate of radiographic evidence of pneumonia (74.5%) compared with those single- infected by other single pathogenpathogens, which demonstratinged that community-acquired pneumonia was is a heterogeneous disease. It was worth noting, of OfAmong the 382 SARI patients thatwho had a underwent chest CT performed, that thethere was a significant difference ofin the proportion of patients who accepted aing chest radiographic examination between SARI patients with confirmed pathogenpathogens and those without confirmed pathogenpathogens was statistically significant. However, thea significant difference ofin the proportion of patients presenting a radiographic diagnosis of pneumonia between SARI patients with confirmed pathogenpathogens and those without confirmed pathogen werepathogens was not observed, which suggestinged that the etiologies and disease courses of community-acquired pneumonia were highly variable.

M. pneumoniae (23.9%) was the most common pathogen in the present study. The positive detection rate of *M. pneumoniae* echoed was similar to thethe published data rate (19.7%) in northnorthern China [20]. A prospective study conducted in Hong Kong among including adults hospitalized with pneumonia in from 2004 to 2005 found that *M. pneumoniae* was detected in 78/1,193 patients (6.5%) [29]. *M. pneumoniae* occurs endemically worldwide in many different geographic climatesregions.- *M. pneumoniae* was mostly detected in autumn (43.6%) and spring (27.6%) in our study, but *M. pneumoniae* in Istanbul was more commonly identified in summer (44.9%) and winter (22.4 %4%) [30]. As the second most common pathogen in this study, the positivitye rate of AdV did not significantly differ along with season changeseasonal changesseasonally;, and this trend in seasonality was consistent with previousthe previously

reported AdV seasonality ~~of~~ data from~~the AdV detecting~~detection ~~rate in~~ China [10]. In contrast with the seasonality of viral SARI observed in Georgia in 2015-2017 and in northern China in 2014-2016, where a~~the~~ distinct winter-only influenza peak ~~of influenza circulation~~ was observed [31,32], we found that influenza peaked ~~both~~ in both the winter and in summer. Overall, influenza virus was common in this study, with Flu A/H3N2 dominating in summer~~,~~ and Flu B/Yamagata and Flu A/pH1N1 dominating in winter. According to ~~O~~our findings, ~~found that~~ the positivit~~y~~e rate of Flu B/Yamagata (18.9%) ~~were~~was nearly twice that of Flu A/pH1N1 (9.8%) in winter;~~,~~ ~~which~~this result was different from ~~one~~ that of a study in the USA in which estimated excess hospitalization rates associated with ~~Flu~~ influenza B were lower than ~~for~~those associated with Flu A/H3N2 [33]. In this study, we also noted that no ~~statistical~~significant differences ~~were~~~~was~~were found in the positiv~~e~~ity rates ~~of pathogens identified as~~ *M. pneumoniae*, AdV, Flu A/H3N2, Flu A /pH1N1, HRhV, and Flu ~~B /Yamagata among~~B/Yamagata among the different age ~~group~~groups. This ~~phenomenon~~ result was basically the same as that in ~~the~~a previous study in China [10]~~,~~ and may be attributable to susceptibility to these common viruses in different age ~~group~~groups of adults. As reported elsewhere [34], ~~co-infections~~coinfections were ~~found~~ relatively common in the present study. A total of 13.1% of SARI patients were reported to have more than one pathogen infection; this~~, and the~~ percentage was consistent with ~~the finding of~~that in a previous study (11.7%) [19].

Limitations

Our study was subject to several limitations. First, as a pilot ~~project~~study, this study was conducted at only 1 hospital.~~,~~ ~~although~~Even though this hospital is the ~~biggest~~largest hospital in the district, ~~so~~ the ~~finding~~findings may have relatively limited generalizability.~~ Actually,~~ ~~t~~The

prevalence of each pathogen may vary ~~at~~in regions ~~having~~with different ~~climatic~~climates, demographic patterns and accessibility to healthcare. Second, the result was based on SARI surveillance ~~of~~ over a 12-month period, and the burden ~~derived~~ due to~~from~~ SARI may not reflect the actual situation over several years. Third, the case report form in this study ~~is~~ was ~~the~~ a standard structured~~al~~ questionnaire, and ~~justonly~~ the results were collected ~~the result~~to determine whether ~~has the presence of~~~~there~~the patient had received ~~was~~ a radiographic diagnosis of pneumonia. It was impossible to pinpoint the type of pneumonia, such as ~~the~~ lobar pneumonia ~~and~~ or atypical pneumonia. The pathogens ~~tested~~ detected in this pilot~~ing~~ study ~~only~~ covered only common respiratory viruses and *M. pneumoniae*~~,~~ and did not include related respiratory ~~bacterium~~bacterial pathogens, such as *P~~p~~neumococcus* and *Bordetella pertussis*, owing to limited financial support, so ~~the~~ SARI patients without confirmed ~~pathogen~~pathogens may ~~indicate~~ have been positive for other ~~non-tested~~nontested bacterial ~~etiology~~~~etiologies~~pathogens. Indeed, the inclusion of ~~bacterium~~bacterial surveillance is under consideration ~~to integrate~~for integration into our program.

Conclusions

In conclusion, the current study ~~is~~ was the first ~~study which monitors~~ to monitor hospitalized adult SARI patients for most respiratory viruses and *M. pneumoniae* in Shanghai~~,~~ and confirmed~~s~~ that multiple respiratory pathogens may circulate among the SARI population and vary with ~~the~~ climatic and demographic characteristics. ~~The~~This finding highlights the importance of sustained~~ing~~ sentinel surveillance of SARIs ~~patients~~ at the local and national levels, which can ~~contribute to~~guide accurate~~ly~~ ~~evaluate~~evaluations of~~ng~~ the prevalence of etiological agents of~~y~~ ~~associated to~~~~with~~ SARI and the burden of disease~~,~~ and, most ~~more~~ importantly, ~~to shape~~shaping

public polici~~y~~es on SARI prevention and responses to SARI activity.

Supporting information

S1 File. Minimal data set.

S2 File. Sequences of primers targeting Flu A/B used in real-time RT-PCR.

Acknowledgment~~Acknowledgements~~

We would like to express our gratitude to the physicians and nurses from Jinshan ~~district central hospital~~District Central Hospital for their significant contribution to the data collection and specimen collection.

Funding

This work was supported by the Research Project of Shanghai Municipal Health Commission (201940428) and the Infectious Disease and Epidemiology Project of the 6th Jinshan District Medical Key Specialty Construction (JSZK2019B05). The funder had no role in the study design, data collection and analysis, decision to publish or preparation of the manuscript.

Availability of data and materials

All relevant data are contained within the manuscript and its Supporting Information files.

Author Contributions

Conceptualization: Jian Li, Jian-Min Zhu

Data curation: Can-Lei Song, Tang Wang, Shu-Hua Li

Formal analysis: Jian Li, Can-Lei Song, Tang Wang

Funding acquisition: Can-Lei Song, Shu-Hua Li

Investigation: Can-Lei Song, Tang Wang, Shu-Hua Li–

Methodology: Jian Li, Can-Lei Song, Yu-Long Ye , Jian-Ru Du

Project administration: Jian-Min Zhu

Supervision: Can-Lei Song, Tang Wang, Jian-Min Zhu

Validation: Jian Li, Can-Lei Song, Tang Wang, Yu-Long Ye, Jian-Ru Du, Shu-Hua Li, Jian-Min Zhu

Writing-original draft: Jian Li, Can-Lei Song, Tang Wang

Writing-review & editing: Jian Li, Can-Lei Song, Tang Wang, Yu-Long Ye, Jian-Ru Du, Shu-Hua Li, Jian-Min Zhu

Competing interests

The authors declare that they have no competing interests.

References

1. Chakhunashvili G, Wagner AL, Power LE, Janusz CB, Machablishvili A, Karseladze I, et al. Severe Acute Respiratory Infection (SARI) sentinel surveillance in the country of Georgia, 2015-2017. PLos One.2018;13(7):e0201497. https://doi.org/10.1371/journal.pone.0201497 PMID:30059540. PubMed Central PMCID: PMC6066249.
2. Elhakim MM, Kandil SK, Abd Elaziz KM, Anwar WA. Epidemiology of Severe Acute Respiratory Infection (SARI) cases at a sentinel site in Egypt, 2013-15. J Public Health (Oxf). 2020;42(3):525-33.https:// doi.org/ 10.1093/pubmed/fdz053 PMID: 31090911. PubMed Central PMCID: PMC7107553.
3. Williams B, Gouws E, Boschi-Pinto C, Bryce J, Dye C. Estimates of world-wide distribution of child deaths from acute respiratory infections. Lancet Infect Dis. 2002;2(1):25–32. https:// doi.org/10.1016/s1473-3099(01)00170-0 PMID: 11892493.
4. WHO. The global burden of disease: 2004 update. May 21th, 2020. Available from:

http://www.who.int/healthinfo/global_burden_disease/GBD_report_2004update_full.pdf.

5. Watt JP, Wolfson LJ, O’Brien KL, Henkle E, Deloria-Knoll M, McCall N,et al. Burden of disease caused by Haemophilus influenzae type b in children younger than 5 years: global estimates. Lancet. 2009;374(9693): 903–11. https:// doi.org/10.1016/S0140-6736(09)61203-4 PMID: 19748399.

6. Subramoney K, Hellferscee O, Pretorius M, Tempia S, McMorrow M, von Gottberg A, et al. Human bocavirus, coronavirus,and polyomavirus detected among patients hospitalised with sev ere acute respiratory illness in South Africa, 2012 to 2013. Health Sci Rep. 2018;1(8):e59. https:// doi.org/10.1002/hsr2.59 PMID: 30623094. PubMed Central PMCID: PMC6266378.

7. Razanajatovo NH, Guillebaud J, Harimanana A, Rajatonirina S, Ratsima EH, Andrianirina ZZ, et al. Epidemiology of severe acute respiratory infections from hospital-based surveillance in Madagascar, November 2010 to July 2013. PLos One.2018;13(11):e0205124. https:// doi.org/10.1371/journal.pone.0205124 PMID: 30462659. PubMed Central PMCID: PMC6248916.

8. WHO. WHO global technical consultation: global standards and tools for influenza surveillance. May 21th, 2020. Available from: https://extranet.who.int/iris/restricted/bitstream/handle/10665/70724/WHO_HSE_GIP_2011.1_eng.pdf;jsessionid=53A32B0F697E2FEDAA77BF5F971ED037?sequence=1.

9. Byington CL, Enriquez FR, Hoff C, Tuohy R, Taggart EW, Hillyard DR, et al. Serious bacterial infections in febrile infants 1 to 90 days old with and without viral infections. Pediatrics. 2004;113(6):1662–6. https:// doi.org/10.1542/peds.113.6.1662 PMID: 15173488.

10. Zhao Y, Lu R, Shen J, Xie Z, Liu G, Tan W. Comparison of viral and epidemiological profiles

of hospitalized children with severe acute respiratory infection in Beijing and Shanghai, China. BMC Infect Dis. 2019;19(1):729. https:// doi.org/10.1186/s12879-019-4385-5 PMID: 31429710. PubMed Central PMCID: PMC6701130.

11. Nguenha N, Tivane A, Pale M, Machalele L, Nacoto A, pPires G, et al. Clinical and epidemiological characterization of influenza virus infections in children with severe acute respiratory infection in Maputo, Mozambique: Results from the implementation of sentinel surveillance,2014-2016. PLoS One.2018;13(3):e0194138. https:// doi.org/10.1371/journal.pone.0194138 PMID: 29590162. PubMed Central PMCID: PMC5874022.

12. Yu H, Huang J, Huai Y, Guan X, Klena J, Liu S, et al. The substantial hospitalization burden of influenza in central China: surveillance for severe, acute respiratory infection, and influenza viruses, 2010-2012.Influenza Other Respir Viruses. 2014; 8(1):53-65. https:// doi.org/10.1111/irv.12205 PMID: 24209711. PubMed Central PMCID: PMC4177798.

13. Fica A, Sotomayor V, Fasce R, Dabanch J, Soto A, Charpentier P, et al. Severe acute respiratory infections (SARI) from influenza in adult patients in Chile: the experience of a sentinel hospital. Rev Panam Salud Publica. 2019;43:e1. https://doi.org/10.26633/RPSP.2019.1 PMID:31093225. PubMed Central PMCID: PMC6393720.

14. Foy HM, Cooney MK, McMahan R, et al. Viral and mycoplasmal pneumonia in a prepaid medical care group during an eight-year period. Am J Epidemiol. 1973; 97: 93-102. https://doi.org/10.1093/oxfordjournals.aje.a121493 PMID: 4347511.

15. Waites KB, Xiao L, Liu Y, Balish MF, Atkinson TP. Mycoplasma pneumoniae from the respiratory tract and beyond. Clin Microbiol Rev.2017; 30: 747-809.

https://doi.org/10.1128/CMR.00114-16 PMID: 28539503. PubMed Central PMCID: PMC5475226.

16. Bureau of statistics of Jinshan district. Jinshan statistical yearbook 2018. May 20th, 2020. Available from: http://www.jinshan.gov.cn/Attach/Attaches/201911/201911140517237545.pdf.

17. WHO. Manual for the laboratory diagnosis and virological surveillance of influenza. May 21th, 2020. Available from:http://whqlibdoc.who.int/publications/2011/9789241548090_eng.pdf.

18. Meerhoff TJ, Simaku A, Ulqinaku D, Torosyan L, Gribkova N, Shimanovich V, et al. Surveillance for severe acute respiratory infections (SARI) in hospitals in the WHO European region—an exploratory analysis of risk factors for a severe outcome in influenza-positive SARI cases. BMC Infect Dis. 2015; 15: 1.https://doi.org/10.1186/s12879-014-0722-x PMID: 25567701. PubMed Central PMCID: PMC4314771.

19. Huo X, Qin Y, Qi X, Zu R, Tang F, Li L, et al. Surveillance of 16 respiratory viruses in patients with influenza-like illness in Nanjing, China. J Med Virol. 2012; 84(12):1980-4. https://doi.org/ 10.1002/jmv.23401 PMID: 23080506. PubMed Central PMCID: PMC7166984.

20. Breiman RF, Cosmas L, Njenga M, Williamson J, Mott JA, Katz MA, et al. Severe acute respiratory infection in children in a densely populated urban slum in Kenya, 2007–2011. BMC Infect Dis. 2015; 15: 95.https://doi.org/10.1186/s12879-015-0827-x PMID: 25879805. PubMed Central PMCID: PMC4351931.

21.Butsashvili M, Kandelaki G, Zakhashvili K, Machablishvili A, Avaliani N. Influenza-associated mortality in georgia (2009-2011). Influenza Res Treat. 2012;2012:480763. https://doi.org/10.1155/2012/480763 PMID: 23074667.PubMed Central

PMCID: PMC3447290.

22. Xu W, Guo L, Dong X, Li X, Zhou P, Ni Q, et al. Detection of viruses and mycoplasma pneumonia in hospitalized patients with severe acute respiratory infection in northern China, 2015-2016. Jpn J Infec Dis. 2018; 71(2):134-139. https://doi.org/10.7883/yoken.JJID.2017.412 PMID: 29491245.

23. Costa C, Bergallo M, Astegiano S,Sidoti F, Terlizzi ME, Gambarino S, et al. Detection of human rhinoviruses in the lower respiratory tract of lung transplant recipients. Arch Virol 2011;156(8):1439-43. https://doi.org/10.1007/s00705-011-0986-z PMID: 21461933. PubMed Central PMCID: PMC7087099.

24. Hicks LA, Shepard CW, Britz PH, Erdman DD, Fischer M, Flannery BL, et al. Two outbreaks of severe respiratory disease in nursing homes associated with rhinovirus. J Am Geriatr Soc 2006; 54(2):284-9. https://doi.org/10.1111/j.1532-5415.2005.00529.x PMID: 16460380.

25. Mikulska M, Del Bono V, Gandolfo N, Dini S, Dominietto A, Di Grazia C, et al. Epidemiology of viral respiratory tract infections in an outpatient haematology facility. Ann Hematol 2014;93(4):669-76. https://doi.org/10.1007/s00277-013-1912-0 PMID: 24097084. PubMed Central PMCID: PMC7079995.

26. Louie JK, Yagi S, Nelson FA, Kiang D, Glaser CA, Rosenberg J, et al. Rhinovirus outbreak in a long term care facility for elderly persons associated with unusually high mortality. Clin Infect Dis 2005;41(2):262-5. https://doi.org/10.1086/430915 PMID: 15983926. PubMed Central PMCID: PMC7107978.

27. Wald TG, Shult P, Krause P, Miller BA, Drinka P, Gravenstein S. A rhinovirus outbreak among residents of a long-term care facility. Ann Intern Med 1995;123(8):588-93.

https://doi.org/10.7326/0003-4819-123-8-199510150-00004 PMID: 7677299.

28. Wang KQ, Xi W, Yang DH, Zheng YL, Zhang YQ, Chen YS, et al. Rhinovirus is associated with severe adult community-acquired pneumonia in China. J Thorac Dis; 2017;9(11):4502-4511. https://doi.org/10.21037/jtd.2017.10.107 PMID: 29268520. PubMed Central PMCID: PMC5721016.

29. Lui G, Ip M, Lee N, Rainer TH, Man SY, Cockram CS, et al. Role of 'atypical pathogens' among adult hospitalized patients with community-acquired pneumonia. Respirology.2009; 14(8):1098–1105. https://doi.org/10.1111/j.1440-1843.2009.01637.x PMID: 19818051.

30. Goktas S, Sirin MC. Prevalence and seasonal distribution of respiratory viruses during the 2014 – 2015 season in Istanbul. Jundishapur J Microbiol. 2016; 9(9): e39132. https://doi.org/10.5812/jjm.39132 PMID: 27800148. PubMed Central PMCID: PMC5086027.

31. Azziz Baumgartner E, Dao CN, Nasreen S, Bhuiyan MU, Mah-E-Muneer S, Ai Mamun A. et al. Seasonality, timing, and climate drivers of influenza activity worldwide. J Infect Dis. 2012; 206(6):838-46. https://doi.org/10.1093/infdis/jis467 PMID: 22829641.

32. Zhang Y, Muscatello DJ, Wang Q, Yang P, Pan Y, Huo D, et al. Hospitalizations for influenza-associated severe acute respiratory infection, Beijing, China, 2014-2016. Emerg Infect Dis. 2018;24(11):2098-2102.https://doi.org/10.3201/eid2411.171410 PMID: 30334719. PubMed Central PMCID: PMC6199980.

33. Thompson WW, Shay DK, Weintraub E, Brammer L, Bridges CB, Cox NJ, et al. Influenza-associated hospitalizations in the United States. JAMA. 2004; 292(11):1333–1340. https://doi.org/10.1001/jama.292.11.1333 PMID: 15367555.

34. Hasman H, Pachucki CT, Unal A, Nguyen D, Devlin T, Peeples ME, et al. Aetiology of influenza-like illness in adults includes parainfluenzavirus type 4. J Med Microbiol. 2009;58 (Pt4):408–413. https://doi.org/10.1099/jmm.0.006098-0 PMID: 19273634. PubMed Central PMCID: PMC2778239.

Table 1. Demographic characteristics of adult SARI patients in a surveillance hospital in Jinshan, Shanghai, April 2017 to March 2018

Characteristics	SARI patients			*P* value*
	All (%) [n=397]	With confirmed pathogens (%) [n=250]	Without confirmed pathogens (%) [n=147]	
~~Gender~~Sex				0.315
Male	194(48.9)	127(50.8)	67(45.6)	
Female	203(51.1)	123(49.2)	80(54.4)	
Age group (median, years)	68.0	67.0	69.0	0.357
<30	25(6.3)	17(6.8)	8(5.4)	0.786
30-39	19(4.8)	10(4.0)	9(6.1)	
40-59	58(14.6)	39(15.6)	19(12.9)	
60-79	207(52.1)	128(51.2)	79(53.7)	
≥80	88(22.2)	56(22.4)	32(21.9)	
BMI				0.657
<20	118(29.7)	73(29.2)	45(30.6)	
20-25	208(52.4)	135(54.0)	73(49/7)	
>25	71(17.9)	42(16.8)	29(19.7)	
Chronic medical conditions				
At least one	278(70.0)	178(71.2)	100(68.0)	0.505

Asthma	12(3.0)	6(2.4)	6(4.1)	0.345
Chronic bronchitis	49(12.3)	30(12.0)	19(12.9)	0.787
COPD	28(7.1)	13(5.2)	15(10.2)	0.060
Hypertension	152(38.3)	95(38.0)	57(38.8)	0.878
Cardiovascular disease	30(7.6)	22(8.8)	8(5.4)	0.222
Diabetes	61(15.4)	38(15.2)	23(15.6)	0.905
Cerebrovascular disorder	20(5.0)	14(5.6)	6(4.1)	0.504
Tumor	19(4.8)	14(5.6)	5(3.4)	0.322

*~~The~~ *P* values ~~denoted~~denote comparisons between SARI patients with confirmed ~~pathogen~~pathogens and SARI patients without confirmed ~~pathogen~~pathogens.

Table 2. Etiological agent~~y~~ distributions ~~of~~ among adult SARI patients in a surveillance hospital in Jinshan, Shanghai, April 2017 to March 2018

Etiological agent~~y~~	Frequency# (n)	Percent of samples* (%)
Influenza virus A		
pH1N1	16	4.0
H3N2	44	11.1
Influenza virus B		
Yamagata	25	6.3
Victoria	2	0.5
Parainfluenza virus		
Type 1	8	2.0
Type 2	0	0
Type 3	6	1.5
Type 4	5	1.3
Human coronavirus		
Type 229E	6	1.5
Type OC43	4	1.0

Type HKU1	6	1.5
Type NL63	8	2.0
Respiratory syncytial virus		
Type A	0	0
Type B	2	0.5
Human rhinovirus	32	8.1
Adenovirus	46	11.6
Human metapneumovirus	6	1.5
Human bocavirus	1	0.3
Mycoplasma pneumoniae	95	23.9
Single infection	198	49.9
Multiple infection		
2 pathogens	43	10.8
3 pathogens	8	2.0
4 pathogens	1	0.3

[#]The frequency of each pathogen may include both the samples ~~of~~ with single infection and those with multiple infection, and their total number is larger than the sum of samples with single infection and multiple infection. *Percent of samples ~~referred to~~is the frequency of samples with a positive etiology divided by the total enrolled samples (397 cases).

Table 3. Clinical and epidemiologic characteristics of adult SARI patients in a surveillance hospital in Jinshan, Shanghai, April 2017 to March 2018

Characteristics	SARI patients			*P* value*
	All (%) [n=397]	With confirmed pathogens (%) [n=250]	Without confirmed pathogens (%) [n=147]	
Temperature ≥39°C	189(47.6)	126(50.4)	63(42.9)	0.176
Cough	394(99.2)	249(99.6)	145(98.6)	0.558
Sputum production	351(88.4)	219(87.6)	132(89.8)	0.509
Pharyngalgia	27(6.8)	18(7.2)	9(6.1)	0.680
Thoracalgia	28(7.1)	19(7.6)	9(6.1)	0.687
Dyspnea	19(4.8)	11(4.4)	8(5.4)	0.808
Runny nose	11(2.8)	7(2.8)	4(2.7)	1.000
Vomiting	15(3.8)	10(4.0)	5(3.4)	0.795
Acceptance of chest radiographic exam	382(96.2)	236(94.4)	146(99.3)	**0.013**
Presence of radiographic diagnosis of pneumonia	258/382(67.5)	153/236(64.8)	105/146(71.9)	0.349
Visited~~ing~~ a live poultry market	3(0.8)	3(1.2)	0(0)	0.299
Contact with live poultry	30(7.6)	19(7.6)	11(7.5)	1.000
Contact with patient with fever	32(8.1)	24(9.6)	8(5.4)	0.182
Smoking				0.860
Current~~s~~	43(10.8)	28(11.2)	15(10.2)	
Former	66(16.6)	43(17.2)	23(15.6)	
Never	288(72.6)	179(71.6)	109(74.2)	
Vaccinated with~~ing~~ pneumococcal conjugate vaccine	5(1.3)	3(1.2)	2(1.4)	1.000

Vaccinat~~ing~~ed with influenza vaccine	1(0.3)	1(0.4)	0(0)	1.000

*~~The~~ *P* values ~~denoted~~denote comparisons between SARI patients with confirmed ~~pathogen~~pathogens and SARI patients without confirmed ~~pathogen~~pathogens.

Table 4. Comparison of characteristics of ~~single-infected~~ SARI patients infected with only one of the~~by~~ 6 main pathogens in a surveillance hospital in Jinshan, Shanghai, April 2017 to March 2018

Characteristics	*M. pneumoniae* (%) [n=51]	AdV (%) [n=31]	HRhV (%) [n=19]	Flu A/H3N2 (%) [n=35]	Flu B/Yamagata (%) [n=21]	Flu A /pH1N1 (%) [n=16]	*P* value*
~~Gender~~Sex							0.750
Male	28(54.9)	19(61.3)	8(42.1)	18(51.4)	9(42.9)	8(50.0)	
Female	23(45.1)	12(38.7)	11(57.9)	17(48.6)	12(57.1)	8(50.0)	
Age group(years)							0.247
<30	5(9.8)	3(9.7)	1(5.3)	1(2.9)	0(0)	2(12.5)	
30-39	3(5.9)	3(9.7)	0(0)	0(0)	1(4.8)	1(6.3)	
40-59	12(23.5)	1(3.2)	2(10.5)	5(14.3)	3(14.3)	2(12.5)	
60-79	20(39.2)	15(48.4)	8(42.1)	24(68.6)	12(57.1)	9(56.3)	
≥80	11(21.6)	9(29.0)	8(42.1)	5(14.3)	5(23.8)	2(12.5)	
A~~a~~t least one comorbidity	25(49.0)	23(74.2)	13(68.4)	26(74.3)	18(85.7)	11(68.8)	**0.034**
Temperature ≥39°C	30(58.8)	16(51.6)	6(31.6)	16(45.7)	9(42.9)	8(50.0)	0.444
Cough	51(100)	31(100)	19(100)	34(97.1)	21(100)	16(100)	0.705
Sputum production	39(76.5)	29(93.5)	15(78.9)	30(85.7)	19(90.5)	16(100)	0.120
Pharyngalgia	3(5.9)	3(9.7)	2(10.5)	2(5.7)	2(9.5)	2(12.5)	0.876
Thoracalgia	4(7.8)	2(6.5)	1(5.3)	0(0)	2(9.5)	1(6.3)	0.523
Dyspnea	0(0) †	1(3.2)	4(21.1) †	1(2.9)	1(4.8)	0(0)	**0.007**
Runny nose	1(2.0)	1(3.2)	1(5.3)	1(2.9)	0(0)	2(12.5)	0.360

Vomiting	0(0)	3(9.7)	0(0)	3(8.6)	1(4.8)	1(6.3)	0.123
Presence of radiographic diagnosis of pneumonia	38(74.5)#	17(54.8)	13(68.4)	15(42.9)#	13(61.9)	7(43.8)	**0.042**
Visited~~ing~~ a live poultry market	1(2.0)	1(3.2)	0(0)	0(0)	0(0)	0(0)	0.880
Contact with live poultry	6(11.8)	3(9.7)	2(10.5)	1(2.9)	1(4.8)	1(6.3)	0.753
Contact with a patient with fever	3(5.9)	4(12.9)	2(10.5)	1(2.9)	3(14.3)	2(12.5)	0.442
Current Smok~~ing~~er	2(3.9)	4(12.9)	2(10.5)	6(17.1)	3(14.3)	3(18.8)	0.333
Former Smok~~ing~~er	10(19.6)	7(22.6)	2(10.5)	7(20.0)	3(14.3)	0(0)	
Never Smok~~ing~~ed	39(76.5)	20(64.5)	15(78.9)	22(62.9)	15(71.4)	13(81.3)	

*~~The~~ *P* values ~~denoted~~denote comparisons among the six main pathogens. † and # ~~—~~ signify *P*<0.05 for pairwise comparisons. † refers~~red~~ to comparisons between the single-infected SARI patients ~~by~~ with *M. pneumoniae* and those ~~by~~ with HRhV. # ~~referred~~refers to ~~the~~ comparisons between ~~the single-infected~~ SARI patients ~~by~~ infected with *M. pneumoniae* and those ~~by~~ infected with Flu A/H3N2.

Table 5. ~~—~~ Treatments and prognos~~i~~es ~~of~~ in adult SARI patients in a surveillance hospital in Jinshan, Shanghai, April 2017 to March 2018

Characteristics	SARI patients			*P* value*
	All (%) [n=397]	With confirmed pathogens (%) [n=250]	Without confirmed pathogens (%) [n=147]	
Clinical course (median, days)				
From illness onset to admission	3	3	3	0.567
Length of hospitalization	10	10	10	0.545
Antibiotics prior to hospitalization	241（61.0）	151（60.9）	90（61.2）	0.723
Antibiotics during hospitalization	393(99.0)	246(98.4)	147(100)	0.301
Antivirals	11(2.8)	7(2.8)	4(2.7)	1.000
Glucocorticoids	112(28.2)	72(27.2)	40(28.8)	0.734
Oxygen therapy	196(49.4)	124(49.6)	72(49.0)	0.918

Complications	61(15.4)	37(14.8)	24(16.3)	0.684
Death	3(0.8)	2(0.8)	1(0.7)	1.000

*~~The~~ *P* values ~~denoted~~denote comparisons between SARI patients with confirmed ~~pathogen~~pathogens and SARI patients without confirmed ~~pathogen~~pathogens.

January 5, 2020

Dear editor,

On behalf of my co-authors, we are very appreciated to know that our manuscript (PONE-D-20-19561) is potentially acceptable for publication in PLOS ONE. We thank editors and reviewers greatly for their positive comments on our manuscript. These comments are greatly helpful in improving our manuscript and are addressed carefully. We made corresponding revisions to the manuscript according to comments. The revised manuscript highlights changes made to the original version with red color. Also, we provide a point-by-point response to each comment. The revised manuscript has been polished by a professional, native English speaker from Springer Nature for language usage, spelling, and grammar. In addition, we agree to provide the minimal data set underlying the findings as Supporting Information files for data-sharing.

We believe that the revised version of manuscript is improved highly and attached please find the revised manuscript. We ensure that our manuscript has conformed to the journal style, and we confirm that all author details on the revised version are correct, that all authors have agreed to authorship and order of authorship for this manuscript. We hope the manuscript will receive your kind consideration and be published in your valuable journal.

We would like to express our great appreciation to you and reviewers for comments on our manuscript. Looking forward to hearing from you.

Best regards.

Yours sincerely,

Jian Li

Response to Specific Comments:

1. Specimen collection and laboratory testing: This section need further clarification. Please specify the multiplex PCR used. Did the authors used method described in previous literatures or commercial kit?

   Answer: We thank for these suggestions and have made further clarification. The multiplex PCR used is the commercial kit. We added the data about multiplex PCR and made further clarification (see Page 7, line 154 to Page 8, line 156 in **Revised Manuscript with Track Changes, the same below**).

   Line 219 – 223: Not sure what the authors wish to convey, please rephrase for clarification.

   Answer: We are sorry and have rephrased these sentences (see Page 11, line232-236).

2. Line 260 – 265: Not clear on what the authors' intention on these statement, please clarify.

   Answer: These sentences in line 260-265 mean to show that there were no significant differences of therapy between SARI patients with confirmed pathogen and those without confirmed pathogen. We have modified our text as advised (see Page 13, line 280-286).

3. Line 295 – 299: This argument does not hold. These are not fair comparison since this study excluded children.

   Answer: This comment is appreciated highly. We deleted these sentences in line 295-298 following this comment, and revised the next sentence in line 298-299(see Page 15, line321-323).

4. Ethical statement: This needs to be included in the Materials and Methods section and needs to include approval number.

   Answer: The ethical statement has been moved to the Materials and Methods section, and the approval number has been added (see Page 9, line 181-186).

Response to Journal Requirements:

1. Please ensure that your manuscript meets PLOS ONE's style requirements, including those for file naming. The PLOS ONE style templates can be found at

https://journals.plos.org/plosone/s/file?id=wjVg/PLOSOne_formatting_sample_main_body.pdf and

https://journals.plos.org/plosone/s/file?id=ba62/PLOSOne_formatting_sample_title_authors_affiliations.pdf

Answer: We ensure that our manuscript meets the journal's style.

2. In your methods and ethics statement, please state whether you obtained consent from parents or guardians of minors under 18 years old.

Answer: We have stated that the consent from parents or guardians of those under 18 years old have been obtained in the section of "ethics statement" (see Page 9, line 184-186).

3. PLOS ONE requires experimental methods to be described in enough detail to allow suitably skilled investigators to fully replicate and evaluate your study. See https://journals.plos.org/plosone/s/submission-guidelines#loc-materials-and-methods for more information.
To comply with PLOS ONE submission guidelines, in your Methods section, please provide a more detailed description of your methodology, specifically about your

respiratory pathogens 15 multiplex real-time RT-PCR, Flu A/B RT-PCR, and flu typing methods.

Answer: We have provided a more detailed description of methodology in the section of specimen collection and laboratory testing as advised (see Page 7, line 142 to Page 8, line 166).

4. We note that you have indicated that data from this study are available upon request. PLOS only allows data to be available upon request if there are legal or ethical restrictions on sharing data publicly. For information on unacceptable data access restrictions, please see http://journals.plos.org/plosone/s/data-availability#loc-unacceptable-data-access-restrictions.

In your revised cover letter, please address the following prompts:

a) If there are ethical or legal restrictions on sharing a de-identified data set, please explain them in detail (e.g., data contain potentially identifying or sensitive patient information) and who has imposed them (e.g., an ethics committee). Please also provide contact information for a data access committee, ethics committee, or other institutional body to which data requests may be sent.

b) If there are no restrictions, please upload the minimal anonymized data set necessary to replicate your study findings as either Supporting Information files or to a stable, public repository and provide us with the relevant URLs, DOIs, or accession numbers. Please see http://www.bmj.com/content/340/bmj.c181.long for guidelines on how to de-identify and prepare clinical data for publication. For a list of acceptable repositories, please see http://journals.plos.org/plosone/s/data-availability#loc-recommended-repositories.

We will update your Data Availability statement on your behalf to reflect the information you provide.

Answer: We agree to provide the minimal anonymized data set as Supporting Information files for data-sharing. And Data Availability statement has been updated, and you can revise it on our behalf.

5. Your ethics statement should only appear in the Methods section of your manuscript. If your ethics statement is written in any section besides the Methods, please move it to the Methods section and delete it from any other section. Please ensure that your ethics statement is included in your manuscript, as the ethics statement entered into the online submission form will not be published alongside your manuscript.

Answer: We have moved the ethics statement to the Methods sections of manuscript.

6. Please include captions for your Supporting Information files at the end of your manuscript, and update any in-text citations to match accordingly. Please see our Supporting Information guidelines for more information: http://journals.plos.org/plosone/s/supporting-information.

Answer: We have added captions for the Supporting Information files at the end of the revised manuscript (see Page 20, line 420-421), and updated in-text citations as advised.

Response to Reviewer #1' comments:

Reviewer #1: Dear Author

Thank you for the very nice work, indeed it generated comprehensive and very informative data. The active surveillance is much appreciated. I understand that such

surveillance produced a lot of data which I believe is a big challenge to make the best out of it which you did through a very nice data presentation and analysis. In addition SARI surveillance in adult is not addressed much in the literature especially in developing areas. Moreover it seems that you described surveillance from a special geographical area characterized with unique pattern of SARI surveillance especially for the influenza B as well as the summer seasonal influenza H3 peak.

Comments:

1- The 1st letters in the title are to be capitalized.

Answer: The first letters in the title have been capitalized as advised.

Abstract

1- In the abstract line 71-73, the statement “No significant difference among … rate of main pathogens.” is unclear, please rephrase.

Answer: We have modified the statement of this sentence (see Page 3, line 62-64).

Methods

2- Line 132 please insert a reference for Sari definition.

Answer: We thank for this suggestion. A reference for SARI definition has been inserted (see Page 6, line 123).

3- Please specify details of sample collection: oropharyngeal or nasopharyngeal or both, type of the swabs used and manufacturer, VTM inhouse prepared or commercial and it’s manufacturer, duration of sample storage till transportation.

Answer: We have specified the details of sample collection including the type of swab and manufacturer. The information of VTM manufacturer and duration of sample storage till transportation have been provided as advised (see Page 7, line 142-147).

4- Please specify the type of kits used : catalogue number, manufacturer or if it is inhouse made, provide primers and reagent used along with the reference.

Answer: The information of PCR kits has been specified (see Page 7, line 154 to Page 8, line 156). Both of the primers and reagent came from the PCR kit. The testing process of PCR was conducted according to the manufacturer's protocols.

5- Study subjects: Are the patients admitted in ICU or regular wards?

Answer: The patients in this study included those admitted in ICU, respiratory medicine department and general wards, which was specified in the Study Subject section (see Page 6, line 118-119).

6- Line 158-159 "Specimens were lysed at strongly denaturing conditions to deactivate RNases" please provide a reference as I believe that harsh conditions may affect the target fragile viral RNA.

Answer: We have followed the comment, deleted the term of "strongly" and rephrased the sentence in line 158, also, a reference has been provided according to your suggestion (see Page 7, line 151-152).

7- Line 160: using term "contaminant" is incorrect

Answer: Another reviewer thought that it was unnecessary to keep the sentence which was located in line 159-160, namely, "After adding alcohol and loading lysates onto the QIAamp spin column, viral RNA and DNA combined to the QIAamp silica membrane while contaminants passed through". We followed this suggestion and deleted this sentence which included the term of "contaminant".

Results

8- Line 237: it is not clear where did these numbers came from (20/95, 21/94) and how can the P value show significant difference between these very close findings. Please recheck and clarify.

Answer: The denominator (95,94) were the total number of monitoring patients in summer(Jun-Aug) and autumn(Sep-Nov) respectively, and the numerator(20,21) were the positive number of patients in summer(Jun-Aug) and autumn(Sep-Nov) respectively. As for the P value, we are sorry for negligence. The P value should be 0.83 and the difference is not significant. Thanks for point to this mistake, we have corrected it (see Page 12, line 254).

9- Line 239 and 240 please clarify what this P value indicates.
Answer: We have clarified the significance of this P value (see Page 12, line 254-256).

Discussion
10- For the significant P values, you addressed the comorbidities in the discussion. What about the dyspnea and the radiologic examination.

Answer: We thanks for this comment. We have addressed the dyspnea and presence of radiographic diagnosis of pneumonia in the discussion (see Page 16, line 344 to Page 17, line 364).
11- Findings in the result section line 224 and 225 were not discussed regarding the Xray finding in the mycoplasma and rhino causing dyspnea.

Answer: We thanks for this comment and have discussed them accordingly (see Page 16, line 344 to Page 17, line 357).
12- In the discussion, comparison of the patients from Madagascar and yours is irrelevant as they enrolled pediatric patients that were excluded from your study.

Answer: This comment is appreciated and we deleted this comparison in the discussion.
13- Line 311: You discuss cough as being the most common symptom, this is obvious

as it in part of the inclusion criteria. Rather, you should address elaboration about the pneumonia and bronchiolitis.

Answer: We are sorry for no discussing the pneumonia in discussion on account of space limitation of original manuscript. In the revised paper, we have discussed the pneumonia and bronchiolitis following the suggestion (see Page 16, line 340-344).

Figures and tables:

14- Figure 3: Percentage of the y axis is not clear (is it from the total enrolled or from the positive cases only). Please provide your definition of the detection rate.

Answer: We have clarified the significance of y axis and provided the definition of the detection rate in Fig 2 and Fig3.

15- Table 5: please draw lines between columns as it is confusing.

Answer: We have drawn lines between columns in all 5 tables according to this comment(see Table 5).

16- Table 4: Title is not informative. Significant P values need further analysis to detect the significance is between which 2 groups.

Answer: Title of table 4 has been revised (see Page 31, line 609-610). As for 3 variables with significant P value, we conducted the pairwise comparison (see Page 32, line 611-615). Also, we revised the statistics section accordingly (see Page 9, line 177-178).

17- Table3: It is not clear what is meant by “Chest radiographic exam”, please clarify especially that it shows significant P value and should be addressed in the discussion.

Answer: It means the acceptance of chest radiographic exam, we have revised it and clarified especially in bold font in table 3. And we addressed it in the discussion (see Page 17, line 357-364).

18- In table 2 : Percent is done from the total enrolled cases or from the positive ones. Please clarify and add the total number at the end.

Answer: Percent refers to the frequency of positive etiology divided by the total enrolled samples (397 cases). We have provided the explanation for it under the table 2 and added the total number at the end (see Page 29, line 590-591).

GENERAL:

19- Please specify that the surveillance addresses the community acquired infections.

Answer: We have specified this important significance of surveillance system in the Background section (see Page 5, line 99-101).

20- When you mention “Presence of radiographic diagnosis of pneumonia” you mean, lobar pneumonia denoting mostly bacterial origin, or atypical pneumonia denoting viral or atypical bacterial origin (Mycoplasma). These details need to be mentioned especially for the negative cases as they may indicate other non-tested bacterial etiology.

Answer: We are sorry that our case report form is the standard structural questionnaire, and it just collected the result whether has the presence of radiographic diagnosis of pneumonia, and can not show lobar pneumonia or atypical pneumonia. Meanwhile, the pathogens tested in this piloting study only covered common respiratory viruses and *Mycoplasma pneumonia*, and did not include respiratory bacterium. We agreed this comment and we address it in the limitation section (see Page 19, line 400-408).

21- Some sentences are ambiguous and need to be rephrased or corrected:

a. Line 149

Answer: The sentence in line 149 has been revised (see Page 7, line 139-140).

b. Line 188: remove “positive”

Answer: The term of “positive” in line 188 has been removed (see Page 9, line 190).

c. Line 191

Answer: The sentence in line 191 has been revised (see Page 9, line 194-195).

d. Line 273-274

Answer: The sentence in line 273-274 has been revised (see Page 14, line 295-297).

e. Line 295

Answer: The previous comment thought the sentence in line 295 did not hold, so we delete this sentence in line295-298.

f. Line 323

Answer: The “viral respiratory SARI” in line 323 has been changed to “viral SARI” (see Page 18, line 376).

g. Line 341

Answer: The sentence in line 341 has been revised (see Page 19, line 397-398).

h. Line 345

Answer: The sentence in line 345 has been revised (see Page 19, line 404-407).

Recommendations:

1- The title include many details that can be removed as the age group and the study period

Answer: We deleted the study period (April 2017 to March 2018) from the title following the recommendation. Meanwhile, we respect the editor’s suggestion about

this point. Since SARI surveillance in adults is not addressed much in the literatures especially in developing areas, we think it'd better to keep 'adult' in the title to show the difference from other studies.

2- Seasonality is better described in Epidemiologic weeks (Epi-weeks)

Answer: We respect this recommendation and it is accepted that seasonality can be described in both weeks and months. Some studies about SARI surveillance described seasonality in months, such as reference of 10 and 20. Also , our piloting study only last for 12 months and did not include enough patients. In the case of relatively small sample size of patients with confirmed pathogens, the use of weeks will make the seasonality character can not be better displayed. So we thought it is better to describe seasonality in months in order to show the characteristics of seasonality of SARI clearly.

Response to Reviewer #2' comments:

Reviewer #2: The authors described the etiological and epidemiological characters of severe acute respiratory infection caused by multiple viruses and mycoplasma pneumoniae in adult patients in Jinshan of Shanghai, April 2017 to March 2018. So befor publication there are some points need to revise as following:-

Major questions Must be clarified:-

1- Why did the authors not represent the values of real time PCR / RT-PCR for the detected pathogens as an indicator for the load of different pathogens and if there are variations among their load in relation to seasonal variation?

Answer: The PCR kit this study used is a qualitative detection kit. The detecting results were judged by Tm value of various pathogens according to melting curve. The kit didn't provide the quantitative value for the load of different pathogens. So, we are sorry that we can't state if there are variations among loads in relation of seasonal variation. We have clarified the qualitative characteristic of PCR kit in the

manuscript following in this comment (see Page 7, line 154 to Page 8, line 156).

2- Only pathogens from males (173 positive cases) were statistically analyzed in relation to different variants such as type of pathogen, clinical and diagnostic parameters, age......etac Why did not authors do the same data analysis for female samples (77 positive cases) as in table 4? Also, Table 1 based manily on male cases (194) and no data concerning the female (203), why?

Answer: Please allow us to clarify these problems. Both of the differences between males and females for the proportions in table 4 and table 1 have been analyzed, and initially, we omitted to display the information of female patients on consideration of controlling the length of table. We have added a row to show the female information in table 1 and table 4.

3- Among 19 pathogens have been detected authors decided to focus on only 6 pathogens although other studies stated the predominance of other pathogens such as RSV?

Answer: This study detected 17 kinds of pathogens, in which the number of six pathogens exceeds 10. So we focus on these 6 main pathogens as the number of other seven pathogens all was fewer than 10. Table 2 described all detected pathogens. We have clarified this in the discussion (see Page 15, line 313-319).

Minor comments

1-The manuscript should be revised carefulley for typographical errors.

Answer: We have revised carefully for typographical error of the manuscript.

Abstract

2-abbravietions in line 71 should be defined at its first appearance as in line 66 then use the abbreviations

Answer: The names of viruses in line 71 have been defined with their full names at their first appearance (see Page 2, line 38 and Page 3, line 49-52). Other abbreviations

in the manuscript have also been checked and revised.

3-lines 66-67 only 217 pathogens reported while in line 63 they are 250, could you mention the other type of etological agent and its frequency.

Answer: 217 pathogens in lines 66-67 refers to the total frequency of 4 main pathogens, and 250 in line 63 is the total number of patients who were identified as at least 1 pathogen. We have followed this suggestion and added the other type of etiological agents and their frequency in the abstract (see Page 3, line 51-57).

Background

4-line 100:- "owing to the lack of gold standard methods to swiftly determine etiological diagnoses" change to "owing to the lack of gold standard diagnostic methods to swiftly determine etiological agents"

Answer: We thanks for this suggestion and revised this sentence accordingly(see Page 4, line 84-85).

Materials and methods

5-Line 133:- "≥38˚C, cough, with onset within the last 10 days and require hospitalization" change to "≥38˚C, cough onset within the last 10 days and require hospitalization"

Answer: We thanks for this suggestion and revised this sentence accordingly (see Page 6, line 122-123).

6-Lines 137-138:- "vaccination (vaccinating influenza vaccine during 1 year before illness onset, vaccinating pneumococcal conjugate vaccine)" change to "vaccination (receiving influenza vaccine during 1 year before illness onset,and pneumococcal conjugate vaccine)"

Answer: We thanks for this suggestion and revised this sentence accordingly (see Page 6, line 127-128).

7-Line 149:- "149 information that could identify the identification of patients was masked during or after data" change to "149 information that could identify the personality of patients was masked during or after data"

Answer: We thanks for this suggestion and revised this sentence accordingly (see Page 7, line 139-140).

8-Line 157:- "viral RNA and DNA using the QIAamp Viral RNA Mini Kit (Qiagen, Hilden, Germany) following " change to "viral RNA and DNA using the QIAamp Viral RNA/DNA Mini Kit (Qiagen, Hilden, Germany) following"

Answer: We are sorry for this negligence and revised this sentence according to the suggestion (see Page 7, line 148-150).

9-Lines 161-162:- "Total nucleic acid extracts were further processed by multiplex real-time reverse transcription" change to "Viral nucleic acid extracts were further processed by multiplex real-time reverse transcription" since you used kit for viral nucleic acid (RNA or DNA)

Answer: We thanks for this suggestion and revised this sentence accordingly(see Page 7, line 153-154).

10-Lines 163-163:- "Respiratory pathogens 15 multiplex real-time RT-PCR diagnostic strategy was adopted to detect PIV (types 1, ..." change to "The multiplex real-time RT-PCR diagnostic strategy was adopted to detect 15 respiratory pathogens, PIV (types 1, ..."

Answer: We thanks for this suggestion and revised this sentence following the suggestion (see Page 7, line 154 to Page 8, line 156).

Results

11-As general when you describe the results please make full description of the full cases either positive or not and do not leave unclear such as line 212 you mentioned 382 cases and ignored the residue 15 cases and this was repeated allover the manuiscript, do not leave anything for guessing.

Answer: We thank for this suggestion, and have tried our best to clarify these unclear descriptions all over the manuscript as advised (see Page 9, line 191-197; Page 10, line 199-200; Page 11, line 222-225; Page 13, line 277-280).

12-Lines 199-203:- Authors described the frequency and type of pathogens,however in compare to table 2 there is confusion concerning the pathogen frequency as in text 198 singl and 52 multiple, while later on the number will be 232 and in table 312, how can this occur? please clarify this.

Answer: Number of 198 and 52 in line 199 were the number of patients with single and multiple infections, respectively. Numbers from line 201 to line 203 including 95 (*M. pneumoniae*), 46 (AdV), 44 (Flu A/H3N2), 32 (HRhV), 25 (Flu B/Yamagata) represent the frequency of identified pathogen which was detected most frequently, and their meaning was different from that in line 199.Numbers from the 3rd row(16 for Flu A/pH1N1) to the 25th row(95 for *M. pneumoniae*) in table 2 also represent the frequency of identified pathogens and their total number equals to 312. We have revised the corresponding description in section of etiology (see Page 10, line 206-214), and added the explanation for frequency under the table 2.

13-lines 213-215:- "Thirty-two SARI patients and 30 patients had exposure of contacting with patients with fever and respiratory symptoms and contacting with live poultry during 2 weeks before their illness onset, respectively" change to "Thirty-two SARI patients had exposures with fever and respiratory symptoms patients while 30 SARI patients contacted with live poultry during 2 weeks before their illness onset"

Answer: We thanks for this suggestion and revised this sentence following the suggestion (see Page 11, line 225-227).

Tables

1- Table 1 1st row change " All SARI SARI patient with confirmed pathogens SARI patient without confirmed pathogens" to "All with confirmed pathogens without confirmed pathogens" and add SARI patient above as another row.

Answer: We have revised the 1st row of Table 1 and added SARI patient above as another row following this suggestion (see Table1).

2- Table 2 1st clonumn please change "viral etiology" to "etiology" only because there is a bacteria also mentioned there.

Answer: We are sorry for this negligence and have changed it according to the suggestion (see Table 2).

3- Table 3 1st row change " All SARI SARI patient with confirmed pathogens SARI patient without confirmed pathogens" to "All with confirmed pathogens without confirmed pathogens" and add SARI patient above as another row. Visiting a live poultry market and Contact with live poultry in table 3 looks the same where in table 4 it become one catogery Contact with live poultry.

Answer: We have revised the 1st row of Table 3 and added SARI patient above as another row following this suggestion, so does the Table 5. Contact with live poultry included contacting with live poultry at home and other place (such as live poultry market), so it is different from visiting a live poultry market. Since the number of patients visiting a live poultry market was just 3 cases, and it only included 1 case with single-infected *M. pneumoniae* positivity and 1 case with single-infected AdV positivity, the third case belonged to multiple infections, so the initial table 4 didn't analyze this variable. We have analyzed it in table 4 according to this comment (see Table 4).

Figures

The presented pathogens in Fig 1-3 based only on male SARI cases with confirmed pathogens or included all pathogens from male and female cases.

Answer: The pathogens in Fig1-3 based on all SARI cases with confirmed pathogens including male and female cases.
