# Supplementary material for: Etiological and epidemiological characteristics of severe acute respiratory infection caused by multiple viruses and Mycoplasma pneumoniae in adult patients in Jinshan, Shanghai: A pilot hospital-based surveillance study
Source: PLoS One. 2021 Mar 22;16(3):e0248750. doi: 10.1371/journal.pone.0248750 (PMC7984646; doi:10.1371/journal.pone.0248750)
Supplement: Attachment — Submitted filename: Response to Reviewers.docx [file pone.0248750.s005.docx]

January 5, 2020

Dear editor,

On behalf of my co-authors, we are very appreciated to know that our manuscript (PONE-D-20-19561) is potentially acceptable for publication in PLOS ONE. We thank editors and reviewers greatly for their positive comments on our manuscript. These comments are greatly helpful in improving our manuscript and are addressed carefully. We made corresponding revisions to the manuscript according to comments. The revised manuscript highlights changes made to the original version with red color. Also, we provide a point-by-point response to each comment. The revised manuscript has been polished by a professional, native English speaker from Springer Nature for language usage, spelling, and grammar. In addition, we agree to provide the minimal data set underlying the findings as Supporting Information files for data-sharing.

We believe that the revised version of manuscript is improved highly and attached please find the revised manuscript. We ensure that our manuscript has conformed to the journal style, and we confirm that all author details on the revised version are correct, that all authors have agreed to authorship and order of authorship for this manuscript. We hope the manuscript will receive your kind consideration and be published in your valuable journal.

We would like to express our great appreciation to you and reviewers for comments on our manuscript. Looking forward to hearing from you.

Best regards.

Yours sincerely,

Jian Li

**Response to Specific Comments:**

1. Specimen collection and laboratory testing: This section need further clarification. Please specify the multiplex PCR used. Did the authors used method described in previous literatures or commercial kit?

**Answer:** We thank for these suggestions and have made further clarification. The multiplex PCR used is the commercial kit. We added the data about multiplex PCR and made further clarification (see Page 7, line 154 to Page 8, line 156 in **Revised Manuscript with Track Changes, the same below**).

Line 219 – 223: Not sure what the authors wish to convey, please rephrase for clarification.

**Answer:** We are sorry and have rephrased these sentences (see Page 11, line232-236).

2. Line 260 – 265:  Not clear on what the authors’ intention on these statement, please clarify.

**Answer:** These sentences in line 260-265 mean to show that there were no significant differences of therapy between SARI patients with confirmed pathogen and those without confirmed pathogen. We have modified our text as advised (see Page 13, line 280-286).

3. Line 295 – 299: This argument does not hold.  These are not fair comparison since this study excluded children.

Answer: This comment is appreciated highly. We deleted these sentences in line 295-298 following this comment, and revised the next sentence in line 298-299(see Page 15, line321-323).

4. Ethical statement:  This needs to be included in the Materials and Methods section and needs to include approval number.

**Answer:** The ethical statement has been moved to the Materials and Methods section, and the approval number has been added (see Page 9, line 181-186).

**Response to Journal Requirements:**

1. Please ensure that your manuscript meets PLOS ONE's style requirements, including those for file naming. The PLOS ONE style templates can be found at

https://journals.plos.org/plosone/s/file?id=wjVg/PLOSOne_formatting_sample_main_body.pdf and

https://journals.plos.org/plosone/s/file?id=ba62/PLOSOne_formatting_sample_title_authors_affiliations.pdf

**Answer:** We ensure that our manuscript meets the journal’s style.

2. In your methods and ethics statement, please state whether you obtained consent from parents or guardians of minors under 18 years old.

**Answer:** We have stated that the consent from parents or guardians of those under 18 years old have been obtained in the section of “ethics statement” (see Page 9, line 184-186).

3. PLOS ONE requires experimental methods to be described in enough detail to allow suitably skilled investigators to fully replicate and evaluate your study. See https://journals.plos.org/plosone/s/submission-guidelines#loc-materials-and-methods for more information.
To comply with PLOS ONE submission guidelines, in your Methods section, please provide a more detailed description of your methodology, specifically about your respiratory pathogens 15 multiplex real-time RT-PCR, Flu A/B RT-PCR, and flu typing methods.

**Answer:** We have provided a more detailed description of methodology in the section of specimen collection and laboratory testing as advised (see Page 7, line 142 to Page 8, line 166).

4.  We note that you have indicated that data from this study are available upon request. PLOS only allows data to be available upon request if there are legal or ethical restrictions on sharing data publicly. For information on unacceptable data access restrictions, please see http://journals.plos.org/plosone/s/data-availability#loc-unacceptable-data-access-restrictions.

In your revised cover letter, please address the following prompts:

a) If there are ethical or legal restrictions on sharing a de-identified data set, please explain them in detail (e.g., data contain potentially identifying or sensitive patient information) and who has imposed them (e.g., an ethics committee). Please also provide contact information for a data access committee, ethics committee, or other institutional body to which data requests may be sent.

b) If there are no restrictions, please upload the minimal anonymized data set necessary to replicate your study findings as either Supporting Information files or to a stable, public repository and provide us with the relevant URLs, DOIs, or accession numbers. Please see http://www.bmj.com/content/340/bmj.c181.long for guidelines on how to de-identify and prepare clinical data for publication. For a list of acceptable repositories, please see http://journals.plos.org/plosone/s/data-availability#loc-recommended-repositories.

We will update your Data Availability statement on your behalf to reflect the information you provide.

**Answer:** We agree to provide the minimal anonymized data set as Supporting Information files for data-sharing. And Data Availability statement has been updated, and you can revise it on our behalf.

5. Your ethics statement should only appear in the Methods section of your manuscript. If your ethics statement is written in any section besides the Methods, please move it to the Methods section and delete it from any other section. Please ensure that your ethics statement is included in your manuscript, as the ethics statement entered into the online submission form will not be published alongside your manuscript.

**Answer:** We have moved the ethics statement to the Methods sections of manuscript.

6. Please include captions for your Supporting Information files at the end of your manuscript, and update any in-text citations to match accordingly. Please see our Supporting Information guidelines for more information: [http://journals.plos.org/plosone/s/supporting-information](about:blank).

**Answer:** We have added captions for the Supporting Information files at the end of the revised manuscript (see Page 20, line 420-421), and updated in-text citations as advised.

**Response to Reviewer #1' comments:**

**Reviewer #1: Dear Author**
Thank you for the very nice work, indeed it generated comprehensive and very informative data. The active surveillance is much appreciated. I understand that such surveillance produced a lot of data which I believe is a big challenge to make the best out of it which you did through a very nice data presentation and analysis. In addition SARI surveillance in adult is not addressed much in the literature especially in developing areas. Moreover it seems that you described surveillance from a special geographical area characterized with unique pattern of SARI surveillance especially for the influenza B as well as the summer seasonal influenza H3 peak.

**Comments:**
1- The 1st letters in the title are to be capitalized.
**Answer:** The first letters in the title have been capitalized as advised.

Abstract
1- In the abstract line 71-73, the statement “No significant difference among … rate of main pathogens.” is unclear, please rephrase.
**Answer:** We have modified the statement of this sentence (see Page 3, line 62-64).

Methods
2- Line 132 please insert a reference for Sari definition.

**Answer:** We thank for this suggestion. A reference for SARI definition has been inserted (see Page 6, line 123).

3- Please specify details of sample collection: oropharyngeal or nasopharyngeal or both, type of the swabs used and manufacturer, VTM inhouse prepared or commercial and it’s manufacturer, duration of sample storage till transportation.

**Answer:** We have specified the details of sample collection including the type of swab and manufacturer. The information of VTM manufacturer and duration of sample storage till transportation have been provided as advised (see Page 7, line 142-147).
4- Please specify the type of kits used : catalogue number, manufacturer or if it is inhouse made, provide primers and reagent used along with the reference.

**Answer:** The information of PCR kits has been specified (see Page 7, line 154 to Page 8, line 156). Both of the primers and reagent came from the PCR kit. The testing process of PCR was conducted according to the manufacturer’s protocols.
5- Study subjects: Are the patients admitted in ICU or regular wards?

**Answer:** The patients in this study included those admitted in ICU, respiratory medicine department and general wards, which was specified in the Study Subject section (see Page 6, line 118-119).
6- Line 158-159 “Specimens were lysed at strongly denaturing conditions to deactivate RNases” please provide a reference as I believe that harsh conditions may affect the target fragile viral RNA.

**Answer:** We have followed the comment, deleted the term of “strongly” and rephrased the sentence in line 158, also, a reference has been provided according to your suggestion (see Page 7, line 151-152).
7- Line 160: using term “contaminant” is incorrect

**Answer:** Another reviewer thought that it was unnecessary to keep the sentence which was located in line 159-160, namely, “After adding alcohol and loading lysates onto the QIAamp spin column, viral RNA and DNA combined to the QIAamp silica membrane while contaminants passed through”. We followed this suggestion and deleted this sentence which included the term of “contaminant”.


Results
8- Line 237: it is not clear where did these numbers came from (20/95, 21/94) and how can the P value show significant difference between these very close findings. Please recheck and clarify.

**Answer:** The denominator (95,94) were the total number of monitoring patients in summer(Jun-Aug) and autumn(Sep-Nov) respectively, and the numerator(20,21) were the positive number of patients in summer(Jun-Aug) and autumn(Sep-Nov) respectively. As for the P value, we are sorry for negligence. The P value should be 0.83 and the difference is not significant. Thanks for point to this mistake, we have corrected it (see Page 12, line 254).

9- Line 239 and 240 please clarify what this P value indicates.
**Answer:** We have clarified the significance of this P value (see Page 12, line 254-256).

Discussion
10- For the significant P values, you addressed the comorbidities in the discussion. What about the dyspnea and the radiologic examination.

**Answer:** We thanks for this comment. We have addressed the dyspnea and presence of radiographic diagnosis of pneumonia in the discussion (see Page 16, line 344 to Page 17, line 364).
11- Findings in the result section line 224 and 225 were not discussed regarding the Xray finding in the mycoplasma and rhino causing dyspnea.

**Answer:** We thanks for this comment and have discussed them accordingly (see Page 16, line 344 to Page 17, line 357).
12- In the discussion, comparison of the patients from Madagascar and yours is irrelevant as they enrolled pediatric patients that were excluded from your study.

**Answer:** This comment is appreciated and we deleted this comparison in the discussion.
13- Line 311: You discuss cough as being the most common symptom, this is obvious as it in part of the inclusion criteria. Rather, you should address elaboration about the pneumonia and bronchiolitis.

**Answer:** We are sorry for no discussing the pneumonia in discussion on account of space limitation of original manuscript. In the revised paper, we have discussed the pneumonia and bronchiolitis following the suggestion (see Page 16, line 340-344).

Figures and tables:
14- Figure 3: Percentage of the y axis is not clear (is it from the total enrolled or from the positive cases only). Please provide your definition of the detection rate.

**Answer:** We have clarified the significance of y axis and provided the definition of the detection rate in Fig 2 and Fig3.
15- Table 5: please draw lines between columns as it is confusing.

**Answer:** We have drawn lines between columns in all 5 tables according to this comment(see Table 5).

16- Table 4: Title is not informative. Significant P values need further analysis to detect the significance is between which 2 groups.

**Answer:** Title of table 4 has been revised (see Page 31, line 609-610). As for 3 variables with significant P value, we conducted the pairwise comparison (see Page 32, line 611-615). Also, we revised the statistics section accordingly (see Page 9, line 177-178).

17- Table3: It is not clear what is meant by “Chest radiographic exam”, please clarify especially that it shows significant P value and should be addressed in the discussion.

**Answer:** It means the acceptance of chest radiographic exam, we have revised it and clarified especially in bold font in table 3. And we addressed it in the discussion (see Page 17, line 357-364).

18- In table 2 : Percent is done from the total enrolled cases or from the positive ones. Please clarify and add the total number at the end.

**Answer:** Percent refers to the frequency of positive etiology divided by the total enrolled samples (397 cases). We have provided the explanation for it under the table 2 and added the total number at the end (see Page 29, line 590-591).

**GENERAL:**
19- Please specify that the surveillance addresses the community acquired infections.

**Answer:** We have specified this important significance of surveillance system in the Background section (see Page 5, line 99-101).
20- When you mention “Presence of radiographic diagnosis of pneumonia” you mean, lobar pneumonia denoting mostly bacterial origin, or atypical pneumonia denoting viral or atypical bacterial origin (Mycoplasma). These details need to be mentioned especially for the negative cases as they may indicate other non-tested bacterial etiology.

**Answer:** We are sorry that our case report form is the standard structural questionnaire, and it just collected the result whether has the presence of radiographic diagnosis of pneumonia, and can not show lobar pneumonia or atypical pneumonia. Meanwhile, the pathogens tested in this piloting study only covered common respiratory viruses and *Mycoplasma pneumonia*, and did not include respiratory bacterium. We agreed this comment and we address it in the limitation section (see Page 19, line 400-408).
21- Some sentences are ambiguous and need to be rephrased or corrected:
a. Line 149

**Answer:** The sentence in line 149 has been revised (see Page 7, line 139-140).
b. Line 188: remove “positive”

**Answer:** The term of “positive” in line 188 has been removed (see Page 9, line 190).
c. Line 191

**Answer:** The sentence in line 191 has been revised (see Page 9, line 194-195).
d. Line 273-274

**Answer:** The sentence in line 273-274 has been revised (see Page 14, line 295-297).
e. Line 295

**Answer:** The previous comment thought the sentence in line 295 did not hold, so we delete this sentence in line295-298.
f. Line 323

**Answer:** The “viral respiratory SARI” in line 323 has been changed to “viral SARI” (see Page 18, line 376).
g. Line 341

**Answer:** The sentence in line 341 has been revised (see Page 19, line 397-398).
h. Line 345
**Answer:** The sentence in line 345 has been revised (see Page 19, line 404-407).

Recommendations:
1- The title include many details that can be removed as the age group and the study period

**Answer:** We deleted the study period (April 2017 to March 2018) from the title following the recommendation. Meanwhile, we respect the editor’s suggestion about this point. Since SARI surveillance in adults is not addressed much in the literatures especially in developing areas, we think it’d better to keep ‘adult’ in the title to show the difference from other studies.
2- Seasonality is better described in Epidemiologic weeks (Epi-weeks)

**Answer:** We respect this recommendation and it is accepted that seasonality can be described in both weeks and months. Some studies about SARI surveillance described seasonality in months, such as reference of 10 and 20. Also , our piloting study only last for 12 months and did not include enough patients. In the case of relatively small sample size of patients with confirmed pathogens, the use of weeks will make the seasonality character can not be better displayed. So we thought it is better to describe seasonality in months in order to show the characteristics of seasonality of SARI clearly.

**Response to Reviewer #2' comments:**

**Reviewer #2:** The authors described the etiological and epidemiological characters of severe acute respiratory infection caused by multiple viruses and mycoplasma pneumoniae in adult patients in Jinshan of Shanghai, April 2017 to March 2018. So befor publication there are some points need to revise as following:-

Major questions Must be clarified:-
1- Why did the authors not represent the values of real time PCR / RT-PCR for the detected pathogens as an indicator for the load of different pathogens  and if there are variations among their load in relation to seasonal variation?

**Answer:** The PCR kit this study used is a qualitative detection kit. The detecting results were judged by Tm value of various pathogens according to melting curve. The kit didn’t provide the quantitative value for the load of different pathogens. So, we are sorry that we can’t state if there are variations among loads in relation of seasonal variation. We have clarified the qualitative characteristic of PCR kit in the manuscript following in this comment (see Page 7, line 154 to Page 8, line 156).
2- Only pathogens from males (173 positive cases) were statistically analyzed in relation to different variants such as type of pathogen, clinical and diagnostic parameters, age......etac Why did not authors do the same data analysis for female samples (77 positive cases) as in table 4? Also, Table 1 based manily on male cases (194) and no data concerning the female (203), why?

**Answer:** Please allow us to clarify these problems. Both of the differences between males and females for the proportions in table 4 and table 1 have been analyzed, and initially, we omitted to display the information of female patients on consideration of controlling the length of table. We have added a row to show the female information in table 1 and table 4.
3- Among 19 pathogens have been detected authors decided to focus on only 6 pathogens although other studies stated the predominance of other pathogens such as RSV?

**Answer:** This study detected 17 kinds of pathogens, in which the number of six pathogens exceeds 10. So we focus on these 6 main pathogens as the number of other seven pathogens all was fewer than 10. Table 2 described all detected pathogens. We have clarified this in the discussion (see Page 15, line 313-319).

Minor comments
1-The manuscript should be revised carefulley for typographical errors.

**Answer:** We have revised carefully for typographical error of the manuscript.
Abstract
2-abbravietions in line 71 should be defined at its first appearance as in line 66 then use the abbreviations

**Answer:** The names of viruses in line 71 have been defined with their full names at their first appearance (see Page 2, line 38 and Page 3, line 49-52). Other abbreviations in the manuscript have also been checked and revised.
3-lines 66-67 only 217 pathogens reported while in line 63 they are 250, could you mention the other type of etological agent and its frequency.
**Answer:** 217 pathogens in lines 66-67 refers to the total frequency of 4 main pathogens, and 250 in line 63 is the total number of patients who were identified as at least 1 pathogen. We have followed this suggestion and added the other type of etiological agents and their frequency in the abstract (see Page 3, line 51-57).
Background
4-line 100:- "owing to the lack of gold standard methods to swiftly determine etiological diagnoses" change to "owing to the lack of gold standard diagnostic methods to swiftly determine etiological agents"
**Answer:** We thanks for this suggestion and revised this sentence accordingly(see Page 4, line 84-85).
Materials and methods
5-Line 133:- "≥38˚C, cough, with onset within the last 10 days and require hospitalization" change to "≥38˚C, cough onset within the last 10 days and require hospitalization"

**Answer:** We thanks for this suggestion and revised this sentence accordingly (see Page 6, line 122-123).
6-Lines 137-138:- "vaccination (vaccinating influenza vaccine during 1 year before illness onset, vaccinating pneumococcal conjugate vaccine)" change to "vaccination (receiving influenza vaccine during 1 year before illness onset,and pneumococcal conjugate vaccine)"

**Answer:** We thanks for this suggestion and revised this sentence accordingly (see Page 6, line 127-128).
7-Line 149:- "149 information that could identify the identification of patients was masked during or after data" change to "149 information that could identify the personality of patients was masked during or after data"

**Answer:** We thanks for this suggestion and revised this sentence accordingly (see Page 7, line 139-140).
8-Line 157:- "viral RNA and DNA using the QIAamp Viral RNA Mini Kit (Qiagen, Hilden, Germany) following " change to "viral RNA and DNA using the QIAamp Viral RNA/DNA Mini Kit (Qiagen, Hilden, Germany) following"

**Answer:** We are sorry for this negligence and revised this sentence according to the suggestion (see Page 7, line 148-150).
9-Lines 161-162:- "Total nucleic acid extracts were further processed by multiplex real-time reverse transcription" change to "Viral nucleic acid extracts were further processed by multiplex real-time reverse transcription" since you used kit for viral nucleic acid (RNA or DNA)

**Answer:** We thanks for this suggestion and revised this sentence accordingly(see Page 7, line 153-154).
10-Lines 163-163:- "Respiratory pathogens 15 multiplex real-time RT-PCR diagnostic strategy was adopted to detect PIV (types 1, ......." change to "The multiplex real-time RT-PCR diagnostic strategy was adopted to detect 15 respiratory pathogens, PIV (types 1, ......."

**Answer:** We thanks for this suggestion and revised this sentence following the suggestion (see Page 7, line 154 to Page 8, line 156).
Results
11-As general when you describe the results please make full description of the full cases either positive or not and do not leave unclear such as line 212 you mentioned 382 cases and ignored the residue 15 cases and this was repeated allover the manuiscript, do not leave anything for guessing.

**Answer:** We thank for this suggestion, and have tried our best to clarify these unclear descriptions all over the manuscript as advised (see Page 9, line 191-197; Page 10, line 199-200; Page 11, line 222-225; Page 13, line 277-280 ).

12-Lines 199-203:- Authors described the frequency and type of pathogens,however in compare to table 2 there is confusion concerning the pathogen frequency as in text 198 singl and 52 multiple, while later on the number will be 232 and in table 312, how can this occur? please clarify this.

**Answer:** Number of 198 and 52 in line 199 were the number of patients with single and multiple infections, respectively. Numbers from line 201 to line 203 including 95 (*M*. *pneumoniae*), 46 (AdV), 44 (Flu A/H3N2), 32 (HRhV), 25 (Flu B/Yamagata) represent the frequency of identified pathogen which was detected most frequently, and their meaning was different from that in line 199.Numbers from the 3^rd^ row( 16 for Flu A/pH1N1) to the 25^th^ row(95 for *M*. *pneumoniae*) in table 2 also represent the frequency of identified pathogens and their total number equals to 312. We have revised the corresponding description in section of etiology (see Page 10, line 206-214), and added the explanation for frequency under the table 2.

13-lines 213-215:- "Thirty-two SARI patients and 30 patients had exposure of contacting with patients with fever and respiratory symptoms and contacting with live poultry during 2 weeks before their illness onset, respectively" change to "Thirty-two SARI patients had exposures with fever and respiratory symptoms patients while 30 SARI patients contacted with live poultry during 2 weeks before their illness onset"

**Answer:** We thanks for this suggestion and revised this sentence following the suggestion (see Page 11, line 225-227).
Tables
1- Table 1 1st row change " All SARI SARI patient with confirmed pathogens SARI patient without confirmed pathogens" to "All with confirmed pathogens without confirmed pathogens" and add SARI patient above as another row.

**Answer:** We have revised the 1^st^ row of Table 1 and added SARI patient above as another row following this suggestion (see Table1).
2- Table 2 1st clonumn please change "viral etiology" to "etiology" only because there is a bacteria also mentioned there.

**Answer:** We are sorry for this negligence and have changed it according to the suggestion (see Table 2).
3- Table 3 1st row change " All SARI SARI patient with confirmed pathogens SARI patient without confirmed pathogens" to "All with confirmed pathogens without confirmed pathogens" and add SARI patient above as another row. Visiting a live poultry market and Contact with live poultry in table 3 looks the same where in table 4 it become one catogery Contact with live poultry.

**Answer:** We have revised the 1^st^ row of Table 3 and added SARI patient above as another row following this suggestion, so does the Table 5. Contact with live poultry included contacting with live poultry at home and other place (such as live poultry market), so it is different from visiting a live poultry market. Since the number of patients visiting a live poultry market was just 3 cases, and it only included 1 case with single-infected *M. pneumoniae* positivity and 1 case with single-infected AdV positivity, the third case belonged to multiple infections, so the initial table 4 didn’t analyze this variable. We have analyzed it in table 4 according to this comment (see Table 4).
Figures
The presented pathogens in Fig 1-3 based only on male SARI cases with confirmed pathogens or included all pathogens from male and female cases.

**Answer:** The pathogens in Fig1-3 based on all SARI cases with confirmed pathogens including male and female cases.
