# Supplementary material for: Etiological and epidemiological characteristics of severe acute respiratory infection caused by multiple viruses and Mycoplasma pneumoniae in adult patients in Jinshan, Shanghai: A pilot hospital-based surveillance study
Source: PLoS One. 2021 Mar 22;16(3):e0248750. doi: 10.1371/journal.pone.0248750 (PMC7984646; doi:10.1371/journal.pone.0248750)
Supplement: Attachment — Submitted filename: Response to Reviewers.docx [file pone.0248750.s008.docx]

March 1, 2021

Dear editor,

On behalf of my co-authors, we are very appreciated to know that our manuscript (PONE-D-20-19561) is potentially acceptable for publication in PLOS ONE. We thank editors and reviewers greatly for their positive comments on our manuscript. These comments are greatly helpful in improving our manuscript and are addressed carefully. We made corresponding revisions to the manuscript according to comments. The revised manuscript highlights changes made to the original version with red color. Also, we provide a point-by-point response to each comment. The revised manuscript has been thoroughly copyedited for language usage, spelling, and grammar.

We believe that the revised version of manuscript is improved highly and attached please find the revised manuscript. We ensure that our manuscript has conformed to the journal style, and we confirm that all authors have agreed to authorship and order of authorship for this manuscript. We hope the manuscript will receive your kind consideration and be published in your valuable journal.

We would like to express our great appreciation to you and reviewers for comments on our manuscript. Looking forward to hearing from you.

Best regards.

Yours sincerely,

Jian Li

Ruijin Hospital, Shanghai Jiao Tong University School of Medicine

**Response to Reviewer #1' comments:**

Reviewer #1: Thank you for the corrections made, some comments are still remaining

1.Comment #2: a reference from the WHO is expected as mentioned in the manuscript

**Answer:** We thank for this suggestion. A reference from the WHO has been replaced (see Page 6, line 114 in Revised Manuscript with Track Changes, the same below).

2.Comment #6 :as to ensure reproducibility, you can mention as per the manufacturer's instructions or otherwise specify any other steps not present in the manufacturer's protocol. The reference mentioned does not provide details of the lysis steps
**Answer:** This comment was highly appreciated. Actually, this laboratory testing was performed as per the manufacturer’s instructions. We have clarified it (see Page 7, line 141-142).

3.Comment 16#: Further statistical analysis was done for 2 out of the 3 significant P values (remaining is the comorbidity item)

**Answer:** We are sorry for negligence. The pairwise comparison for comorbidity item has been added (see Page 30, line 560-561).

**Response to Reviewer #2' comments:**

Reviewer #2: Dear Author,
Thank you for making the manuscript beautiful and clean, although minor errors or typos are still present. Please check carefully the attached file and correct these typos.

**Answer:** We highly thank for your carefulness, and we have carefully copyedited the minor errors or typos.
